# One- vs two-phase extraction: re-evaluation of sample preparation procedures for untargeted lipidomics in plasma samples

**DOI:** 10.1007/s00216-018-1200-x

**Published:** 2018-07-02

**Authors:** Andres Gil, Wenxuan Zhang, Justina C. Wolters, Hjalmar Permentier, Theo Boer, Peter Horvatovich, M. Rebecca Heiner-Fokkema, Dirk-Jan Reijngoud, Rainer Bischoff

**Affiliations:** 1 Department of Analytical Biochemistry, University of Groningen, Antonius Deusinglaan 1, 9713 AV Groningen, The Netherlands; 2 Systems Medicine and Metabolic Signaling, Department of Pediatrics, University Medical Center Groningen, University of Groningen, PO Box 30.001, 9700 RB Groningen, The Netherlands; 3 Interfaculty Mass Spectrometry Center, University of Groningen, Antonius Deusinglaan 1, 9713 AV Groningen, The Netherlands; 4 Laboratory of Metabolic Disease, Department of Laboratory Medicine, University Medical Center Groningen, University of Groningen, PO Box 30.001, 9700 RB Groningen, The Netherlands

**Keywords:** Lipids, Untargeted lipidomics, Extraction, LC-MS, Multivariate analysis

## Abstract

Lipidomics is a rapidly developing field in modern biomedical research. While LC-MS systems are able to detect most of the known lipid classes in a biological matrix, there is no single technique able to extract all of them simultaneously. In comparison with two-phase extractions, one-phase extraction systems are of particular interest, since they decrease the complexity of the experimental procedure. By using an untargeted lipidomics approach, we explored the differences/similarities between the most commonly used two-phase extraction systems (Folch, Bligh and Dyer, and MTBE) and one of the more recently introduced one-phase extraction systems for lipid analysis based on the MMC solvent mixture (MeOH/MTBE/CHCl_3_). The four extraction methods were evaluated and thoroughly compared against a pooled extract that qualitatively and quantitatively represents the average of the combined extractions. Our results show that the lipid profile obtained with the MMC system displayed the highest similarity to the pooled extract, indicating that it was most representative of the lipidome in the original sample. Furthermore, it showed better extraction efficiencies for moderate and highly apolar lipid species in comparison with the Folch, Bligh and Dyer, and MTBE extraction systems. Finally, the technical simplicity of the MMC procedure makes this solvent system highly suitable for automated, untargeted lipidomics analysis.

**Electronic supplementary material:**

The online version of this article (10.1007/s00216-018-1200-x) contains supplementary material, which is available to authorized users.

## Introduction

Several studies have shown that in addition to roles in cellular membranes and the provision of energy, lipids also have important bioactivities and signaling functions that may be altered in widespread human diseases, including cardiovascular disease, diabetes type 2, Alzheimer’s disease, and cancer [1, 2]. Consequently, lipidomics is a rapidly developing area of research mainly focused on searching biomarkers for diagnostic purposes [3, 4]. Irrespective of this rapid growth of the field and the technological advances in chromatography and mass spectrometry that resulted in the development of more sensitive, selective, and high-throughput methods over the last decade [5–8], extraction of all lipid species in a comprehensive manner remains an active area of research in the lipidomics field.

Currently, there is no single extraction technique able to extract all lipid classes from a biological matrix (tissue, biological fluid, cell) in a quantitative manner [3]. The most commonly used methods for lipid extraction were introduced by Folch et al. [9] and by Bligh and Dyer [10]. So far, different modifications of these methods have appeared for specific applications [11, 12], nevertheless, using chloroform/methanol mixtures that separate into an upper methanol-rich layer, containing hydrophilic compounds, and a lower chloroform-rich layer mainly containing lipids, remains the basis of these extractions.

An important pitfall of two-phase extractions is the high chance of contamination of the samples, due to the need of retrieving lipids from the lower chloroform-rich layer [2]. To avoid this issue, the methyl tert-butyl ether (MTBE) extraction method and more recently the butanol-methanol (BUME) method were introduced by Matyash et al. [13] and Löfgren et al. [14], respectively. While both methods have the advantage that the upper layer is the lipid-rich organic phase, unsatisfactory recovery for more polar lipid classes has been observed [14].

The main objectives of lipidomics studies are to increase the number of extracted and detected lipids (the coverage of the lipidome) and to do so in a straightforward and reproducible manner to avoid bias due to technical variability. Trying to achieve these objectives, while avoiding the inherent problems of the two-phase extraction methods, one-phase lipid extractions have recently been developed [2, 3, 15, 16]. One-phase extractions focus on an “all-in-one-tube” approach eliminating the need for phase separation by denaturing proteins that are later removed by centrifugation. Methanol, butanol, isopropanol, MTBE, and mixtures thereof have been used as solvents. However, up to date, these approaches have been evaluated with respect to the targeted analysis of a small set of lipid standards by comparing their recovery [2, 3, 14]. By using an untargeted lipidomics approach on plasma samples, the major aim of the current work is to explore the differences and similarities between the three most commonly used two-phase extraction systems and a more recently described one-phase system, the MMC solvent mixture (MeOH/MTBE/CHCl_3_) [3], for lipid analysis. The four extraction methods were evaluated and thoroughly compared against a pooled extract that qualitatively and quantitatively was considered to represent an average standard extract.

## Materials and methods

### Lipid extraction methods

All extractions were performed in 2 mL Eppendorf tubes with 75 μL of human plasma each (see the electronic Supplementary Material (ESM) for blood collection). Three samples were independently prepared for each extraction method. Incubation time (1 h) and temperature of extraction (22 °C) were kept constant. Each extraction method was performed three times (*n* = 3) on samples independently prepared and analyzed in triplicate. Two- and one-phase extractions were performed as detailed below.

### Two-phase extractions

#### Folch method (Folch)

Seventy-five microliters of human plasma was mixed with 187.4 μL of MeOH and vortexed for 20 s followed by addition of 375 μL of CHCl_3_. The mixture was incubated on a shaker at 900 rpm for 1 h. Phase separation was induced by the addition of 156.2 μL of H_2_O and incubation of the mixture for 10 min. Subsequently, the sample was centrifuged at 175,00 RCF for 10 min at 20 °C and the lower (CHCl_3_) phase was collected (300 μL). The upper MeOH phase was re-extracted with 250 μL of the following solvent mixture (CHCl_3_/MeOH/H_2_O 86:14:1, *v*/*v*/*v*), and the lower phase was again collected (250 μL). The CHCl_3_ phases were combined and dried in a vacuum centrifuge at 30 °C for 1 h. The extracted lipids were re-suspended in 50 μL CHCl_3_/MeOH/H_2_O (60:30:4.5, *v*/*v*/*v*) from which 10 μL was taken to prepare the pooled extracts (see below). The remaining 40 μL was diluted to the same level as the pooled extract (100 μL) and stored at − 20 °C.

#### Bligh and Dyer method (Bligh)

Seventy-five microliters of human plasma was mixed with 562 μL MeOH/CHCl_3_ (2:1) and vortexed for 20 s. Subsequently, the mixture was incubated on a shaker at 900 rpm for 1 h, after which, 156.2 μL of H_2_O was added to induce phase separation. The sample was centrifuged at 17,500 RCF for 10 min at 20 °C, and the lower CHCl_3_ phase was collected (150 μL). A second extraction step was performed on the upper aqueous phase with MeOH/CHCl_3_ (2:1). Both organic phases were combined and dried in a vacuum centrifuge at 30 °C for 1 h. The extracted lipids were re-suspended in 50 μL CHCl_3_/MeOH/H_2_O (60:30:4.5, *v*/*v*/*v*) from which 10 μL was taken to prepare the pooled extracts (see below). The remaining 40 μL was diluted to the same level as the pooled extract (100 μL) and stored at − 20 °C.

#### Matyash method (MTBE)

Seventy-five microliters of human plasma was mixed with 187.4 μL of MeOH and vortexed for 20 s. Next, 625 μL of MTBE was added and the mixture was incubated on a shaker at 900 rpm for 1 h. Water (156.2 μL) was added to the mixture and incubated for 10 min to induce phase separation. The sample was centrifuged at 17,500 RCF for 10 min at 20 °C, and the upper (MTBE) phase was collected (700 μL). The lower methanol phase was re-extracted with 250 μL of MTBE/MeOH/H_2_O (10:3:2.5, *v*/*v*/*v*), and the upper phase was again collected (200 μL). The MTBE phases were combined and dried in a vacuum centrifuge at 30 °C during 1 h. The extracted lipids were re-suspended in 50 μL CHCl_3_/MeOH/H_2_O (60:30:4.5) from which 10 μL was taken to prepare the pooled extracts (see below). The remaining 40 μL was diluted to the same level as the pooled extract (100 μL) and stored at − 20 °C.

In order to assess the potential loss of polar lipids, the hydrophilic phase from each two-phase extraction procedures was collected and dried under a stream of N_2_ at room temperature overnight. The pellets obtained were re-suspended separately in 40 μL CHCl_3_/MeOH/H_2_O (60:30:4.5, *v*/*v*/*v*). From each pellet, 10 μL was taken to prepare the pooled extract (see below). The remaining 30 μL was diluted to the same level as the pooled extract (100 μL) and stored at − 20 °C.

### One-phase extraction (MMC method)

Seventy-five microliters of human plasma was mixed with 500 μL of MeOH/MTBE/CHCl_3_ (1.33:1:1, *v*/*v*/*v*) and vortexed for 20 s. Subsequently, the mixture was incubated on a shaker at 900 rpm for 1 h at 22 °C. The sample was vortexed during 10 s, and particulate matter was pelleted by centrifugation at 17,500 RCF for 10 min at 20 °C. Supernatant was collected (500 μL) and dried in a vacuum centrifuge for 1 h at 30 °C. The extracted lipids were re-suspended in 50 μL chloroform/methanol/water (60:30:4.5, *v*/*v*/*v*) from which 10 μL was taken to prepare the pooled extract (see below). The remaining 40 μL was diluted to the same level as the pooled extract (100 μL) and stored at − 20 °C.

### Pooled lipid extracts

Two pooled extracts or quality control samples (QCs) containing the entire set of components from all extraction methods were prepared. The first pool consisted of the main set of extracted lipids (hydrophobic phases). Briefly, 10 μL of the extracts (in CHCl_3_/MeOH/H_2_O 60:30:4.5, *v*/*v*/*v*) from each solvent system (Folch, Bligh, MTBE, and MMC methods) was mixed. Consequently, the final volume of the hydrophobic pooled sample and the Folch, Bligh, MTBE, and MMC hydrophobic lipid extracts was 40 μL. Volumes of all five hydrophobic extracts were adjusted to 100 μL with IPA-ACN-H_2_O (2:1:1, *v*/*v*/*v*) and then subjected to LC-MS analysis.

The second pool consisted of a set of polar lipids and other components with more polar characteristics that remained in the hydrophilic phase. In short, 10 μL of the solutions (in CHCl_3_/MeOH/H_2_O 60:30:4.5, *v*/*v*/*v*) obtained from the hydrophilic phases of the two-phase extraction systems (Folch, Bligh, and MTBE) was mixed. The final volume of the hydrophilic pooled sample and that of the Folch, Bligh, and MTBE extracts was 30 μL. Final volumes were adjusted to 100 μL with IPA-ACN-H_2_O (2:1:1, *v*/*v*/*v*) and then subjected to LC-MS analysis.

### Blank extracts

In order to evaluate whether contaminant features, that might appear as lipid signals, were part of the measured lipidomes, blank extracts were obtained using water (25 μL) instead of plasma. By following the Folch, Bligh, MTBE, and MMC experimental procedures (see above), four blank extracts (*n* = 3) were obtained for comparison purposes. Contaminant features, that were found to be differentially extracted (fold change ≥ 1.5, statistical significance (*p* ≤ 0.05, Student’s *t* test), CV < 30%) in the blank extracts (both in positive and negative modes) in comparison with the various solvent systems, were removed from the data.

### LC-MS

Lipids were separated by reversed-phase chromatography using an Acquity UPLC CSH column (1.7 μm, 100 × 2.1 mm) on an Acquity UPLC system (Waters, Manchester, UK). Mobile phases consisted of 10 mM ammonium formate in water (eluent A) and 10 mM ammonium formate in methanol (eluent B). Linear gradient elution was as follows: 0–5 min from 50 to 30% eluent A, 5–15 min from 30 to 10% eluent A, and 15–25 min from 10 to 0% eluent A. This was followed by isocratic elution at 0% eluent A over the next 15 min. A conditioning cycle of 5 min with the initial proportions of eluents A and B was performed prior to the next analysis. The column temperature was set at 80 °C, and the flow rate was 0.5 mL/min. Four or eight microliters of sample was injected in positive and negative modes, respectively. The samples were analyzed in a randomized order throughout the experiment.

Mass spectrometry detection was performed using a Synapt G2-Si high-resolution QTOF mass spectrometer (Waters, Manchester, UK). Lipids were detected by electrospray ionization in positive (ESI^+^) and negative modes (ESI^−^). Nitrogen and argon were used as desolvation and collision gas, respectively. Data were acquired over the *m/z* range from 50 to 1750 Da in continuum and enhanced resolution modes, at an acquisition rate of 1 spectrum/0.2 s. The source temperature was set at 150 °C, the desolvation temperature at 400 °C, the cone voltage at 30 V, and the capillary voltage at 2000 V. MS/MS experiments were performed with data-dependent acquisition (DDA). A survey MS scan was alternated with three DDA MS/MS scans resulting in a cycle time of 1 s. Singly charged precursor ions were selected based on abundance with a threshold of 1000 cps intensity. After being selected, a particular *m*/*z* value was excluded for 30 s from MS/MS fragmentation. The collision energy potential setting was 35 V. The system was equipped with an integral LockSpray unit with its own reference sprayer that was controlled automatically by the acquisition software to collect a reference scan every 10 s lasting 0.3 s. The LockSpray internal reference used for these experiments was a 0.2-ng/μL leucine-enkephalin solution (reference mass *m/z* 556.2771 in the positive and *m/z* 554.2615 in the negative modes) infused at 10 μL/min to allow operation of the instrument at high mass accuracy (< 1 ppm).

### Data preprocessing

MassLynx software version 4.1 was used for data acquisition. Waters raw data files were analyzed using Progenesis QI software (Waters Corporation, Milford, MA) for peak alignment, peak picking, and normalization of the LC-MS data. On the basis of normalized peak intensities, the number of features was filtered according to two different sets of selection criteria (see ESM for data preprocessing). A final table containing *m*/*z* values, retention times, and normalized peak intensities was imported into Simca P v.13 (Umetrics, Umea, Sweden) for multivariate statistical analysis.

### Multivariate statistical analysis

Using Simca P v.13, data were grouped in blocks according to the extraction methods (Folch, Bligh, MTBE, and MMC), as well as to the pooled extracts (hydrophobic and hydrophilic). Principal component analysis (PCA) and partial least squares-discriminant analysis (OPLS-DA) via orthogonal projection to latent structures were carried out on the filtered features. Discriminant features between lipid profiles were identified and permutation tests were carried out to determine the robustness of the multivariate models (see ESM on multivariate statistical analysis).

### Lipid identification

An in-house data base containing retention times and accurate masses for about 600 lipid species was created by manually checking and comparing the list of lipids identified by T’Kindt et al. [17] with those present in a standard plasma sample (section on blood collection). Tentative identification of lipids was based on accurate mass determinations within a narrow *m*/*z* (1–5 mDa) and retention time (0.1 min) range. Moreover, further examination of the identified features was performed with accurate mass information present in on-line databases (LIPID MAPS, LipidBlast, and HMDB).

## Results and discussion

### Pooled lipid extracts as QC samples

The reliable multicomponent analysis of complex biological samples such as plasma by HPLC-MS presents a number of challenges with respect to obtaining valid data [18]. By exploring the time dependency of the PCA scores for pooled lipid extracts (QCs), one obtains insight into trends and drifts over the course of the analysis of a batch of samples [19]. Therefore, technical performance of the LC-MS method was monitored by randomly injecting the hydrophobic QC extract several times throughout the entire study. After conditioning the system, the pooled extract was measured nine times both in the positive and negative modes. Data were processed by PCA, and the results showed that the first principal component was within ± 2SD for both polarities, indicating that no outlier data were observed (ESM Fig. S1), as suggested elsewhere [18]. Combining aliquots of all samples to be investigated into one pooled extract to generate a QC is a common procedure in untargeted lipidomics [20]. Since the pooled extract mimics the sample matrix and lipid composition of the experimental samples both qualitatively and quantitatively, it is considered to be the average standard extract with the most comprehensive lipid composition. The pooled extract was used as a reference to test the performance of the two- and one-phase extraction methods. By using an untargeted approach with multivariate statistical data analysis, we aimed to determine whether the extraction methods produced different lipid profiles and how efficient they are for different groups of lipid species.

### Unsupervised multivariate comparison of the extraction systems

The total number of features detected in the positive mode for the hydrophobic phases followed the order: pooled > MTBE > Folch > MMC > Bligh (3688, 3300, 3254, 3200, and 3082 features, respectively). In negative ionization, the order was as follows: pooled > MTBE > MMC > Folch > Bligh (1082, 1030, 1029, 943, and 738 features, respectively). These features were filtered as described in the Experimental section (see ESM on data preprocessing) to eliminate low-intensity, highly variable signals and noise. Features fulfilling the filtering criteria were subjected to comparative multivariate statistical analysis (PCA).

**Fig. 1:**
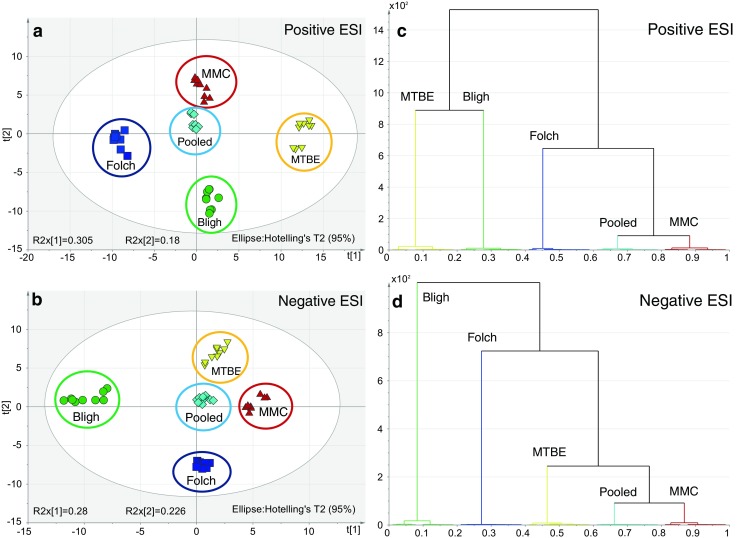
Comparison of lipid extraction methods by principal component analysis (PCA) after LC-MS analysis in positive (**A**) and negative electrospray ionization (ESI) mode (**B**). Hierarchical clustering analysis (HCA) of the same data in positive (**C**) and negative (**D**) ESI mode, depicting quantitative relationships between the extraction methods. The vertical axis of the HCA dendrograms indicates the variance increase, which can be considered normalized Euclidean distance

PCA was used to display general trends, intrinsic clustering of samples, and possible outliers. The tight clustering of the pooled extracts in the middle of both PCA score plots showed that the LC-MS analysis itself introduced little technical variability compared with the extraction methods. On data from the different extraction methods in the positive and negative ESI modes, PCA showed clear clustering of samples according to the extraction methods, indicating that different lipid profiles were acquired with the tested extraction methods (Fig. 1A, B). Since the pooled extract contains lipids derived from all four extraction methods, proximity of the cluster of a given extraction method with respect to the pool can be considered a readout of how comprehensive a given procedure is, but exact quantitative interpretation of this proximity is difficult. Therefore, hierarchical cluster analysis (HCA) was used to show the relationship between sample clusters according to similarities in lipid composition. On data obtained in the positive ESI mode, HCA showed that the MMC cluster is closest to the pool cluster followed by the Folch, the Bligh, and the MTBE clusters (Fig. 1C). In the negative ESI mode, the results show a somewhat different order of proximity but the MMC cluster is again most similar to the pool cluster. The order in the negative mode is pooled = MMC > MTBE > Folch > Bligh (Fig. 1D). According to our results, the MMC extraction method results in a lipid composition that is closest to the pooled extract from a qualitative and quantitative points of view, indicating that the lipid profile obtained with this method is most similar to the average standard extract. However, separation of clusters in the PCA plot indicates that there is still a considerable difference between the lipid profiles that needs to be considered.

### Selectivity of the extraction systems for different lipid species

We employed OPLS-DA to identify lipid species that contribute to the observed molecular profile differences between the extraction methods as observed in the PCA plots. For this analysis, a different filtering approach was used consisting of solely focusing on reproducible features by taking only the contribution of signals with a CV ≤ 30% into account. These features were then analyzed on the basis of their variable importance in the projection (VIP) scores. OPLS-DA models and S-plots were used to define those features with the greatest influence on the separation of groups (ESM Fig. S2A–H for the positive and Fig. S3A–H for the negative ESI mode). The VIP value is related to the importance of the contribution of a given variable to the model as a whole. Given that the average of the sum-of-squares of the VIP values is equal to 1, values larger than 1 indicate important variables and values lower than 0.5 indicate unimportant variables [21]. Furthermore, to check the robustness of the OPLS-DA models (pooled vs Folch, pooled vs MTBE, pooled vs Bligh, and pooled vs MMC both in the positive and negative modes), random permutation tests (*n* = 999) were performed (ESM Figs. S2A–H and S3A–H) and compared with the default cross-validation method automatically performed by the SIMCA software (see Experimental section on “Multivariate statistical analysis”). The results show that for all OPLS-DA models both in the positive and negative ESI modes, the *R*2 (> 0.983) and *Q*2 (> 0.954) values of the original models were well above the permutated models, indicating low variability and excellent predictive ability (ESM Figs. S2A–H and S3A–H).

**Fig. 2:**
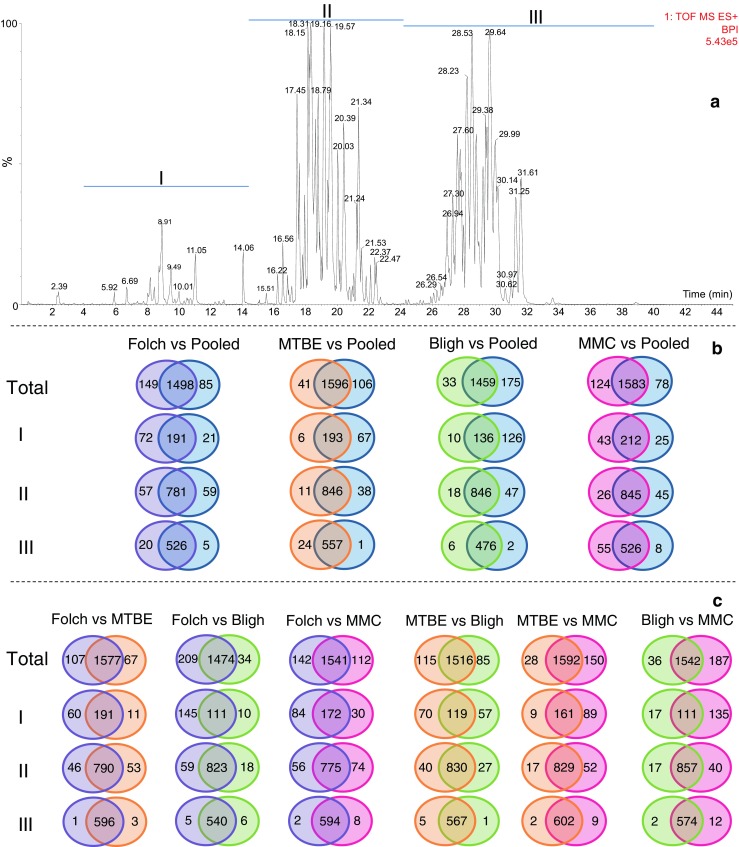
Selectivity of the extraction systems for different lipid species delimited by time windows and analyzed in positive ESI mode. (**A**) LC-MS chromatogram of lipids present in plasma samples. (**B**) Venn diagrams of the number of extracted features present in the pooled extract in comparison with the tested extraction methods. (**C**) Venn diagrams of the number of extracted features in the extracts of the tested approaches when compared with each other. The type of lipids in each segment of the chromatogram is as follows: segment I, lysophospholipids (LPL) and monoglycerides (MG); segment II, phospholipids (PI, PC, PE, and PG), sphingomyelins (SM), and diglycerides (DG); and segment III, cholesterol esters (CE) and triglycerides (TG)

**Fig. 3:**
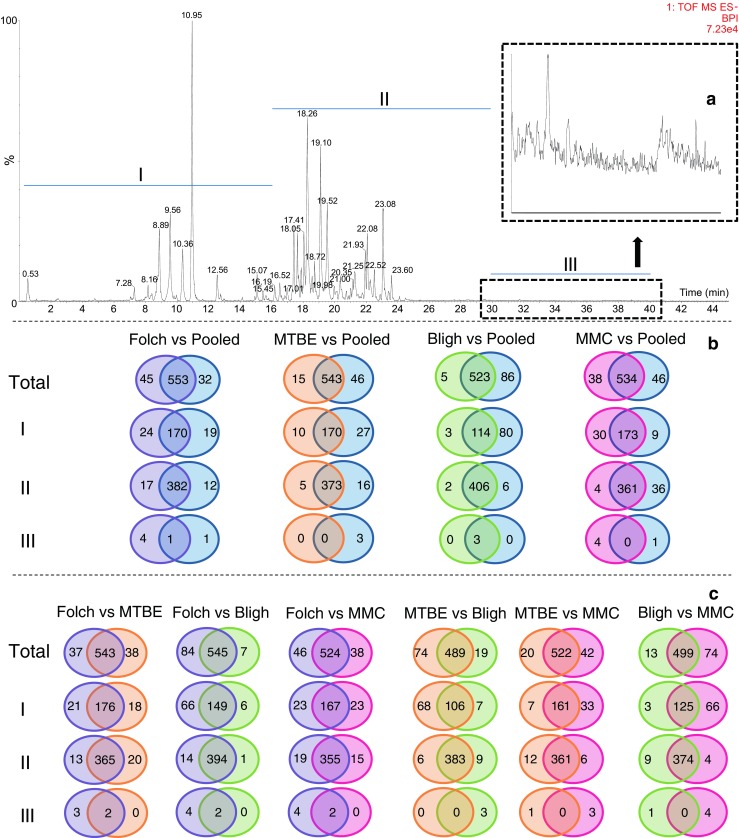
Selectivity of the extraction systems for different lipid species delimited by time windows and analyzed in negative ESI mode. (**A**) LC-MS chromatogram of lipids present in plasma samples. (**B**) Venn diagrams of the number of extracted features present in the pooled extract in comparison with the tested extraction methods. (**C**) Venn diagrams of the number of extracted features in the extracts of the tested approaches when compared with each other. The type of lipids in each segment of the chromatogram is as follows: segment I, fatty acids (FA) and lysophospholipids (LPL); segment II, phospholipids (PC, PI, PG, PS, and PE) and sphingomyelins (SM); and segment III, ceramides (Cer)

Here, we used VIP values ≥ 1.5 as cutoff, allowing a better discrimination of important features. The comparisons of features considered to be mainly responsible for discrimination between the extraction methods (VIP ≥ 1.5) are shown in Fig. 2 for the positive and Fig. 3 for the negative ESI modes, respectively. The chromatograms were divided into three different retention time segments according to decreasing polarity. In the positive mode, segment I corresponds to lysophospholipids (LPL) and monoglycerides (MG), segment II to phospholipids [phosphatidylcholines (PC), phosphatidylinositols (PI), phosphatidylglycerols (PG) and phosphatidylethanolamines (PE)], sphingomyelins (SM), cardiolipins (CL), and diglycerides (DG), and segment III to cholesterol esters (CE), cardiolipins (CL), and triglycerides (TG) (Fig. 2A). In the negative mode, segment I corresponds to fatty acids (FA) and LPL, segment II to phospholipids [PC, PI, PG, PE and phosphatidylserines (PS)] and sphingomyelins (SM), and segment III to some ceramides (Cer) (Fig. 3A). To identify a certain number of discriminating lipids, we merged the accurate mass information from three on-line databases (LIPID MAPS, LipidBlast, and HMDB) with our homemade database built on accurate mass and retention times. The class of lipid, adducts, and the identity of individual lipids in both the positive and negative ESI modes were confirmed based on matching the information using a narrow *m*/*z* window (1–5 mDa) and retention time range (0.1 min). In total, 460 distinct lipids were identified (Table 1). Glycerophospholipids were found to be the class with the largest number of species, closely followed by glycerolipids. The full list of identified lipid species and the specific groups in which the highest and lowest ion intensities were observed are shown in ESM Table S1.

**Table 1:** List of lipid classes and sub-classes identified by LC-high-resolution mass spectrometry in the extracted plasma sample

Lipid class	Number of detection	Dominant adducts	Retention time range (min)
*Fatty acyls*	**20**		
FAs	20	[M–H]^−^	6.4–16.19
*Glycerophospholipids*	**207**		
LysoPCs	32	[M+H]^+^/[M+HCOO]^−^	6.32–15.91
PCs	101	[M+H]^+^/[M+HCOO]^−^	16.42–23.83
LysoPEs	10	[M+H]^+^/[M–H]^−^	8.15–11.93
PEs	36	[M+H]^+^/[M–H]^−^	17.7–21.38
LysoPSs	1	[M–H]^−^	
PSs	5	[M–H]^−^	17.62–19.57
LysoPGs	1	[M–H]^−^	
PGs	2	[M–H]^−^	
LysoPIs	2	[M–H]^−^	
PIs	14	[M–H]^−^	16.24–18.47
CL	3	[M+H]^+^/[M+NH_4_]^+^	
*Sphingolipids*	**68**		
SMs	36	[M+H]^+^/[M+HCOO]^−^	15.04–22.96
Cers	32	[M+H]^+^/[M–H]^−^	15.69–24.17
*Glycerolipids*	**155**		
DGs	15	[M+NH_4_]^+^	19.44–22.44
TGs	140	[M+NH_4_]^+^	14.88–36.98
*Sterol lipids*	**10**		
CEs	10	[M+NH_4_]^+^	27.35–31.19
**Total**	**460**		

The Venn diagrams in Figs. 2 and 3 show that the main difference between extraction systems is due to the lipid selectivity of each solvent system. While most of the features are common to all extraction methods (Figs. 2B, C and 3B, C), there is a number of features that contributes to the formation of separate clusters upon PCA and the OPLS-DA analysis.

The main source of variation in the OPLS-DA analyses was found in the first segment of the chromatograms, comprising lipids of a polar nature (FA, LPL, and MG). In order to discard the contribution of contaminating features coming from the extraction solvents, we performed a thorough comparison between blank extracts and the tested extraction approaches and found no interferences in either the positive or negative ionization mode (ESM Figs. S5, S6, S7, and S8). In the positive ionization mode, the ratio of discriminant to common features was highest in segment I for all comparisons (pooled vs Folch, pooled vs MTBE, pooled vs Bligh, and pooled vs MMC), followed by segments II and III (Figs. 2B, C and 3B, C). In the negative mode, behavior was similar as in the positive ESI mode, implying that overall LPL, FA, and MG are strongly affected and PC, PI, PG, PS, PE, SM, and DG somewhat less by the extraction method. However, segment III in the negative ESI mode can be neglected, since the number of extracted features is rather low. Regarding comparison of the extraction methods against the pooled extract in the positive ESI mode, MMC showed the best results providing the broadest coverage across all lipid classes, followed by the Folch, MTBE, and Bligh extraction methods (1707, 1647, 1637, and 1492 extracted features, respectively). These results are in agreement with Reis et al. [12] who reported the same decreasing order in efficiency for the two-phase extraction systems (Folch > MTBE > Bligh) but contrasts with a more recent report in which the Bligh extraction system was the most efficient in the positive ESI mode [22]. In the negative ESI mode on the other hand, our results showed the following coverage of extraction across all lipid classes: Folch > MMC > MTBE > Bligh (598, 572, 558, and 528 extracted lipids, respectively). This decreasing order of efficiency of the two-phase extraction systems is in agreement with the results previously reported by Lee et al. [22].

Comparing the two best extraction systems (MMC and Folch) in the positive ESI mode, we observed that MMC is more efficient for medium (PI, PC, PE, PG, SM, and DG) to highly apolar lipids (CE and TG), while Folch performs better for more polar lipids (LPL and MG) (Fig. 2C). This result contrasts with the “all-in-one-tube” idea of the MMC extraction, in which one would expect to see the highest number of lipids with a more polar nature. The Folch extraction method appears to be better suited for the extraction of PC, PI, PG, PS, PE, and SM when analyzed in the negative ESI mode, while MMC and Folch show the same selectivity for FA and LPL (Fig. 3C). Pellegrino et al. [3], previously introduced the MMC solvent system (MeOH/MTBE/CHCl_3_) as one of the most promising extraction methods for lipid analysis, since, in comparison with the popular two-phase systems (Folch, Bligh, and MTBE), it increased the recovery from 79 to above 95% for a set of lipid standards covering a broad polarity range. Although, in the current work, we are not only taking a small defined set of standard lipid compounds into account but the total extractable set of plasma lipids, our results agree with these findings. Moreover, the experimental simplicity of this one-phase approach makes it the preferred method for untargeted lipid analysis.

Previously, pure isopropanol (IPA) and mixtures with other solvents have been used for lipidomics analysis. Pellegrino et al. [3] tested a precipitating solvent based on a mixture of MeOH/IPA and found an average low recovery (62.8%) for a set of lipid standards. Sarafian et al. [23], on the other hand, found that it was possible to get a repeatable extraction of the lipidome from plasma samples using pure IPA as precipitating solvent with increased lipid coverage and good recovery (> 60–80%). Taking the physical-chemical characteristics of both solvents into account, we did not perform an experimental comparison of solvent systems containing IPA, since we believe their relatively high polarity might increase the amount of contaminant features and probably having a negative effect on our results.

### Relative losses of identified lipid species

**Fig. 4:**
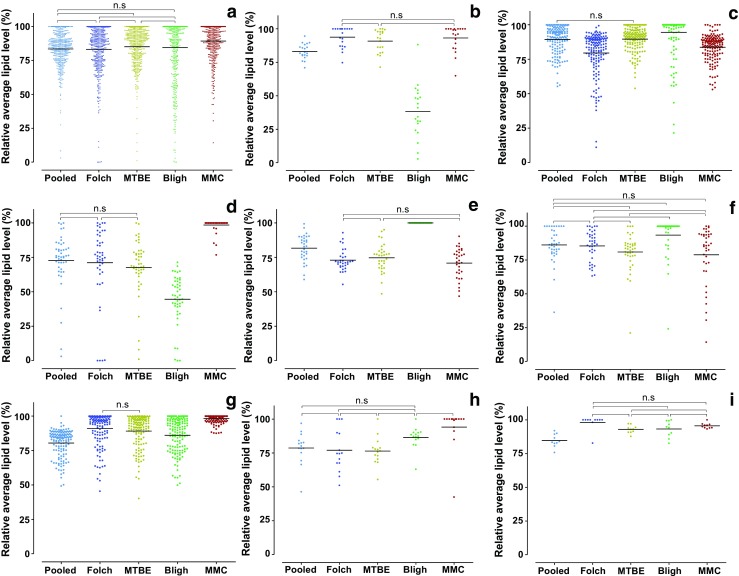
Assessment of method-induced losses of identified lipid species for the different extraction approaches. (**A**) All identified lipid species, (**B**) fatty acids, (**C**) phospholipids, (**D**) lysophospholipids, (**E**) ceramides, (**F**) sphingomyelins, (**G**) triglycerides, (**H**) diglycerides, and (**I**) cholesterol esters. For visualization purposes, levels are expressed as percentage of the highest observed average level for each identified lipid. Statistically significant differences (*p* < 0.05, Newman-Keuls multiple comparison test; performed on the absolute average levels) were found between all comparisons, unless specified otherwise in the figure

It is not feasible to evaluate the recovery of the 460 lipid species identified here, due to the lack of internal standards. Alternatively, following the work recently published by Klont et al. [24], we evaluated method-induced losses on the basis of lipids that were identified and subjected to relative quantification in all 45 measurements (four extraction methods and one pooled sample, nine replicates of each analysis). Average levels of each compound were calculated for each method based on peak intensities. Then, relative values of a particular compound for one extraction method were calculated as percentage of the value relative to the most abundant condition (extraction method) (see ESM Table S1). Overall, the Folch, MTBE, and Bligh methods showed a similar extraction performance with average lipid losses between 14.9 and 16.9% (Fig. 4a). These were in line with the losses observed in the pooled sample (16.6%). This figure furthermore shows that MMC extraction results in statistically significantly reduced lipid losses of 10.7%. Repeatability of the MMC extraction method and the pooled sample were similar with coefficients of variation of 14.6 and 14.9%, respectively, while the two-phase lipid extraction systems had coefficients of variation between 16.3 and 26.2%, with the Bligh extraction method being the most variable. The MMC method yielded the highest levels of FA, LPL, TG, and DG (Fig. 4b–h), while the Bligh extraction method gave the highest levels for phospholipids (PLs) in general as well as for SM. However, it resulted in the greatest overall losses of all extraction methods, particularly affecting FA and LPL (Fig. 4).

### Polar lipids species are lost in the hydrophilic fraction of two-phase extractions systems

**Fig. 5:**
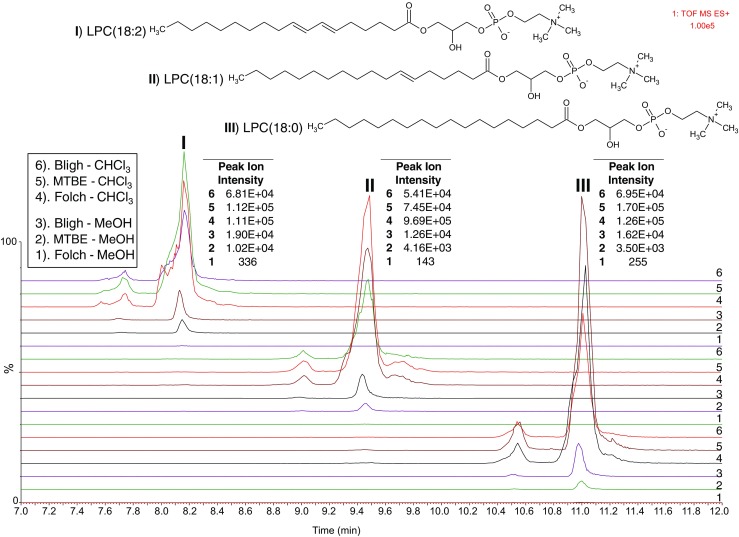
Comparison of the relative abundance of a representative set of lysophophatidylcholines in positive ESI mode present in the chloroform- and the methanol-rich aqueous phases of the three tested two-phase extraction methods

**Fig. 6:**
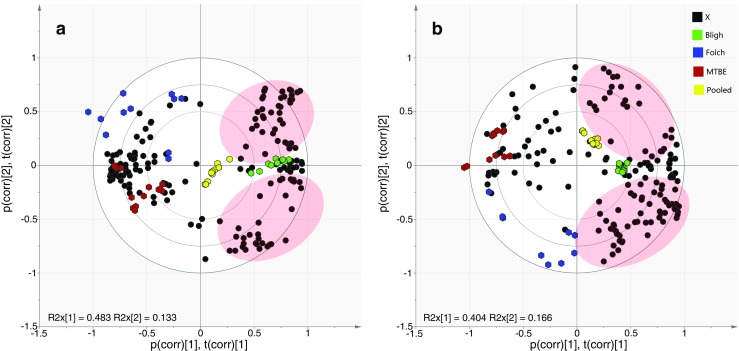
PCA-Biplot (score and loading plots are overlaid) comparing the hydrophilic fractions of the two-phase extraction systems (Folch, Bligh, and MTBE) against a hydrophilic pooled sample in positive (**A**) and negative (**B**) ESI mode. The features taken into account for this analysis are represented as “X”

To gain a better understanding of the reason behind the differences observed in segment I of the chromatograms with the different two-phase lipid extraction systems, we analyzed the content of the remnant hydrophilic phases that are usually discarded. We followed a set of endogenous lysophophatidylcholines (LPC) with a C18 carbon chain and up to two double bonds in the positive ESI mode (Fig. 5) and a set of endogenous FA with the same number of carbon atoms and up to three double bonds in the negative ESI mode (ESM Fig. S4). According to our results, the Folch extraction method shows only minor signals of LPC and FA in the more hydrophilic fraction, while the MTBE and notably the Bligh method showed significant levels of LPC- and FA-derived signals in the positive and negative ESI modes, respectively. Figure 6 shows a PCA-Biplot (score and loading plots are overlaid) of the extracted features in the methanol phase of all two-phase extractions systems. This plot displays similarities and dissimilarities between observations and allows us to interpret the observations in terms of the variables/features. Observations close to the origins do not contribute to the formation of the clusters and are poorly described by the model components. As highlighted in Fig. 6 by the ellipses, most of the extracted lipid features present in the hydrophilic phases are related to the Bligh and Dyer extraction system. This explains the low total coverage of lipids with this extraction method observed in the Venn diagrams for the hydrophobic fraction (Figs. 2 and 3), indicating that the Bligh and Dyer method is less well suited for untargeted lipidomics.

## Conclusion

By comparing a pooled extract with the extracts of four different sample preparation methods for lipidomics, we tried to establish which of the methods is most comprehensive (closest to the pooled extract in terms of lipid composition) and which of the methods show significant differences. While a pooled extract might be considered most comprehensive, it is not practical to perform two or more extractions with different methods in order to increase the coverage of the number of extracted lipids. Instead, a straightforward procedure able to perform this task in a simple way is much preferred. In this regard, one-phase extraction methods, and specifically in our case, the MMC method (MeOH/MTBE/CHCl_3_) developed by Pellegrino et al. [3] showed the best results as it turned out to be quantitatively and qualitatively most similar to the pooled extract.

The most important differences were observed for the Bligh and Dyer extraction. Particularly, more polar lipid species like LPC or FA were lost in the methanol-rich hydrophilic phase of this extraction approach, which is usually discarded for lipid analysis.

## Electronic supplementary material

ESM 1 (PDF 2.89 MB)
