# Supplementary material for: One- vs two-phase extraction: re-evaluation of sample preparation procedures for untargeted lipidomics in plasma samples
Source: Anal Bioanal Chem. 2018 Jul 2;410(23):5859–70. doi: 10.1007/s00216-018-1200-x (PMC6096717; doi:10.1007/s00216-018-1200-x)
Supplement: Supplementary file 1 — (PDF 2.89 MB) [file 216_2018_1200_MOESM1_ESM.pdf]

Analytical and Bioanalytical Chemistry

Electronic Supplementary Material

One- vs two-phase extraction: re-evaluation of sample preparation procedures for untargeted lipidomics in plasma samples

Andres Gil, Wenxuan Zhang, Justina C. Wolters, Hjalmar Permentier, Theo Boer,

Peter Horvatovich, Rebecca Heiner-Fokkema,Dirk-Jan Reijngoud, Rainer Bischoff

CONTENT

Supporting materials and methods

Fig. S1 PCA line plot of the QC

Fig. S2 Supervised multivariate analysis in positive ESI mode

Fig. S3 Supervised multivariate analysis in negative ESI mode

Fig. S4 Comparison of the relative abundance for a set of FA in the chloroform- and methanol-rich aqueous phases

Figs. S5 to S8 Comparison of plasma and blank samples extracted with the Folch, MTBE, Bligh and MMC extraction methods, respectively

Table S1 List of identified lipids species and relative quantitation

Supporting materials and methods

Chemicals

All chemicals used were analytical grade or of the highest purity commercially available. Methanol (MeOH), acetonitrile (ACN), isopropanol (IPA) and chloroform ($CHCl_3$) were purchased from BIOSOLVE (Valkenswaard, Netherlands). Methyl tert-butyl ether (MTBE) and ammonium formate (for mass spectrometry) were purchased from Sigma-Aldrich (Zwijndrecht, The Netherlands). Ultrapure water was obtained from a Milli-Q Advantage A10 water purification system at a resistivity of 18.2 MΩ cm (Millipore SAS, Molsheim, France). Plastic tubes that do not get altered by the use of organic solvents were purchased from Eppendorf (Order no. 0030 120.094).

Blood collection

Heparin-anticoagulated plasma samples, obtained from adult patients at the University Medical Center Groningen (UMCG) in an anonymous manner, were combined to generate a standard plasma sample. This sample was separated in aliquots that were stored at -80°C in the dark (up to 1 year) until further use. Respective triglycerides and cholesterol concentrations in the combined plasma sample were 1.66 and 3.6 mM. The study design was in accordance with the current revision of the Helsinki Declaration (2013).

Data preprocessing

MassLynx software version 4.1 was used for data acquisition. Waters raw data files were analyzed using Progenesis QI software (Waters Corporation, Milford, MA) for peak alignment, peak picking and normalization of the LC-MS data. Peak alignment was done to correct drifts in retention times. To this end a reference LC-MS run, that was the best representative of the entire data set, was selected. All other runs were then aligned to this reference. For peak picking and feature selection the following adduct forms were used: [M+H], [$M+NH_4$], [M+Na], [M+K], [$M+H-H_2O$], [$M+CH_3OH+H$], [2M+H], [$2M+NH_4$] and [2M+Na] in positive mode; and [M-H], [M+FA-H], [M+Cl], [2M+FA-H] and [$M-H_2O-H$] in negative mode. The peak picking limits were set at the maximum sensitivity mode. To this end the software uses an algorithm to automatically determine the noise level in the data. This automatic method examines the intensities of groups of MS ions to judge whether they are likely to form part of a peak or whether they represent noise and thus should be ignored. A default automatic normalization approach called "normalize to all compounds" was used. This normalization automatically selects a reference LC-MS measurement and then uses ratiometric data in a log space, along with a median and mean absolute deviation outlier filtering approach, to compare and calculate a scalar factor for the remaining measurements. The metabolite features reported hereafter were entirely generated using the Progenesis software.

On the basis of normalized peak intensities, the number of features was filtered according to 2 different sets of selection criteria. The first set of selection was strict and only applied for PCA. This included observations showing a change in magnitude (Max fold change $\geq$ 1.5), a

statistically significant difference ($P \leq 0.05$, student's t-test), while excluding features with a variation of more than 30% (CV) within each experimental group (i.e. pooled, Folch, Bligh, MTBE and MMC samples). The second set of selection, only applied for OPLS-DA, was less strict and included all observations showing a CV ≤ 30% within each experimental group. These set of selections allowed to avoid over-fitting and improved the multivariate model's predictive ability[17]. Final results were represented in an output table containing m/z values, retention times and normalized peak intensities for each compound ion (feature) in the two pooled extracts and the samples obtained with the individual extraction systems. This table was imported into Simca P v.13 (Umetrics, Umea, Sweden) for multivariate statistical analysis.

Multivariate statistical analysis

Principal component analysis (PCA) and partial least squares discriminant analysis (OPLS-DA) via orthogonal projection to latent structures were carried out on the filtered features using Simca P v.13 (Umetrics, Umea, Sweden). Data were $\log_{10}$ transformed, mean-centered, pareto-scaled, and columns representing samples were grouped in blocks according to the extraction methods (Folch, Bligh, MTBE and MMC), as well as to the pooled extracts (hydrophobic and hydrophilic). Discriminating features between lipid profiles were identified for each model using s-plots [17]. Permutation testing (n = 999) was carried out to determine the robustness of the multivariate models (i.e. whether a model fits the training set well and accurately predicts the variable under study (Y) for new observations).

The latter was made possible by the sevenfold cross validation used as default by the Simca software. In this procedure $1/7^{th}$ of the data is omitted from model building and the obtain model is used to predict the omitted data class membership. In this cross validation, determination coefficient (R^2) represents the fraction of the data variation explained by the multivariate regression model. A large R^2 (close to 1) is directly related to a good reproducibility or low noise in the dataset. Q^2 represents an estimate of the predictive ability of the model. A large Q^2 (> 0.5) indicates good prediction performance.

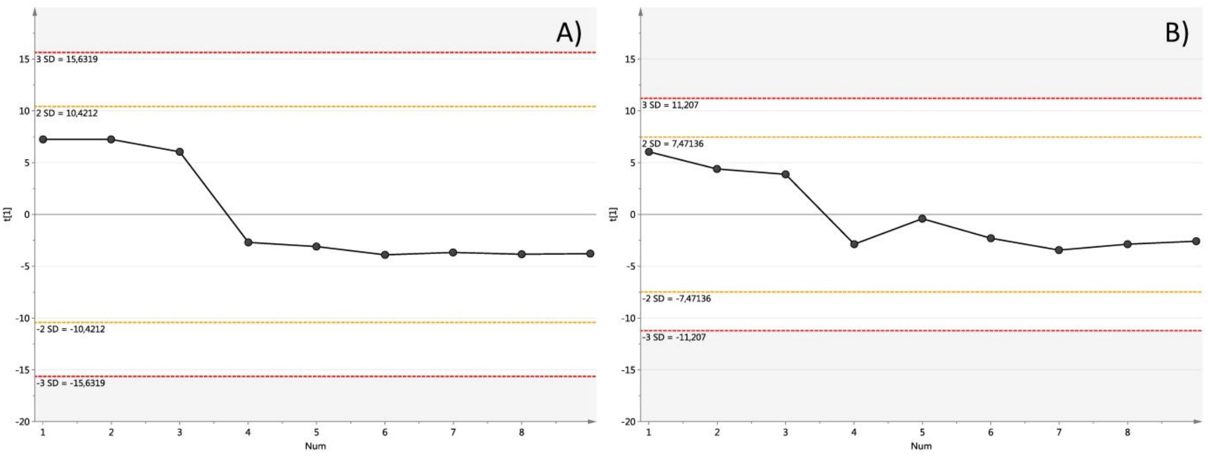

Fig. S1 PCA line plot of the Pooled sample (QC) using the first component in positive (A) and negative (B) ionization mode. The plots represent the technical variation observed for the LC-MS data across the experiments

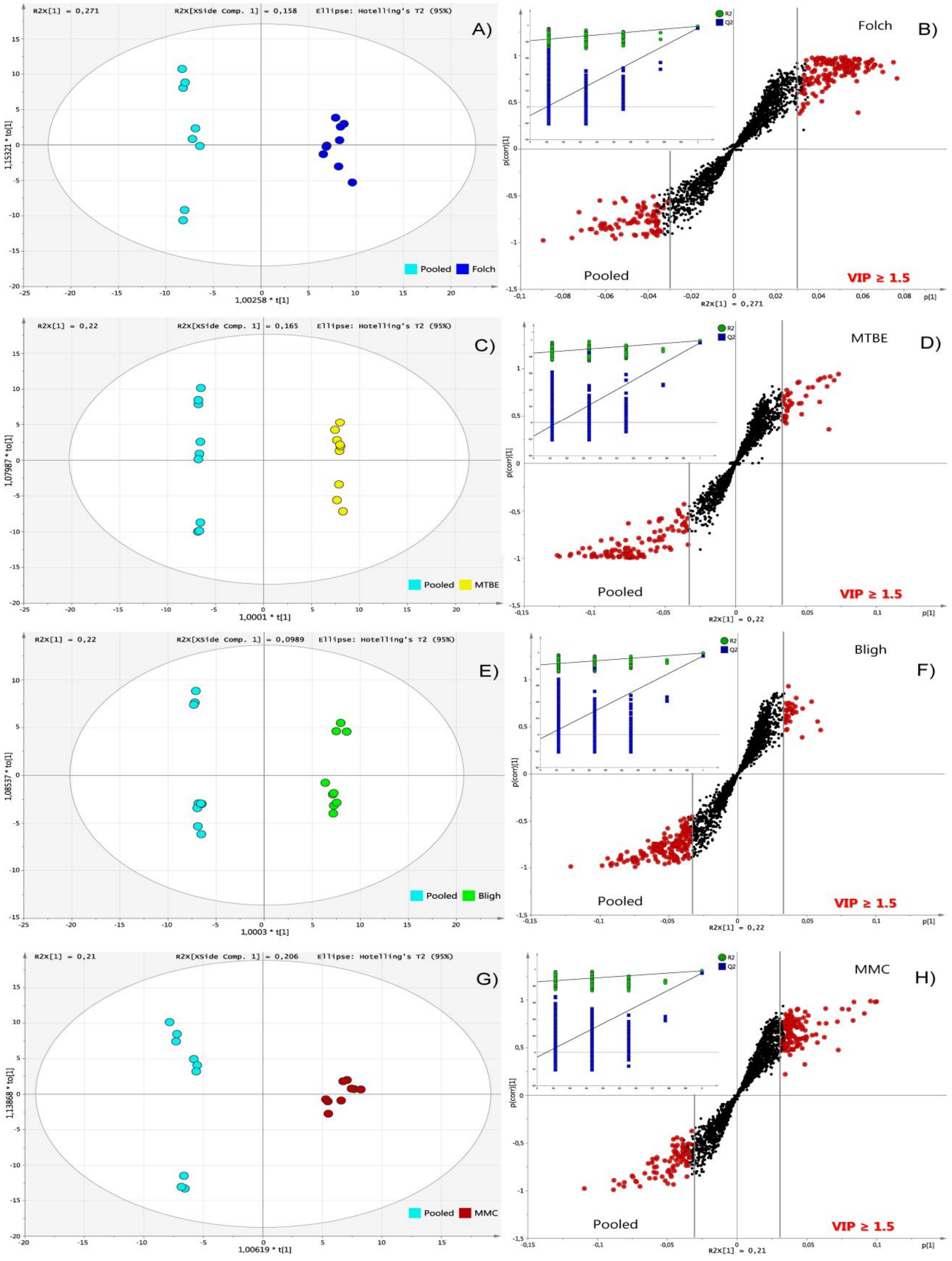

Fig. S2 OPLS-DA scores plots and S-Plots showing the separation and discriminating features (respectively) between the pooled sample (QCs) and the Folch (A, B), MTBE (C, D), Bligh (E, F) and MMC (G, H) extracts in positive mode. Validation plots displaying 999 permutation tests for the models are shown within the S-Plots. The explained variances (R2) were 0.990, 0.993, 0.995 and 0.983, and predictive abilities (Q2) were 0.965, 0.968, 0.960 and 0.954 for the "Pooled vs Folch", "Pooled vs MTBE", "Pooled vs Bligh" and "Pooled vs MMC" models respectively

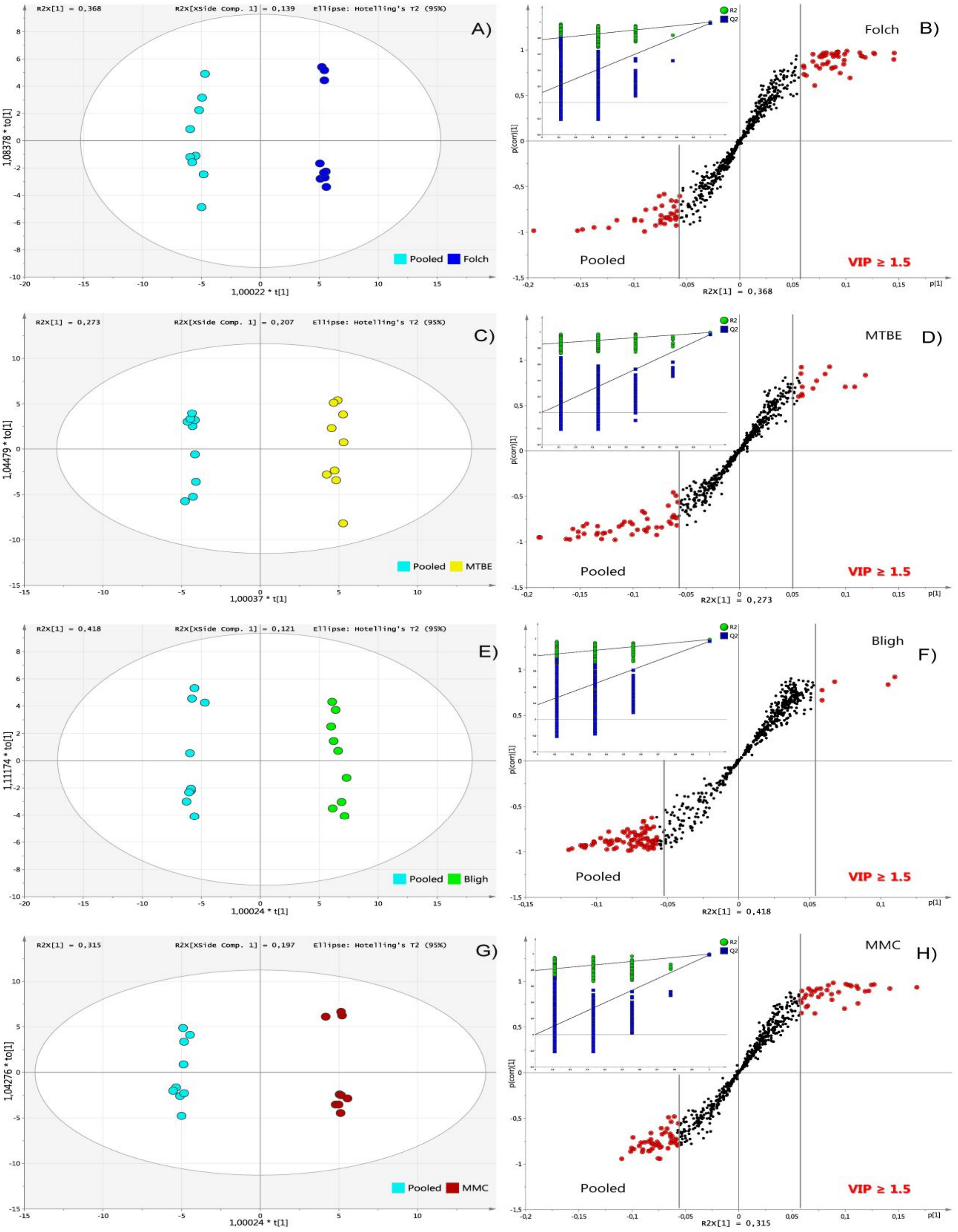

Fig. S3 OPLS-DA scores plots and S-Plots showing the separation and discriminating features (respectively) between the pooled sample (QCs) and the Folch (A, B), MTBE (C, D), Bligh (E, F) and MMC (G, H) extracts in negative mode. Validation plots displaying 999 permutation tests for the models are shown within the S-Plots. The explained variances (R2) were 0.996, 0.994, 0.990 and 0.996, and predictive abilities (Q2) were 0.983, 0.969, 0.968 and 0.987 for the “Pooled vs Folch”, “Pooled vs MTBE”, “Pooled vs Bligh” and “Pooled vs MMC” models respectively

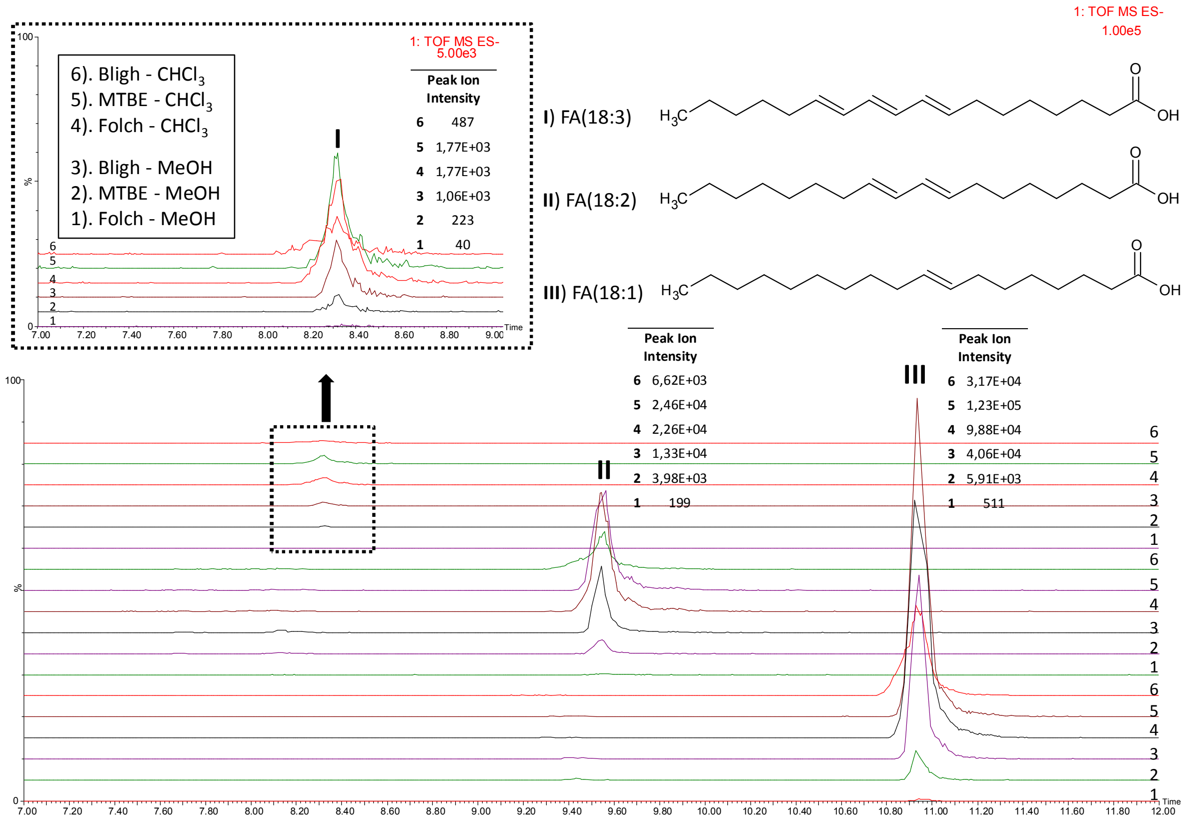

Fig. S4 Comparison of the relative abundance of a representative set of fatty acids in negative ESI mode present in the chloroform- and the methanol-rich aqueous phases of the 3 tested two-phase extraction methods

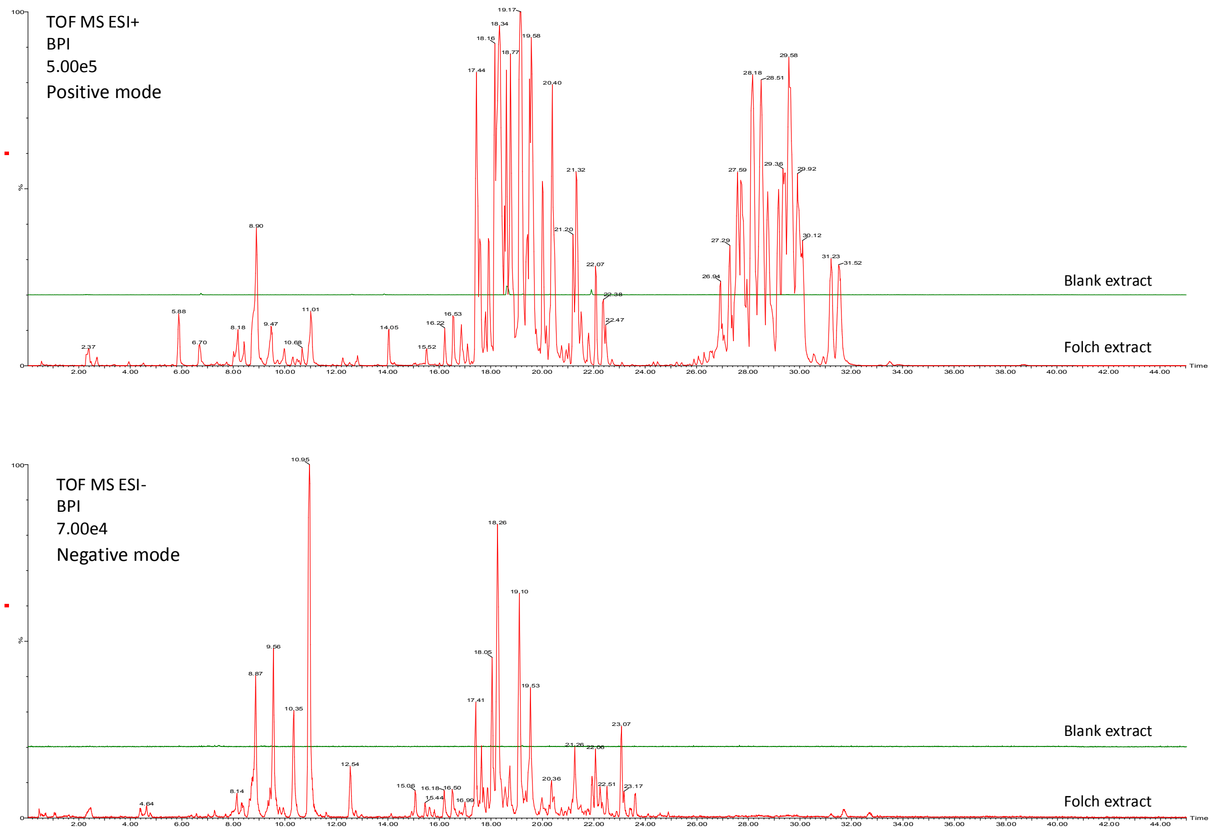

Fig. S5 Comparison of plasma and blank samples extracted with Folch method in positive and negative mode

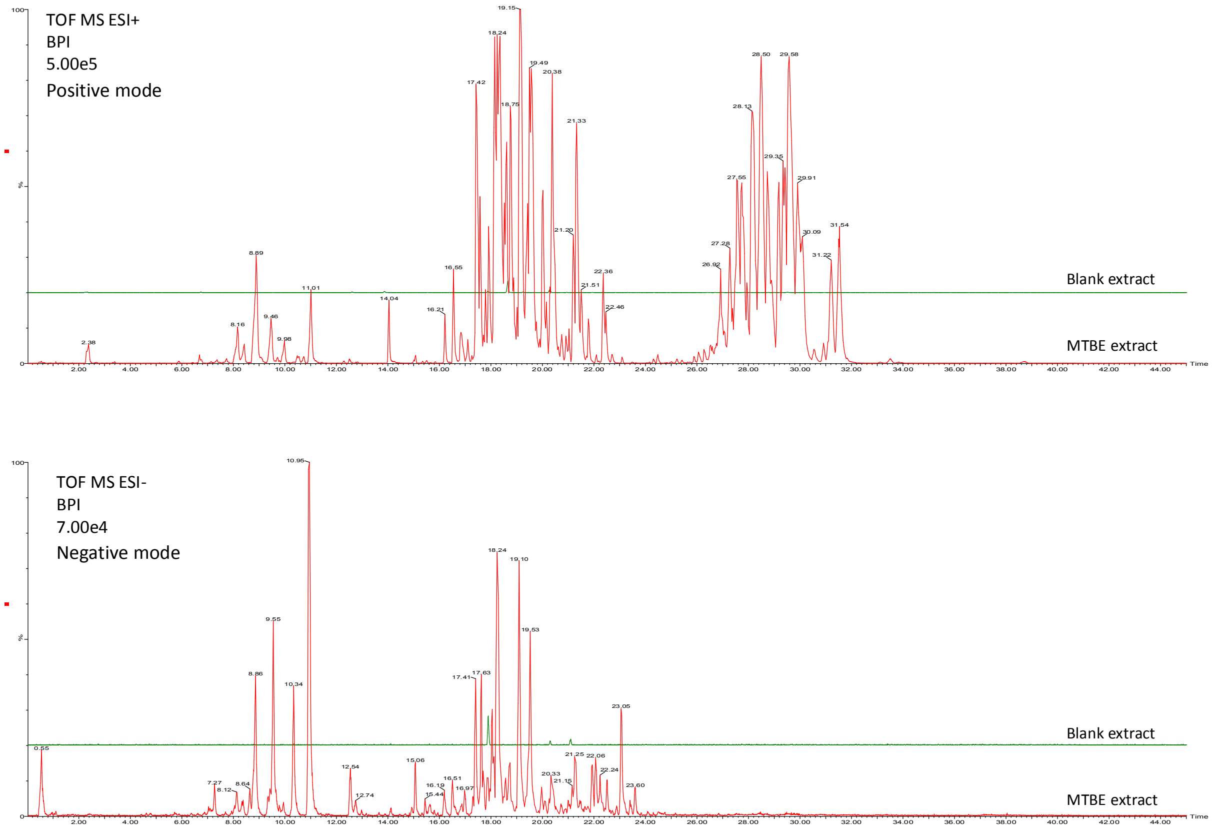

Fig. S6 Comparison of plasma and blank samples extracted with MTBE method in positive and negative mode

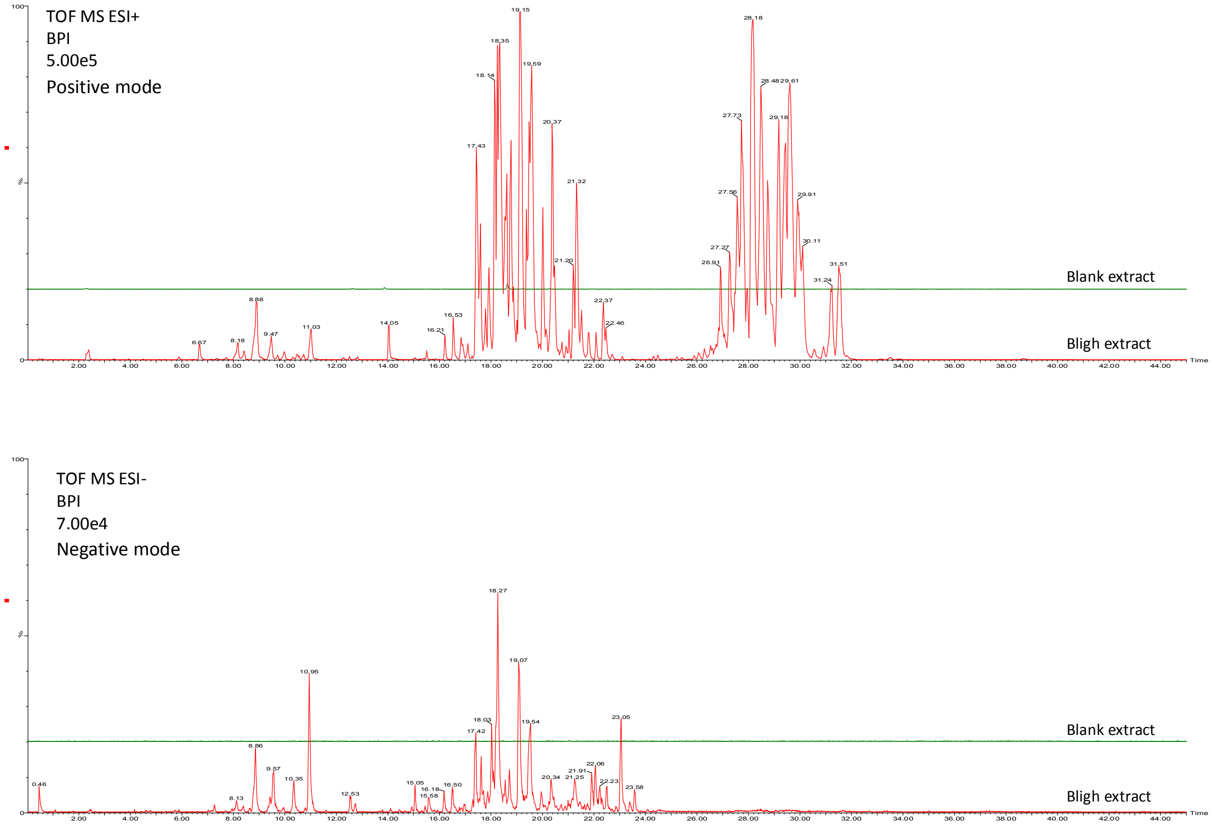

Fig. S7 Comparison of plasma and blank samples extracted with Bligh method in positive and negative mode

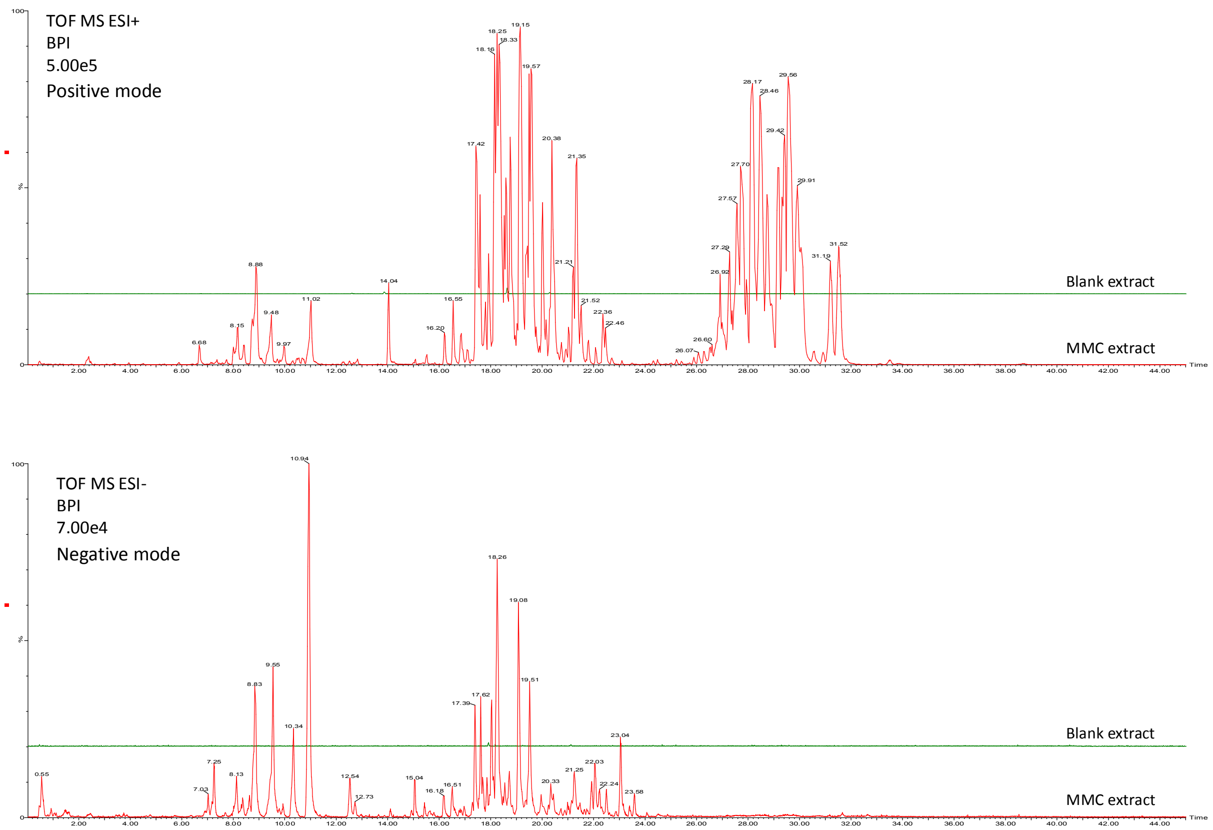

Fig. S8 Comparison of plasma and blank samples extracted with MMC method in positive and negative mode

Table S1 List of identified lipids species and relative quantitation

Compound	ccepted Compound	Accepted Description	m/z	Charge	Retention	Chromato	Anova (p)	Max Fold	Highest M	Lowest M	IS	Accepted	Accepted Description	Adducts	Formula	Score	Average intensity					MAX intensity	Relative Pecentage				
																	POOL Av	Folch ave	MTBE ave	Bligh ave	MMC average		POOL	Folch	MTBE	Bligh	MMC
7.96_227.2008m/z	FA01010001	FA(14:0)	227,2008	1	7,961517	0,301833	0	6,814262	MMC	Bligh		FA010100	FA(14:0)	M-H	C14H28O	51,6	24,64437	26,45729	25,05571	4,485165	30,56309	30,56309	80,63%	86,57%	81,98%	14,68%	100,00%
10.37_255.2324m/z	FA01010004	FA(16:0)	255,2324	1	10,3656	0,5445	0	2,073971	MMC	Bligh		FA010100	FA(16:0)	M-H	C16H32O	60,4	559,8292	638,8254	656,6563	330,4186	685,2785	685,2785	81,69%	93,22%	95,82%	48,22%	100,00%
11.52_269.2476m/z	FA01010006	FA(17:0)	269,2476	1	11,52137	0,169233	0	35,23167	Folch	Bligh		FA010100	FA(17:0)	M-H	C17H34O	49,9	5,941618	7,201287	5,139011	0,204398	4,675672	7,201287	82,51%	100,00%	71,36%	2,84%	64,93%
12.58_283.2633m/z	FA01010007	FA(18:0)	283,2633	1	12,57598	0,429833	0	2,098369	Folch	Bligh		FA010100	FA(18:0)	M-H	C18H36O	57	340,2974	433,615	349,7437	206,6438	338,6184	433,615	78,48%	100,00%	80,66%	47,66%	78,09%
6.40_225.1851m/z	FA01010008	FA(14:1)	225,1851	1	6,398817	0,198533	0	13,89469	MMC	Bligh		FA010100	FA(14:1)	M-H	C14H26O	45,8	13,72216	13,50462	14,46037	1,301748	18,08738	18,08738	75,87%	74,66%	79,95%	7,20%	100,00%
8.66_253.2165m/z	FA01010009	FA(16:1)	253,2165	1	8,65645	0,459583	0	3,417723	MMC	Bligh		FA010100	FA(16:1)	M-H	C16H30O	52,7	169,0299	183,9889	182,5169	61,82228	211,2915	211,2915	80,00%	87,08%	86,38%	29,26%	100,00%
9.81_267.2321m/z	FA01010010	FA(17:1)	267,2321	1	9,811083	0,37815	0	3,053277	MMC	Bligh		FA010100	FA(17:1)	M-H	C17H32O	49,6	97,11222	92,57408	106,8822	35,73961	109,1229	109,1229	88,99%	84,83%	97,95%	32,75%	100,00%
13.03_309.2789m/z	FA01010013	FA(20:1)	309,2789	1	13,029	0,22005	1,11E-16	2,918778	MTBE	Bligh		FA010100	FA(20:1)	M-H	C20H38O	51,3	31,9076	32,15584	36,94861	12,65893	31,61925	36,94861	86,36%	87,03%	100,00%	34,26%	85,58%
16.19_365.3417m/z	FA01010015	FA(24:1)	365,3417	1	16,18768	0,154067	0,000115	1,726577	Folch	Bligh		FA010100	FA(24:1)	M-H	C24H46O	52,4	8,77266	9,2799	9,081642	5,374737	7,906609	9,2799	94,53%	100,00%	97,86%	57,92%	85,20%
8.15_279.2319m/z	FA01010016	FA(18:2)	279,2319	1	8,145233	0,229067	0,738156	1,166325	Folch	Pooled		FA010100	FA(18:2)	M-H	C18H32O	49,4	7,852243	9,158268	7,897394	8,076664	8,452312	9,158268	85,74%	100,00%	86,23%	88,19%	92,29%
9.57_279.2322m/z	FA01010017	FA(18:2)	279,2322	1	9,574017	0,75515	1,11E-16	2,047665	MTBE	Bligh		FA010100	FA(18:2)	M-H	C18H32O	56,8	877,215	905,3646	978,2215	477,7253	939,8243	978,2215	89,67%	92,55%	100,00%	48,84%	96,07%
11.74_307.2632m/z	FA01010018	FA(20:2)	307,2632	1	11,73782	0,175217	0	4,452746	Folch	Bligh		FA010100	FA(20:2)	M-H	C20H36O	49,9	10,3763	13,72959	13,29653	3,083399	12,24101	13,72959	75,58%	100,00%	96,85%	22,46%	89,16%
8.36_277.2163m/z	FA01010019	FA(18:3)	277,2163	1	8,3595	0,46225	0	3,228976	Folch	Bligh		FA010100	FA(18:3)	M-H	C18H30O	48,7	97,55553	121,9638	113,1922	37,77167	120,9051	121,9638	79,99%	100,00%	92,81%	30,97%	99,13%
10.51_305.2477m/z	FA01010020	FA(20:3)	305,2477	1	10,51163	0,302417	0	3,212361	MMC	Bligh		FA010100	FA(20:3)	M-H	C20H34O	56,5	41,71674	47,49931	47,86616	15,25581	49,00717	49,00717	85,12%	96,92%	97,67%	31,13%	100,00%
9.54_303.2320m/z	FA01010023	FA(20:4)	303,232	1	9,5396	0,554767	3,41E-10	1,800819	MMC	Bligh		FA010100	FA(20:4)	M-H	C20H32O	51,6	96,5621	106,7181	110,1722	65,56264	118,0664	118,0664	81,79%	90,39%	93,31%	55,53%	100,00%
11.41_331.2634m/z	FA01010024	FA(22:4)	331,2634	1	11,40993	0,17105	7,31E-12	2,264842	Folch	Bligh		FA010100	FA(22:4)	M-H	C22H36O	56,4	18,67306	21,67416	21,48161	9,569834	20,84977	21,67416	86,15%	100,00%	99,11%	44,15%	96,20%
8.35_301.2163m/z	FA01010025	FA(20:5)	301,2163	1	8,351933	0,27855	0	4,141364	Folch	Bligh		FA010100	FA(20:5)	M-H	C20H30O	48,8	18,09324	25,49399	20,98637	6,155942	24,32012	25,49399	70,97%	100,00%	82,32%	24,15%	95,40%
10.29_329.2477m/z	FA01010026	FA(22:5)	329,2477	1	10,2853	0,284283	0	2,436834	Folch	Bligh		FA010100	FA(22:5)	M-H	C22H34O	55,5	54,93652	62,24246	53,6545	25,54235	51,40128	62,24246	88,26%	100,00%	86,20%	41,04%	82,58%
9.37_327.2321m/z	FA01010027	FA(22:6)	327,2321	1	9,373033	0,448517	1,18E-11	1,849702	MMC	Bligh		FA010100	FA(22:6)	M-H	C22H32O	56,9	104,5714	107,1112	118,0413	70,2625	129,9647	129,9647	80,46%	82,42%	90,83%	54,06%	100,00%
15.48_351.3260m/z	FA01010028	FA(23:1)	351,326	1	15,47507	0,312167	5,58E-13	1,930658	Folch	Bligh	x	FA010100	FA(23:1)	M-H	C23H44O	56,7	112,8702	125,9069	125,2304	65,21452	124,6481	125,9069	89,65%	100,00%	99,46%	51,80%	99,00%
17.70_662.4759m/z	GPEtn(15:0/15:0)	PE(30:0)	662,4759	1	17,70167	0,377633	0,000914	1,349292	Pooled	Folch	x	GPEtn(15:	PE(30:0)	M-H	C35H70N	51	134,9304	100,0009	108,3421	120,8636	112,8941	134,9304	100,00%	74,11%	80,29%	89,57%	83,67%
20.18_718.5380m/z	GP02010004	PE(34:0)	718,538	1	20,18412	0,403617	0,00393	1,42135	Bligh	MMC		GP02010	PE(34:0)	M-H	C39H78N	73,2	158,043	135,463	147,5883	165,6832	116,5675	165,6832	95,39%	81,76%	89,08%	100,00%	70,36%
21.38_746.5692m/z	GPEtn(18:0/18:0)	PE(36:0)	746,5692	1	21,37598	0,435067	1,10E-06	1,59574	Bligh	MMC		GPEtn(18:	PE(36:0)	M-H	C41H82N	53,4	198,7781	152,3885	145,095	211,9131	132,7993	211,9131	93,80%	71,91%	68,47%	100,00%	62,67%
17.93_716.5227m/z	GP02010006	PE(34:1)	716,5227	1	17,92808	0,234067	0,08166	1,538631	Bligh	MTBE		GP02010	PE(34:1)	M-H	C39H76N	62,7	25,84487	24,89036	20,66831	31,80092	24,95147	31,80092	81,27%	78,27%	64,99%	100,00%	78,46%
19.22_716.5227m/z	GP02010007	PE(34:1)	716,5227	1	19,22487	0,360133	0,016989	1,288951	Bligh	MMC		GP02010	PE(34:1)	M-H	C39H76N	69,6	116,6955	109,1242	115,7687	126,1707	97,88633	126,1707	92,49%	86,49%	91,76%	100,00%	77,58%
20.42_744.5537m/z	GP02010010	PE(36:1)	744,5537	1	20,42473	0,257033	0,002387	1,432256	Bligh	MMC		GP02010	PE(36:1)	M-H	C41H80N	70,7	39,53654	38,28321	36,8824	48,94472	34,17317	48,94472	80,78%	78,22%	75,36%	100,00%	69,82%
20.40_772.5849m/z	GP02010011	PE(38:1)	772,5849	1	20,39912	0,3217	0,001652	1,441193	Bligh	Pooled		GP02010	PE(38:1)	M-H	C43H84N	67	29,00084	32,00698	31,66774	41,79579	32,53409	41,79579	69,39%	76,58%	75,77%	100,00%	77,84%
18.40_714.5071m/z	GP02010015	PE(34:2)	714,5071	1	18,40292	0,424583	0,007033	1,257049	Bligh	Pooled		GP02010	PE(34:2)	M-H	C39H74N	62,1	105,5653	113,2656	119,0307	132,7007	123,2025	132,7007	79,55%	85,35%	89,70%	100,00%	92,84%
19.65_742.5380m/z	GP02010019	PE(36:2)	742,538	1	19,64635	0,493833	3,06E-09	1,569857	Bligh	Folch		GP02010	PE(36:2)	M-H	C41H78N	55,3	285,6886	220,1764	310,7625	345,6453	280,2444	345,6453	82,65%	63,70%	89,91%	100,00%	81,08%
19.57_770.5690m/z	GP02010020	PE(38:2)	770,569	1	19,57448	0,4936	5,42E-07	1,382463	Bligh	Folch		GP02010	PE(38:2)	M-H	C43H82N	63,1	111,3878	100,0382	115,4975	138,2991	120,2853	138,2991	80,54%	72,33%	83,51%	100,00%	86,97%
18.66_740.5224m/z	GP02010022	PE(36:3)	740,5224	1	18,66363	0,548683	0,000422	1,421103	Bligh	Pooled		GP02010	PE(36:3)	M-H	C41H76N	51,9	89,85394	96,53457	106,3866	127,6917	91,01496	127,6917	70,37%	75,60%	83,32%	100,00%	71,28%
20.06_768.5534m/z	GP02010024	PE(38:3)	768,5534	1	20,0554	0,238417	0,01091	1,402673	Bligh	MMC		GP02010	PE(38:3)	M-H	C43H80N	52,1	27,20511	27,96232	28,02524	33,20998	23,67621	33,20998	81,92%	84,20%	84,39%	100,00%	71,29%
18.35_738.5069m/z	GP02010026	PE(36:4)	738,5069	1	18,34553	0,372283	2,11E-07	1,233607	Bligh	Pooled		GP02010	PE(36:4)	M-H	C41H74N	57,5	137,2072	140,7538	151,6623	169,2598	141,0362	169,2598	81,06%	83,16%	89,60%	100,00%	83,33%
19.57_766.5381m/z	GP02010031	PE(38:4)	766,5381	1	19,57448	0,357283	8,78E-11	1,411863	Bligh	Folch		GP02010	PE(38:4)	M-H	C43H78N	63	332,261	275,5224	337,2402	389	308,3246	389,0000	85,41%	70,83%	86,69%	100,00%	79,26%
19.51_794.5688m/z	GP02010032	PE(40:4)	794,5688	1	19,50548	0,262117	1,22E-06	1,427339	Bligh	Folch		GP02010	PE(40:4)	M-H	C45H82N	60	26,16538	24,60104	29,86338	35,11403	28,39804	35,11403	74,52%	70,06%	85,05%	100,00%	80,87%
18.61_764.5222m/z	GP02010034	PE(38:5)	764,5222	1	18,60635	0,377783	0,007452	1,35707	Bligh	Pooled		GP02010	PE(38:5)	M-H	C43H76N	62,8	120,1539	124,4255	130,8343	163,0573	129,1534	163,0573	73,69%	76,31%	80,24%	100,00%	79,21%
18.91_764.5223m/z	GP02010035	PE(38:5)	764,5223	1	18,9093	0,322533	1,60E-06	1,643539	Bligh	Folch		GP02010	PE(38:5)	M-H	C43H76N	54,3	34,60355	26,57792	32,18768	43,68183	27,23093	43,68183	79,22%	60,84%	73,69%	100,00%	62,34%
19.81_792.5538m/z	GP02010036	PE(40:5)	792,5538	1	19,81297	0,21515	0,000388	1,618588	Bligh	MMC		GP02010	PE(40:5)	M-H	C45H80N	54,7	17,03564	15,5967	14,12625	19,15302	11,83317	19,15302	88,94%	81,43%	73,75%	100,00%	61,78%
18.24_762.5069m/z	GP02010038	PE(38:6)	762,5069	1	18,243	0,37555	6,60E-06	1,353861	Bligh	Pooled		GP02010	PE(38:6)	M-H	C43H74N	60,9	188,0341	197,9837	200,1024	254,5721	196,2257	254,5721	73,86%	77,77%	78,60%	100,00%	77,08%
19.47_790.5380m/z	GP02010040	PE(40:6)	790,538	1	19,4709	0,346117	0,004431	1,368194	Bligh	Folch		GP02010	PE(40:6)	M-H	C45H78N	56,9	124,036	118,2659	149,6476	161,8107	154,0935	161,8107	76,65%	73,09%	92,48%	100,00%	95,23%
18.48_788.5225m/z	GP02010041	PE(40:7)	788,5225	1	18,48167	0,321717	3,91E-05	1,434807	Bligh	MMC		GP02010	PE(40:7)	M-H	C45H76N	54,6	34,77278	33,40344	32,24362	45,33076	31,59362	45,33076	76,71%	73,69%	71,13%	100,00%	69,70%
21.01_728.5587m/z	GP02020003	PE(O-36:2)//PE(P-36:1)	728,5587	1	21,01305	0,179367	0,153436	1,281128	MTBE	Folch		GP02020	PE(O-36:2)//PE(P-36:1)	M-H	C41H80N	63,6	11,95753	11,1381	14,26933	13,55842	13,13343	14,26933	83,80%	78,06%	100,00%	95,02%	92,04%
18.98_698.5120m/z	GP02020004	PE(O-34:3)//PE(P-34:2)	698,512	1	18,98173	0,323167	0,002722	1,332664	Bligh	MMC		GP02020	PE(O-34:3)//PE(P-34:2)	M-H	C39H74N	54,1	27,18548	21,65637	25,81353	28,02609	21,03012	28,02609	97,00%	77,27%	92,11%	100,00%	75,04%
20.23_726.5430m/z	GP02020005	PE(O-36:3)//PE(P-36:2)	726,543	1	20,22962	0,305783	0,009982	1,313651	MTBE	Pooled		GP02020	PE(O-36:3)//PE(P-36:2)	M-H	C41H78N	59,7	39,63869	45,38907	52,07139	50,47417	44,26379	52,07139	76,12%	87,17%	100,00%	96,93%	85,01%
20.05_726.5432m/z	GP02020005	PE(O-36:3)//PE(P-36:2)	726,5432	1	20,04782	0,163033	0,035951	1,464654	Bligh	MMC		GP02020	PE(O-36:3)//PE(P-36:2)	M-H	C41H78N	51,5	9,231315	7,577091	9,028417	10,13422	6,919192	10,13422	91,09%	74,77%	89,09%	100,00%	68,28%
19.24_724.5275m/z	GP02020006	PE(O-36:4)//PE(P-36:3)	724,5275	1	19,24003	0,5364	0,039167	1,328632	Bligh	MMC		GP02020	PE(O-36:4)//PE(P-36:3)	M-H	C41H76N	56,3	48,82376	43,13882	49,58367	54,49562	41,01634	54,49562	89,59%	79,16%	90,99%	100,00%	75,27%
18.91_722.5121m/z	GP02020007	PE(O-36:5)//PE(P-36:4)	722,5121	1	18,9093	0,463067	0,000132	1,521194	Bligh	Folch		GP02020	PE(O-36:5)//PE(P-36:4)	M-H	C41H74N	63,7	117,7085	96,89622	105,6014	147,398	102,2182	147,398	79,86%	65,74%	71,64%	100,00%	69,35%
20.38_776.5585m/z	GP02020009	PE(O-40:6)//PE(P-40:5)	776,5585	1	20,38398	0,2832	4,60E-05	1,763869	Bligh	MMC		GP02020	PE(O-40:6)//PE(P-40:5)	M-H	C45H80N	55,2	18,24908	16,59218	19,31113	25,12632	14,245	25,12632	72,63%	66,04%	76,86%	100,00%	56,69%
20.12_750.5428m/z	GP02030007	PE(P-38:4)//PE(O-38:5)	750,5428	1	20,11997	0,396367	0,000133	1,375415	Bligh	MMC		GP02030	PE(P-38:4)//PE(O-38:5)	M-H	C43H78N	66	129,8855	142,0157	153,6337	170,5489	123,9981	170,5489	76,16%	83,27%	90,08%	100,00%	72,71%
21.37_778.5745m/z	GP02030009	PE(P-40:4)//PE(O-40:5)	778,5745	1	21,36833	0,201883	0,006854	1,888272	Bligh	MMC		GP02030	PE(P-40:4)//PE(O-40:5)	M-H	C45H82N	47,1	4,216186	4,142429	5,435538	6,511039	3,448148	6,511039	64,75%	63,62%	83,48%	100,00%	52,96%
18.22_720.4965m/z	PE(P-16:0/20:5)	PE(P-36:5)//PE(O-36:6)	720,4965	1	18,21607	0,29675	0,171518	1,289534	Bligh	MMC		PE(P-16:0	PE(P-36:5)//PE(O-36:6)	M-H	C41H72N	48,1	6,453921	6,059791	6,546595	7,526804	5,836839	7,526804	85,75%	80,51%	86,98%	100,00%	77,55%
19.18_748.5274m/z	GP02030011	PE(P-38:5)//PE(O-38:6)	748,5274	1	19,175	0,515683	0,003118	1,308149	Bligh	MMC		GP02030	PE(P-38:5)//PE(O-38:6)	M-H	C43H76N	58,7	196,4805	181,4536	188,5905	225,5631	172,4291	225,5631	87,11%	80,44%	83,61%	100,00%	76,44%
19.46_748.5275m/z	GP02030012	PE(P-38:5)//PE(O-38:6)	748,5275	1	19,4633	0,30425	0,000594	1,44893	MTBE	Folch		GP02030	PE(P-38:5)//PE(O-38:6)	M-H	C43H76N	58,7	25,86418	21,16591	30,66794	30,04852	28,41043	30,66794	84,34%	69,02%	100,00%	97,98%	92,64%
18.77_746.5120m/z	GP02030014	PE(P-38:6)//PE(O-38:7)	746,512	1	18,76938	0,341083	0,085292	1,335861	Bligh	Folch		GP02030	PE(P-38:6)//PE(O-38:7)	M-H	C43H74N	56,2	135,6003	122,9615	136,3675	164,2596	138,027	164,2596	82,55%	74,86%	83,02%	100,00%	84,03%
20.01_774.5426m/z	GP02030015	PE(P-40:6)//PE(O-40:7)	774,5426	1	20,00673	0,303483	0,181871	1,265695	Bligh	MMC		GP02030	PE(P-40:6)//PE(O-40:7)	M-H	C45H78N	55,8	109,0863	103,8377	96,25184	120,072	94,86643	120,072	90,85%	86,48%	80,16%	100,00%	79,01%
19.08_772.5275m/z	GP02030016	PE(P-40:7)//PE(O-40:8)	772,5275	1	19,07622	0,37655	1,11E-05	1,773988	Pooled	MMC		GP02030	PE(P-40:7)//PE(O-40:8)	M-H	C45H76N	49,7	80,18361	50,04699	72,64113	70,79487	45,19963	80,18361	100,00%	62,42%	90,59%	88,29%	56,37%
8.97_452.2774m/z	GP02050001	LPE(16:0)	452,2774	1	8,972383	0,368467	0	2,986366	MMC	Bligh		GP02050	LPE(16:0)	M-H	C21H44N	53,8	39,01685	43,53072	41,24722	17,47577	52,18903	52,18903	74,76%	83,41%	79,03%	33,49%	100,00%
8.39_480.3088m/z	GP02050002	LPE(18:0)	480,3088	1	8,389683	0,432	6,40E-07	1,991075	MMC	Bligh		GP02050	LPE(18:0)	M-H	C23H48N	56,9	49,3356	41,63584	50,16707	32,57229	64,85385	64,85385	76,07%	64,20%	77,35%	50,22%	100,00%
8.87_480.3089m/z	GP02050003	LPE(18:0)	480,3089	1	8,8707	0,548417	3,97E-07	1,543876	Folch	Bligh		GP02050	LPE(18:0)	M-H	C23H48N	58,7	413,8662	516,8662	413,6422	334,7847	440,5513	516,8662	80,07%	100,00%	80,03%	64,77%	85,24%
9.56_478.2931m/z	GP02050005	LPE(18:1)	478,2931	1	9,558733	0,31055	3,07E-06	1,809669	MMC	Bligh		GP02050	LPE(18:1)	M-H	C23H46N	56,2	21,31198	20,31587	21,33082	11,88122	21,50107	21,50107	99,12%	94,49%	99,21%	55,26%	100,00%
9.44_506.3244m/z	GP02050007	LPE(20:1)	506,3244	1	9,444967	0,4212	4,31E-06	1,507876	MMC	Pooled		GP02050	LPE(20:1)	M-H	C25H50N	46,4	69,94563	80,16037	77,5892	72,38655	105,4693	105,4693	66,32%	76,00%	73,57%	68,63%	100,00%
8.23_476.2775m/z	GP02050009	LPE(18:2)	476,2775	1	8,2277	0,376233	0	2,803215	MMC	Bligh		GP02050	LPE(18:2)	M-H	C23H44N	56,4	48,58467	52,69916	49,66232	20,18311	56,57759	56,57759	85,87%	93,14%	87,78%	35,67%	100,00%
8.15_504.3087m/z	GP02050010	LPE(20:2)	504,3087	1	8,145233	0,425867	3,53E-11	1,827493	MMC	Bligh		GP02050	LPE(20:2)	M-H	C25H48N	55,4	73,67599	87,62135	75,71771	62,65934	114,5095	114,5095	64,34%	76,52%	66,12%	54,72%	100,00%
8.25_500.2774m/z	GP02050012	LPE(20:4)	500,2774	1	8,2541	0,36115	0	2,306862	MMC	Bligh		GP02050	LPE(20:4)	M-H	C25H44N	49,6	63,99253	65,09532	61,18427	28,83169	66,51074	66,51074	96,21%	97,87%	91,99%	43,35%	100,00%
8.28_524.2779m/z	GP02050017	LPE(22:6)	524,2779	1	8,2807	0,3727	1,28E-12	1,842676	Pooled	Bligh		GP02050	LPE(22:6)	M-H	C27H44N	50,3	70,84718	70,05811	63,5404	38,44798	67,88123	70,84718	100,00%	98,89%	89,69%	54,27%	95,81%
11.93_464.3140m/z	GP02070002	LPE(P-18:0)//LPE(O-18:1)	464,314	1	11,93485	0,113067	2,58E-14	11,22467	MMC	Bligh		GP02070	LPE(P-18:0)//LPE(O-18:1)	M-H	C23H48N	48,2	5,249051	2,541408	4,626367	0,62239	6,986119	6,986119	75,14%	36,38%	66,22%	8,91%	100,00%
17.85_760.5124m/z	GP03010001	PS(34:1)	760,5124	1	17,85295	0,491367	1,02E-12	2,220873	MMC	Folch		GP03010	PS(34:1)	M-H	C40H76N	51,7	49,78649	31,06985	61,01094	38,30845	69,00219	69,00219	72,15%	45,03%	88,42%	55,52%	100,00%
17.62_760.5122m/z	GP03010001	PS(34:1)	760,5122	1	17,62192	0,216183	0	9,049388	MMC	Folch		GP03010	PS(34:1)	M-H	C40H76N	44,6	20,85737	4,10555	26,823	7,977969	37,15272	37,15272	56,14%	11,05%	72,20%	21,47%	100,00%
17.87_786.5279m/z	GP03010004	PS(36:2)	786,5279	1	17,871	0,325433	2,22E-16	6,628517	MMC	Folch		GP03010	PS(36:2)	M-H	C42H78N	42,9	28,4365	6,18278	34,33503	11,28018	40,98266	40,98266	69,39%	15,09%	83,78%	27,52%	100,00%
19.57_834.5256m/z	GP03010012	PS(40:6)	834,5256	1	19,57448	0,3078	0,015363	1,335173	Bligh	Folch		GP03010	PS(40:6)	M-H	C46H78N	51,4	19,26381	18,33425	21,27045	24,4794	20,77483	24,4794	78,69%	74,90%	86,89%	100,00%	84,87%
18.22_830.4941m/z	GPSer(18:2/22:6)	PS(40:8)	830,4941	1	18,22368	0,277633	0,001051	1,734817	Bligh	Pooled		GPSer(18:	PS(40:8)	M-H	C46H74N	36	8,025518	10,77593	8,600552	13,9228	12,99665	13,9228	57,64%	77,40%	61,77%	100,00%	93,35%
7.28_508.2672m/z	GP03010013	LPS(17:1)	508,2672	1	7,284283	0,3618	0	Infinity	MMC	Folch		GP03010	LPS(17:1)	M-H	C23H44N	42,6	28,4819	0	14,80667	0,76006	103,5326	103,5326	27,51%	0,00%	14,30%	0,73%	100,00%
8.26_509.2872m/z	14714413	LPG(18:1)	509,2872	1	8,26165	0,229383	0	Infinity	MMC	Folch		14714413	LPG(18:1)	M-H	C24H47O	34,5	0,713335	0	0,678913	0	8,581098	8,581098	8,31%	0,00%	7,91%	0,00%	100,00%
19.52_777.5637m/z	GP04010003	PG(36:0)	777,5637	1	19,52463	0,262117	0,000137	1,552353	Pooled	Folch		GP04010	PG(36:0)	M-H	C42H83O	49,2	30,66381	19,75312	23,81034	24,47436	24,25898	30,66381	100,00%	64,42%	77,65%	79,82%	79,11%
17.90_773.5329m/z	GP04010016	PG(18:2/18:0)	773,5329	1	17,8978	0,500167	0,002157	1,433931	MTBE	Folch		GP04010	PG(18:2/18:0)	M-H	C42H79O	44,9	926,7156	662,9563	950,6335	861,9355	838,0592	950,6335	97,48%	69,74%	100,00%	90,67%	88,16%
16.24_807.5018m/z	GP06010001	PI(32:1)	807,5018	1	16,23743	0,147167	4,88E-15	2,64318	MTBE	Folch		GP06010	PI(32:1)	M-H	C41H77O	47,9	17,81062	8,215637	21,71541	9,410744	20,03862	21,71541	82,02%	37,83%	100,00%	43,34%	92,28%
17.32_835.5322m/z	GP06010002	PI(34:1)	835,5322	1	17,3183	0,31985	1,11E-16	2,135016	MTBE	Folch		GP06010	PI(34:1)	M-H	C43H81O	58,9	84,99423	53,32833	113,8568	71,91284	113,1125	113,8568	74,65%	46,84%	100,00%	63,16%	99,35%
18.47_863.5648m/z	GP06010004	PI(36:1)	863,5648	1	18,46657	0,3861	4,79E-11	2,230043	MMC	Folch		GP06010	PI(36:1)	M-H	C45H84O	59,9	55,25993	33,77039	61,26333	42,04871	75,3094	75,3094	73,38%	44,84%	81,35%	55,83%	100,00%
16.63_833.5174m/z	GP06010005	PI(34:2)	833,5174	1	16,62753	0,351117	0	1,984149	MTBE	Folch		GP06010	PI(34:2)	M-H	C43H78O	63,4	155,8247	108,0124	214,3126	132,0621	190,3502	214,3126	72,71%	50,40%	100,00%	61,62%	88,82%
17.74_861.5487m/z	GP06010006	PI(36:2)	861,5487	1	17,73888	0,525733	0	2,457757	MTBE	Folch		GP06010	PI(36:2)	M-H	C45H83O	67	264,4538	127,566	313,5262	176,9629	300,9537	313,5262	84,35%	40,69%	100,00%	56,44%	95,99%
16.87_859.5329m/z	GP06010009	PI(36:3)	859,5329	1	16,87367	0,372933	4,98E-13	2,31716	MTBE	Folch		GP06010	PI(36:3)	M-H	C45H81O	56	100,5737	58,91122	136,5067	79,63605	107,2689	136,5067	73,68%	43,16%	100,00%	58,34%	78,58%
18.12_887.5643m/z	GP06010011	PI(38:3)	887,5643	1	18,1177	0,471	6,41E-13	2,107025	MMC	Folch		GP06010	PI(38:3)	M-H	C47H85O	66,3	72,97485	43,96158	91,38303	59,76128	92,62813	92,62813	78,78%	47,46%	98,66%	64,52%	100,00%
18.05_915.5968m/z	GP06010014	PI(40:3)	915,5968	1	18,04885	0,4557	0,001674	1,859344	Bligh	MTBE		GP06010	PI(40:3)	M-H	C45H81O	67	32,34359	47,85995	31,50205	58,57314	31,87902	58,57314	55,22%	81,71%	53,78%	100,00%	54,43%
16.60_857.5167m/z	GP06010015	PI(36:4)	857,5167	1	16,60085	0,346583	4,33E-10	1,682762	MTBE	Folch		GP06010	PI(36:4)	M-H	C45H79O	67,5	167,9687	135,6125	228,2036	171,3157	198,2994	228,2036	73,60%	59,43%	100,00%	75,07%	86,90%
17.69_885.5490m/z	GP06010019	PI(38:4)	885,549	1	17,69408	0,40815	5,89E-12	2,100179	MTBE	Folch		GP06010	PI(38:4)	M-H	C47H83O	72,8	1272,784	663,5334	1393,539	907,5721	1372,426	1393,539	91,33%	47,61%	100,00%	65,13%	98,48%
16.91_883.5322m/z	GP06010023	PI(38:5)	883,5322	1	16,9082	0,432233	5,55E-16	2,089088	MTBE	Folch		GP06010	PI(38:5)	M-H	C47H81O	56	139,7082	91,60884	191,379	132,556	151,907	191,379	73,00%	47,87%	100,00%	69,26%	79,37%
17.92_911.5640m/z	GP06010027	PI(40:5)	911,564	1	17,9205	0,191967	2,15E-05	2,083467	Pooled	Folch		GP06010	PI(40:5)	M-H	C49H85O	52,1	16,90152	8,112209	15,40594	11,75185	10,47033	16,90152	100,00%	48,00%	91,15%	69,53%	61,95%
16.52_881.5183m/z	GP06010029	PI(38:6)	881,5183	1	16,52177	0,2363	5,74E-11	1,760199	MTBE	Folch		GP06010	PI(38:6)	M-H	C47H79O	54,8	22,39417	18,02219	31,72264	26,11726	25,79088	31,72264	70,59%	56,81%	100,00%	82,33%	81,30%
17.60_909.5485m/z	GP06010031	PI(40:6)	909,5485	1	17,60283	0,314317	7,41E-05	1,856176	MTBE	Folch		GP06010	PI(40:6)	M-H	C49H82O	63,6	60,73245	39,0341	72,45416	56,90648	61,09634	72,45416	83,82%	53,87%	100,00%	78,54%	84,32%
7.04_583.2883m/z	GP06050003	LPI(17:1)	583,2883	1	7,043183	0,529317	0	324,8489	MMC	Folch		GP06050	LPI(17:1)	M-H	C26H49O	66	197,2251	1,602439	166,5038	45,51194	520,5504	520,5504	37,89%	0,31%	31,99%	8,74%	100,00%
9.52_599.3193m/z	GP06050003	LPI(18:0)	599,3193	1	9,520267	0,19675	0	Infinity	MMC	Folch		GP06050	LPI(18:0)	M-H	C27H53O	48,6	0,482879	0	0,159537	0	15,71434	15,71434	3,07%	0,00%	1,02%	0,00%	100,00%
15.69_481.4487n	4266350	Cer(d30:0)	526,4468	1	15,68842	0,269917	0,002749	1,456452	Bligh	Folch		4266350	Cer(d30:0)	M-H, M+F	C30H59N	38,4	121,2645	95,23208	124,9132	138,701	103,2153	138,701	87,43%	68,66%	90,06%	100,00%	74,42%
19.93_612.5564m/z	SP02010002	Cer(d36:0)	612,5564	1	19,9303	0,234867	0,000758	1,574004	Bligh	MTBE		SP020100	Cer(d36:0)	M+FA-H	C36H73N	47,9	13,74648	10,93155	9,624642	15,14922	9,954228	15,14922	90,74%	72,16%	63,53%	100,00%	65,71%
22.37_623.6212n	SP02010003	Cer(d40:0)	668,6192	1	22,37103	0,260767	0,000332	1,771989	Bligh	MTBE		SP020100	Cer(d40:0)	M-H, M+F	C40H81N	63,9	71,27944	73,37667	51,13067	90,60298	64,04089	90,60298	78,67%	80,99%	56,43%	100,00%	70,68%
22.94_637.6367n	SP02010004	Cer(d41:0)	682,6346	1	22,94137	0,2475	2,56E-07	1,68137	Bligh	MMC		SP020100	Cer(d41:0)	M-H, M+F	C41H83N	53,3	83,17842	68,96622	65,18435	97,07444	57,73531	97,07444	85,69%	71,04%	67,15%	100,00%	59,48%
23.51_651.6518n	SP02010005	Cer(d42:0)	696,6501	1	23,5098	0,407833	4,40E-10	1,546661	Bligh	MMC		SP020100	Cer(d42:0)	M-H, M+C	C42H85N	54,5	252,4611	192,709	195,857	290,9098	188,0889	290,9098	86,78%	66,24%	67,33%	100,00%	64,66%
16.91_509.4801n	SP02010006	Cer(d32:1)	554,4783	1	16,9082	0,21325	0,019921	1,463784	Bligh	Pooled		SP020100	Cer(d32:1)	M-H, M+F	C32H63N	64,6	44,61953	44,72091	44,95836	65,31335	46,36883	65,31335	68,32%	68,47%	68,83%	100,00%	70,99%
17.54_568.4940m/z	SP02010007	Cer(d33:1)	568,494	1	17,53525	0,193417	9,14E-11	2,139727	Bligh	MMC		SP020100	Cer(d33:1)	M+FA-H	C33H65N	60,6	12,594	16,13746	10,3563	21,38583	9,994651	21,38583	58,89%	75,46%	48,43%	100,00%	46,73%
18.20_537.5113n	SP02010008	Cer(d34:1)	582,5098	1	18,19695	0,3543	0,012026	1,374834	Bligh	Folch		SP020100	Cer(d34:1)	M-H, M+F	C34H67N	72,2	80,35006	78,86278	80,06215	108,4232	83,65821	108,4232	74,11%	72,74%	73,84%	100,00%	77,16%
19.50_565.5425n	SP02010010	Cer(d36:1)	610,5409	1	19,49762	0,292733	0,085414	1,424544	Bligh	Folch		SP020100	Cer(d36:1)	M-H, M+F	C36H71N	64,4	41,04816	34,82259	39,98262	49,60633	42,17039	49,60633	82,75%	70,20%	80,60%	100,00%	85,01%
20.79_593.5742n	SP02010011	Cer(d38:1)	638,5721	1	20,79247	0,3721	2,46E-08	1,562768	Bligh	MMC		SP020100	Cer(d38:1)	M-H, M+F	C38H75N	60,5	216,8747	204,0509	213,5938	295,313	188,968	295,313	73,44%	69,10%	72,33%	100,00%	63,99%
21.99_621.6053n	SP02010013	Cer(d40:1)	666,6037	1	21,98958	0,576933	1,95E-05	1,396547	Bligh	MMC		SP020100	Cer(d40:1)	M-H, M+C	C40H79N	66,4	1202,848	1111,113	1141,285	1375,308	984,7917	1375,308	87,46%	80,79%	82,98%	100,00%	71,61%
22.35_680.6184m/z	SP02010014	Cer(d41:1)	680,6184	1	22,34818	0,15925	0,000717	1,493547	Bligh	MTBE		SP020100	Cer(d41:1)	M+FA-H	C41H81N	52,6	42,30773	44,13532	33,44119	49,94601	38,39544	49,94601	84,71%	88,37%	66,95%	100,00%	76,87%

22.56_635.6208n	SP02010015	Cer(d41:1)	680,6193	1	22,5595	0,38935	4,64E-05	1,391342	Bligh	Folch		SP020100	Cer(d41:1)	M-H, M+F	C41H81N	64	721,6962	642,5406	689,7821	893,9938	683,2438	893,9938	80,73%	71,87%	77,16%	100,00%	76,43%
22.59_670.5903m/z	SP02010015	Cer(d41:1)	670,5903	1	22,5862	0,146983	0,003442	1,553532	Bligh	Folch		SP020100	Cer(d41:1)	M+Cl	C41H81N	53,1	8,165707	7,620026	9,071897	11,83795	9,607163	11,83795	68,98%	64,37%	76,63%	100,00%	81,16%
23.13_649.6365n	SP02010016	Cer(d42:1)	694,635	1	23,13138	1,003283	6,68E-09	1,473539	Bligh	Folch		SP020100	Cer(d42:1)	M-H, M+C	C42H83N	57,5	2842,41	2486,446	2825,907	3663,875	2499,337	3663,875	77,58%	67,86%	77,13%	100,00%	68,22%
23.49_663.6521n	SP02010017	Cer(d43:1)	708,6502	1	23,48735	0,225217	3,79E-05	1,43487	Bligh	MMC		SP020100	Cer(d43:1)	M-H, M+C	C43H85N	55,1	254,8124	219,5731	234,4433	306,9056	213,8909	306,9056	83,03%	71,54%	76,39%	100,00%	69,69%
23.66_663.6523n	SP02010018	Cer(d43:1)	708,6502	1	23,66348	0,337333	3,78E-09	1,667139	Bligh	MMC		SP020100	Cer(d43:1)	M-H, M+C	C43H85N	55,7	838,1362	766,4208	743,0417	1056,568	633,761	1056,568	79,33%	72,54%	70,33%	100,00%	59,98%
24.17_722.6656m/z	SP02010019	Cer(d44:1)	722,6656	1	24,1721	0,24865	2,63E-14	1,98025	Bligh	MMC		SP020100	Cer(d44:1)	M+FA-H	C44H87N	54,8	118,9036	88,0132	89,28373	123,3547	62,2925	123,3547	96,39%	71,35%	72,38%	100,00%	50,50%
17.24_535.4958n	SP02010020	Cer(d34:2)	580,494	1	17,24448	0,2271	0,165276	1,202056	Bligh	MMC		SP020100	Cer(d34:2)	M-H, M+F	C34H65N	58,7	27,30612	25,5322	24,59179	27,48513	22,86509	27,48513	99,35%	92,89%	89,47%	100,00%	83,19%
18.52_608.5254m/z	SP02010021	Cer(d36:2)	608,5254	1	18,5235	0,229917	6,13E-05	1,595057	Bligh	MTBE		SP020100	Cer(d36:2)	M+FA-H	C36H69N	51,3	10,93708	9,286933	8,996343	14,34968	9,09713	14,34968	76,22%	64,72%	62,69%	100,00%	63,40%
20.98_619.5898n	SP02010022	Cer(d40:2)	664,5877	1	20,9756	0,1647	0,015876	1,320847	Bligh	Folch		SP020100	Cer(d40:2)	M-H, M+F	C40H77N	62,4	126,7094	103,887	128,6598	137,2189	123,7981	137,2189	92,34%	75,71%	93,76%	100,00%	90,22%
21.12_619.5895n	SP02010023	Cer(d40:2)	664,5876	1	21,11768	0,299083	0,102364	1,206387	Bligh	MMC		SP020100	Cer(d40:2)	M-H, M+F	C40H77N	51,7	193,2683	183,0519	203,256	213,9201	177,3229	213,9201	90,35%	85,57%	95,01%	100,00%	82,89%
21.69_633.6056n	SP02010024	Cer(d41:2)	678,6037	1	21,6884	0,57855	0,000617	1,424573	Bligh	Folch		SP020100	Cer(d41:2)	M-H, M+F	C41H79N	53,3	151,0337	150,1136	154,7805	213,8479	165,3344	213,8479	70,63%	70,20%	72,38%	100,00%	77,31%
21.53_633.6051n	SP02010024	Cer(d41:2)	678,6034	1	21,53107	0,237733	0,054028	1,318179	Bligh	MMC		SP020100	Cer(d41:2)	M-H, M+F	C41H79N	65,7	94,94788	103,5983	101,1608	119,9911	91,02796	119,9911	79,13%	86,34%	84,31%	100,00%	75,86%
22.14_692.6192m/z	SP02010026	Cer(d42:2)	692,6192	1	22,14403	0,91155	0,005459	1,285099	Bligh	Folch		SP020100	Cer(d42:2)	M+FA-H	C42H81N	69,5	2113,576	1826,486	1868,228	2347,215	1890,753	2347,215	90,05%	77,82%	79,59%	100,00%	80,55%
22.14_647.6208n	SP02010026	Cer(d42:2)	646,6135	1	22,14403	0,460783	0,010475	1,387902	Bligh	Folch		SP020100	Cer(d42:2)	M-H, M+C	C42H81N	71,5	282,7182	226,242	248,324	314,0017	251,8782	314,0017	90,04%	72,05%	79,08%	100,00%	80,22%
22.49_661.6360n	SP02010028	Cer(d43:2)	706,6345	1	22,49242	0,215167	0,000213	1,614811	Bligh	Pooled		SP020100	Cer(d43:2)	M-H, M+F	C43H83N	54,8	39,4982	45,65663	49,93453	63,78214	50,57643	63,78214	61,93%	71,58%	78,29%	100,00%	79,30%
22.65_661.6367n	SP02010029	Cer(d43:2)	706,6347	1	22,6541	0,171633	5,26E-05	1,375686	Bligh	MMC		SP020100	Cer(d43:2)	M-H, M+F	C43H83N	51,4	135,3319	117,6992	119,903	157,135	114,2231	157,135	86,12%	74,90%	76,31%	100,00%	72,69%
23.23_675.6517n	SP02010030	Cer(d44:2)	720,6499	1	23,22567	0,3254	1,26E-10	1,788172	Bligh	MMC		SP020100	Cer(d44:2)	M-H, M+F	C44H85N	53,8	85,02064	60,89707	73,17436	94,68272	52,94945	94,68272	89,80%	64,32%	77,28%	100,00%	55,92%
21.26_645.6051n	SP02010031	Cer(d42:3)	690,6033	1	21,26173	0,398217	0,00443	1,493947	Bligh	Folch		SP020100	Cer(d42:3)	M-H, M+F	C42H79N	69,4	181,8777	162,1462	187,7395	242,2378	190,124	242,2378	75,08%	66,94%	77,50%	100,00%	78,49%
21.34_684.6136m/z	SP02010032	Cer(t40:0)	684,6136	1	21,33868	0,273233	4,70E-08	1,892297	Bligh	MMC		SP020100	Cer(t40:0)	M+FA-H	C40H81N	54,8	14,69296	9,150116	10,69389	16,54358	8,742596	16,54358	88,81%	55,31%	64,64%	100,00%	52,85%
22.47_667.6469n	SP02010034	Cer(t42:0)	712,645	1	22,47333	0,312567	5,05E-06	1,683754	Bligh	MMC		SP020100	Cer(t42:0)	M-H, M+F	C42H85N	50,8	35,05228	32,07331	32,11712	45,46689	27,00329	45,46689	77,09%	70,54%	70,64%	100,00%	59,39%
16.71_721.5491m/z	SP03010001	SM(d32:0)	721,5491	1	16,7062	0,122317	0,110685	1,358375	Folch	MMC		SP030100	SM(d32:0)	M+FA-H	C37H77N2	52,8	9,495903	11,00712	9,533283	8,492183	8,103153	11,00712	86,27%	100,00%	86,61%	77,15%	73,62%
17.95_749.5803m/z	SP03010002	SM(d34:0)	749,5803	1	17,95475	0,260783	0,280763	1,276923	Pooled	MTBE		SP030100	SM(d34:0)	M+FA-H	C39H81N2	73,8	102,961	94,88187	80,63211	102,9358	96,42819	102,961	100,00%	92,15%	78,31%	99,98%	93,66%
19.23_777.6102m/z	SP03010003	SM(d36:0)	777,6102	1	19,23245	0,235017	9,50E-05	3,281344	Pooled	MMC		SP030100	SM(d36:0)	M+FA-H	C41H85N2	51,8	6,481423	4,362842	3,855139	4,194228	1,975234	6,481423	100,00%	67,31%	59,48%	64,71%	30,48%
21.68_833.6738m/z	SP03010005	SM(d40:0)	833,6738	1	21,68103	0,491733	1,90E-05	2,111351	Bligh	MMC		SP030100	SM(d40:0)	M+FA-H	C45H93N2	50,6	10,65688	8,388034	6,678791	10,98313	5,201944	10,98313	97,03%	76,37%	60,81%	100,00%	47,36%
15.04_691.5022m/z	SP03010007	SM(d30:1)	691,5022	1	15,04367	0,2782	1,66E-06	1,461605	MTBE	Folch		SP030100	SM(d30:1)	M+FA-H	C35H71N2	68,2	53,37566	41,63981	60,86094	43,51656	52,06939	60,86094	87,70%	68,42%	100,00%	71,50%	85,55%
16.22_674.5354n	SP03010009	SM(d32:1)	719,5336	1	16,22225	0,410567	0,000106	1,281598	Bligh	MTBE		SP030100	SM(d32:1)	M+FA-H, 2	C37H75N2	75,4	568,2787	666,6362	534,8125	685,4147	582,5525	685,4147	82,91%	97,26%	78,03%	100,00%	84,99%
16.84_733.5489m/z	SP03010010	SM(d33:1)	733,5489	1	16,83633	0,313733	0,488016	1,151442	Folch	Pooled		SP030100	SM(d33:1)	M+FA-H	C38H77N2	68,5	81,48757	93,82822	89,23262	92,50618	86,50984	93,82822	86,85%	100,00%	95,10%	98,59%	92,20%
17.43_702.5669n	SP03010011	SM(d34:1)	747,5651	1	17,42912	0,655183	0,035312	1,210703	Bligh	Pooled		SP030100	SM(d34:1)	M+FA-H, 2	C39H79N2	70,7	1440,199	1590,933	1456,764	1743,654	1630,967	1743,654	82,60%	91,24%	83,55%	100,00%	93,54%
18.74_715.5750m/z	123068755	SM(d35:1)	715,575	1	18,73507	0,378217	0,343173	1,28785	Bligh	MTBE		1,23E+08	SM(d35:1)	M-H	C40H81N2	37,6	48,12566	52,30029	43,55914	56,09764	52,78258	56,09764	85,79%	93,23%	77,65%	100,00%	94,09%
17.87_761.5785m/z	SP03010012	SM(d35:1)	761,5785	1	17,871	0,12455	0,107107	1,475014	Folch	MTBE		SP030100	SM(d35:1)	M+FA-H	C40H81N2	52,4	4,17352	4,844087	3,284096	4,611092	3,760057	4,844087	86,16%	100,00%	67,80%	95,19%	77,62%
18.09_761.5802m/z	SP03010013	SM(d35:1)	761,5802	1	18,09097	0,238633	0,611807	1,145234	MMC	MTBE		SP030100	SM(d35:1)	M+FA-H	C40H81N2	74,2	24,44637	23,27394	22,85528	25,85143	26,17464	26,17464	93,40%	88,92%	87,32%	98,77%	100,00%
18.73_730.5978n	SP03010014	SM(d36:1)	775,596	1	18,72748	0,45065	0,390304	1,248269	Bligh	MTBE		SP030100	SM(d36:1)	M+FA-H, 2	C41H83N2	75,5	324,5366	374,4758	309,5824	386,442	352,4424	386,442	83,98%	96,90%	80,11%	100,00%	91,20%
19.37_789.6117m/z	SP03010015	SM(d37:1)	789,6117	1	19,36878	0,19265	0,004104	1,496157	Pooled	MMC		SP030100	SM(d37:1)	M+FA-H	C42H85N2	51	5,792182	5,4763	4,651864	4,954601	3,871374	5,792182	100,00%	94,55%	80,31%	85,54%	66,84%
20.01_803.6270m/z	SP03010016	SM(d38:1)	803,627	1	20,00673	0,442917	0,618119	1,235558	Bligh	MTBE		SP030100	SM(d38:1)	M+FA-H	C43H87N2	74,2	212,0826	227,8302	192,236	237,5187	223,7547	237,5187	89,29%	95,92%	80,94%	100,00%	94,21%
20.63_817.6427m/z	SP03010017	SM(d39:1)	817,6427	1	20,63358	0,335333	0,012474	1,437173	Bligh	MTBE		SP030100	SM(d39:1)	M+FA-H	C44H89N2	57,1	143,8314	143,0327	115,0091	165,2879	119,6359	165,2879	87,02%	86,54%	69,58%	100,00%	72,38%
21.81_845.6742m/z	SP03010019	SM(d41:1)	845,6742	1	21,80933	1,08555	0,020051	1,277188	Bligh	MTBE		SP030100	SM(d41:1)	M+FA-H	C46H93N2	72	358,9796	385,071	339,2784	433,3223	342,5963	433,3223	82,84%	88,86%	78,30%	100,00%	79,06%
21.37_797.6533m/z	SP03010020	SM(d41:2)	797,6533	1	21,36833	0,607867	0,023477	1,261356	MMC	Pooled		SP030100	SM(d41:2)	M-H	C46H91N2	55,5	165,5966	172,8607	171,452	207,8106	208,8764	208,8764	79,28%	82,76%	82,08%	99,49%	100,00%
21.82_859.6891m/z	SP03010021	SM(d42:1)	859,6891	1	21,82453	0,304217	0,034336	1,33127	Bligh	Pooled		SP030100	SM(d42:1)	M+FA-H	C47H95N2	53,8	24,10196	27,25517	27,81545	32,08622	30,08715	32,08622	75,12%	84,94%	86,69%	100,00%	93,77%
22.36_859.6898m/z	SP03010022	SM(d42:1)	859,6898	1	22,36342	0,65165	0,010158	1,345915	Bligh	MTBE		SP030100	SM(d42:1)	M+FA-H	C47H95N2	76,9	394,7476	421,4092	360,6931	485,4621	406,5539	485,4621	81,31%	86,81%	74,30%	100,00%	83,75%
22.73_873.7052m/z	SP03010023	SM(d43:1)	873,7052	1	22,72837	0,21	2,07E-07	1,806038	Bligh	MMC		SP030100	SM(d43:1)	M+FA-H	C48H97N2	52,4	26,2456	19,82309	23,75618	28,11958	15,56976	28,11958	93,34%	70,50%	84,48%	100,00%	55,37%
22.96_873.7055m/z	SP03010024	SM(d43:1)	873,7055	1	22,95607	0,198883	1,35E-07	2,773763	Pooled	MMC		SP030100	SM(d43:1)	M+FA-H	C48H97N2	52,8	18,32965	13,00841	11,79387	17,90446	6,608223	18,32965	100,00%	70,97%	64,34%	97,68%	36,05%
15.35_717.5177m/z	SP03010025	SM(d32:2)	717,5177	1	15,35397	0,20525	0,002252	1,320258	MTBE	Bligh		SP030100	SM(d32:2)	M+FA-H	C37H73N2	52,2	35,95651	31,9539	37,24413	28,20974	32,48189	37,24413	96,54%	85,80%	100,00%	75,74%	87,21%
17.54_745.5514m/z	123068752	SM(d34:2)	745,5514	1	17,54285	0,49195	0,000477	2,351104	Pooled	MMC		1,23E+08	SM(d34:2)	M+FA-H	C39H77N2	36,4	5,829425	5,326715	4,842197	4,853536	2,479442	5,829425	100,00%	91,38%	83,06%	83,26%	42,53%
16.57_700.5510n	SP03010026	SM(d34:2)	745,5492	1	16,56658	0,4297	1,44E-06	1,361399	Bligh	Pooled		SP030100	SM(d34:2)	M+FA-H, 2	C39H77N2	75,5	641,4973	817,114	742,1294	873,334	715,2057	873,334	73,45%	93,56%	84,98%	100,00%	81,89%
17.82_773.5801m/z	SP03010028	SM(d36:2)	773,5801	1	17,81502	0,316583	0,252544	1,236388	Bligh	Pooled		SP030100	SM(d36:2)	M+FA-H	C41H81N2	76,4	224,1733	260,9041	230,4956	277,1652	256,993	277,1652	80,88%	94,13%	83,16%	100,00%	92,72%
19.08_801.6115m/z	SP03010029	SM(d38:2)	801,6115	1	19,07622	0,338333	0,135726	1,225011	Pooled	Folch		SP030100	SM(d38:2)	M+FA-H	C43H85N2	71,2	53,32403	43,52944	53,26634	47,9206	45,72071	53,32403	100,00%	81,63%	99,89%	89,87%	85,74%
20.33_769.6215m/z	123068775	SM(d39:2)	769,6215	1	20,32707	0,214217	0,046351	1,21913	Bligh	Folch		1,23E+08	SM(d39:2)	M-H	C44H87N2	36,3	31,90032	31,50877	33,0526	38,41329	37,63369	38,41329	83,05%	82,03%	86,04%	100,00%	97,97%
20.33_829.6426m/z	SP03010030	SM(d40:2)	829,6426	1	20,32707	0,551483	0,010078	1,254263	Bligh	Pooled		SP030100	SM(d40:2)	M+FA-H	C45H89N2	61,9	362,9205	399,0488	390,9219	455,1977	445,6506	455,1977	79,73%	87,66%	85,88%	100,00%	97,90%
20.95_843.6582m/z	SP03010032	SM(d41:2)	843,6582	1	20,94885	0,33055	0,053409	1,170988	Bligh	Folch		SP030100	SM(d41:2)	M+FA-H	C46H91N2	61,5	241,1522	237,4927	260,6315	278,101	275,4546	278,101	86,71%	85,40%	93,72%	100,00%	99,05%
21.37_812.6758n	SP03010034	SM(d42:2)	857,674	1	21,36833	0,777717	0,037464	1,280114	Bligh	Pooled		SP030100	SM(d42:2)	M+FA-H, 2	C47H93N2	74,5	1097,902	1195,683	1174,622	1405,44	1350,066	1405,44	78,12%	85,08%	83,58%	100,00%	96,06%
22.13_871.6898m/z	123068794	SM(d43:2)	871,6898	1	22,12598	0,71515	2,53E-11	6,993579	Folch	MMC		1,23E+08	SM(d43:2)	M+FA-H	C48H95N2	36,3	9,138219	25,16591	5,27988	6,049708	3,598431	25,16591	36,31%	100,00%	20,98%	24,04%	14,30%
21.73_871.6900m/z	SP03010036	SM(d43:2)	871,69	1	21,73003	0,236383	0,004073	1,46434	Bligh	Pooled		SP030100	SM(d43:2)	M+FA-H	C48H95N2	71,7	49,98443	58,96242	57,80072	73,19422	68,62957	73,19422	68,29%	80,56%	78,97%	100,00%	93,76%
19.32_827.6270m/z	123068781	SM(d40:3)	827,627	1	19,31542	0,295833	0,031354	1,369163	Bligh	Folch		1,23E+08	SM(d40:3)	M+FA-H	C45H87N2	33,7	18,08917	15,56966	19,33366	21,31739	18,35494	21,31739	84,86%	73,04%	90,69%	100,00%	86,10%
20.48_810.6602n	SP03010041	SM(d42:3)	855,6584	1	20,48203	0,5738	0,091209	1,247308	Bligh	Pooled		SP030100	SM(d42:3)	M+FA-H, 2	C47H91N2	74,6	658,6029	732,5301	664,5838	821,4805	723,4568	821,4805	80,17%	89,17%	80,90%	100,00%	88,07%
21.67_883.6890m/z	SP03010042	SM(d44:3)	883,689	1	21,6663	0,178983	0,019819	1,957785	Bligh	MMC		SP030100	SM(d44:3)	M+FA-H	C49H95N2	44,3	3,235491	3,38458	3,816536	5,355574	2,735528	5,355574	60,41%	63,20%	71,26%	100,00%	51,08%
20.69_875.6845m/z	SP03010045	SM(t42:1)	875,6845	1	20,68967	0,173733	0,250367	1,369362	Bligh	MMC		SP030100	SM(t42:1)	M+FA-H	C47H95N2	48,1	7,636664	7,568111	6,692422	7,63758	5,577471	7,63758	99,99%	99,09%	87,62%	100,00%	73,03%
21.01_1246.8007n	7983967	14:1(3)-15:1 CL	1247,808	1	21,00777	0,651117	6,89E-08	1,399854	Pooled	Folch		7983967	14:1(3)-15:1 CA	M+H, M+N	C66H120	40,3	1339,686	957,0184	1274,632	1152,606	1284,785	1339,686	100,00%	71,44%	95,14%	86,04%	95,90%
24.51_1308.9104n	7983968	15:0(3)-16:1 CL	1309,918	1	24,51372	0,426467	4,15E-06	1,568048	Pooled	Folch		7983968	15:0(3)-16:1 CA	M+H, M+N	C70H134	41,4	2487,73	1586,513	2125,916	2373,986	1659,861	2487,73	100,00%	63,77%	85,46%	95,43%	66,72%
30.82_1569.1609n	7983969	22:1(3)-14:1 CL	1570,168	1	30,8238	1,142233	0,000722	1,440026	Bligh	Folch		7983969	22:1(3)-14:1 CA	M+H, M+N	C89H166	40,8	2197,465	1676,737	2100,42	2414,546	1990,474	2414,546	91,01%	69,44%	86,99%	100,00%	82,44%
14.88_470.3612n	GL03010001	TG(24:0)	493,3504	1	14,8837	0,201683	0,003515	1,579985	MMC	Folch	x	GL030100	TG(24:0)	M+NH4, M	C27H50O	55,2	37,93238	26,27606	37,08677	31,54171	41,51579	41,51579	91,37%	63,29%	89,33%	75,98%	100,00%
22.63_638.5497n	GL03010003	TG(36:0)	661,5385	1	22,63498	0,222467	0,051701	1,802495	MMC	MTBE		GL030100	TG(36:0)	M+NH4, M	C39H74O	49,9	9,632958	9,665954	9,253549	9,430557	16,67948	16,67948	57,75%	57,95%	55,48%	56,54%	100,00%
24.82_694.6119n	GL03010005	TG(40:0)	712,6457	1	24,8152	0,267433	0,001104	1,384907	MMC	Pooled		GL030100	TG(40:0)	M+NH4, M	C43H86N	49,8	156,3078	191,1254	158,9545	169,7083	216,4718	216,4718	72,21%	88,29%	73,43%	78,40%	100,00%
25.78_722.6428n	GL03010006	TG(42:0)	740,6766	1	25,78063	0,277	0,004649	1,400179	MMC	MTBE	x	GL030100	TG(42:0)	M+NH4, M	C45H90N	54,2	211,1191	188,0101	179,2087	214,0107	250,9242	250,9242	84,14%	74,93%	71,42%	85,29%	100,00%
26.25_754.6925m/z	GL03010008	TG(43:0)	754,6925	1	26,2544	0,2543	0,064534	2,006281	MMC	Bligh		GL030100	TG(43:0)	M+NH4	C46H88O	48,2	4,72454	4,646155	5,099024	4,261634	8,550033	8,550033	55,26%	54,34%	59,64%	49,84%	100,00%
26.70_750.6740n	GL03010009	TG(44:0)	768,7079	1	26,70338	0,453083	3,09E-06	1,41286	MMC	MTBE	x	GL030100	TG(44:0)	M+NH4, M	C47H94N	57,2	260,0441	276,2777	232,3256	268,1827	328,2436	328,2436	79,22%	84,17%	70,78%	81,70%	100,00%
27.18_782.7237m/z	GL03010011	TG(45:0)	782,7237	1	27,17703	0,493167	8,15E-06	1,501879	MMC	MTBE		GL030100	TG(45:0)	M+NH4	C48H92O	48,7	33,72044	37,21382	29,46051	33,21251	44,24612	44,24612	76,21%	84,11%	66,58%	75,06%	100,00%
27.86_778.7056n	GL03010012	TG(46:0)	796,7394	1	27,86165	0,4591	0,021404	1,246176	MMC	MTBE	x	GL030100	TG(46:0)	M+NH4, M	C49H98N	57,2	476,9794	451,7373	414,0975	473,1808	516,0382	516,0382	92,43%	87,54%	80,25%	91,69%	100,00%
28.50_810.7553m/z	GL03010014	TG(47:0)	810,7553	1	28,49817	0,596233	0,003202	1,304105	MMC	MTBE		GL030100	TG(47:0)	M+NH4	C50H100N	50,5	49,50201	44,78563	40,78673	47,99232	53,1902	53,1902	93,07%	84,20%	76,68%	90,23%	100,00%
29.26_806.7373n	GL03010015	TG(48:0)	824,7712	1	29,25885	0,787517	0,036644	1,22593	Pooled	Folch	x	GL030100	TG(48:0)	M+NH4, M	C51H102N	68,8	1924,556	1569,875	1597,622	1826,494	1842,931	1924,556	100,00%	81,57%	83,01%	94,90%	95,76%
31.02_834.7682n	GL03010016	TG(50:0)	852,802	1	31,02253	1,126667	0,556546	1,182877	Bligh	Pooled	x	GL030100	TG(50:0)	M+NH4, M	C53H106N	62,3	2867,958	3145,521	3017,789	3392,441	3262,481	3392,441	84,54%	92,72%	88,96%	100,00%	96,17%
31.67_866.8174m/z	GL03010017	TG(51:0)	866,8174	1	31,66815	0,333667	0,312447	1,309042	MMC	Folch	x	GL030100	TG(51:0)	M+NH4	C54H104	51,6	50,63776	49,69285	51,91887	50,53475	65,05002	65,05002	77,84%	76,39%	79,81%	77,69%	100,00%
32.06_866.8171m/z	GL03010018	TG(51:0)	866,8171	1	32,0564	0,434783	0,155415	1,614405	MMC	Pooled	x	GL030100	TG(51:0)	M+NH4	C54H104	54,5	40,01348	48,18682	43,32112	48,4085	64,59795	64,59795	61,94%	74,59%	67,06%	74,94%	100,00%
33.24_862.7989n	GL03010019	TG(52:0)	885,7886	1	33,24207	0,879817	0,202096	1,456719	MMC	Pooled		GL030100	TG(52:0)	M+NH4, M	C55H110N	48,8	1007,656	1141,153	1106,089	1244,987	1467,871	1467,871	68,65%	77,74%	75,35%	84,82%	100,00%
21.75_636.5337n	GL03010021	TG(36:1)	654,5675	1	21,75142	0,265467	1,73E-05	1,532044	MMC	Pooled	x	GL030100	TG(36:1)	M+NH4, M	C39H72O	51,5	37,85788	41,25935	40,03409	41,59797	57,99993	57,99993	65,27%	71,14%	69,02%	71,72%	100,00%
22.91_664.5649n	GL03010022	TG(38:1)	687,5542	1	22,90527	0,319533	2,60E-05	1,591581	MMC	Folch	x	GL030100	TG(38:1)	M+NH4, M	C41H76O	54,6	89,52115	87,79323	94,13904	90,22574	139,73	139,73	64,07%	62,83%	67,37%	64,57%	100,00%
23.21_664.5646n	GL03010023	TG(38:1)	687,5542	1	23,21428	0,22395	0,001404	1,883823	MMC	Folch	x	GL030100	TG(38:1)	M+NH4, M	C41H76O	52,6	29,28124	17,57286	24,00759	28,16253	33,10417	33,10417	88,45%	53,08%	72,52%	85,07%	100,00%
24.03_692.5961n	GL03010024	TG(40:1)	715,5852	1	24,02627	0,39385	0,000354	1,418979	MMC	Folch	x	GL030100	TG(40:1)	M+NH4, M	C43H80O	53,4	178,0004	157,5641	164,5465	167,5068	223,5802	223,5802	79,61%	70,47%	73,60%	74,92%	100,00%
25.04_720.6275n	GL03010025	TG(42:1)	738,6613	1	25,0362	0,3626	0,554898	1,154974	Bligh	Folch	x	GL030100	TG(42:1)	M+NH4, M	C45H88N	50,1	695,3901	677,1745	713,8866	782,1191	748,1911	782,1191	88,91%	86,58%	91,28%	100,00%	95,66%
25.48_734.6430n	GL03010026	TG(43:1)	752,6768	1	25,47893	0,199033	0,213234	1,412815	MMC	Pooled	x	GL030100	TG(43:1)	M+NH4, M	C46H90N	51,4	12,82537	15,64819	15,07149	14,86533	18,11988	18,11988	70,78%	86,36%	83,18%	82,04%	100,00%
25.90_748.6586n	GL03010027	TG(44:1)	766,6924	1	25,89952	0,450483	0,000977	1,221864	MMC	Pooled	x	GL030100	TG(44:1)	M+NH4, M	C47H92N	54	724,3694	830,7175	737,7883	763,9289	885,0805	885,0805	81,84%	93,86%	83,36%	86,31%	100,00%
26.43_780.7078m/z	GL03010028	TG(45:1)	780,7078	1	26,43242	0,502967	0,118286	1,244289	MMC	Bligh		GL030100	TG(45:1)	M+NH4	C48H94N	48,2	35,72226	36,25958	39,9944	35,5296	44,2091	44,2091	80,80%	82,02%	90,47%	80,37%	100,00%
26.90_776.6901n	GL03010029	TG(46:1)	794,724	1	26,89738	0,685833	0,00032	1,17312	MMC	Pooled	x	GL030100	TG(46:1)	M+NH4, M	C49H96N	54,4	1467,632	1600,033	1525,854	1479,811	1721,708	1721,708	85,24%	92,93%	88,62%	85,95%	100,00%
27.48_790.7055n	GL03010030	TG(47:1)	808,7393	1	27,47563	0,59555	0,000157	1,196874	MMC	Pooled	x	GL030100	TG(47:1)	M+NH4, M	C50H98N	50	196,3195	223,0493	215,3901	212,5477	234,9697	234,9697	83,55%	94,93%	91,67%	90,46%	100,00%
28.73_818.7376n	GL03010033	TG(49:1)	836,7714	1	28,72552	0,944267	0,004057	1,193946	Folch	Pooled	x	GL030100	TG(49:1)	M+NH4, M	C52H102N	51,6	646,1738	771,4968	700,4	688,995	717,2575	771,4968	83,76%	100,00%	90,78%	89,31%	92,97%
29.52_832.7530n	GL03010034	TG(50:1)	850,7868	1	29,51678	0,7727	3,15E-05	1,170556	Folch	Pooled	x	GL030100	TG(50:1)	M+H, M+N	C53H104N	63,1	13748,63	16093,54	14651,74	14426,91	15717,77	16093,54	85,43%	100,00%	91,04%	89,64%	97,67%
30.04_864.8016m/z	GL03010035	TG(51:1)	864,8016	1	30,03732	0,380517	0,747967	1,109557	Bligh	Pooled	x	GL030100	TG(51:1)	M+NH4	C54H106N	57,2	399,6607	417,8842	407,5549	443,4461	422,3927	443,4461	90,13%	94,24%	91,91%	100,00%	95,25%
30.33_846.7676n	GL03010036	TG(51:1)	864,8014	1	30,33108	0,735217	0,001856	1,304482	Folch	Pooled	x	GL030100	TG(51:1)	M+NH4, M	C54H106N	55,2	1203,442	1569,868	1401,788	1241,298	1442,158	1569,868	76,66%	100,00%	89,29%	79,07%	91,86%
31.34_860.7839n	GL03010037	TG(52:1)	878,8177	1	31,34492	1,4416	2,81E-06	1,44132	Folch	Bligh	x	GL030100	TG(52:1)	M+NH4, M	C55H108N	63,6	16367,44	23477,93	17697,13	16289,19	20658,59	23477,93	69,71%	100,00%	75,38%	69,38%	87,99%
32.03_874.7992n	GL03010038	TG(53:1)	892,8331	1	32,03292	0,423133	0,008262	1,553691	MMC	Pooled	x	GL030100	TG(53:1)	M+NH4, M	C56H110N	52,1	135,451	199,392	196,5826	154,1029	210,4489	210,4489	64,36%	94,75%	93,41%	73,23%	100,00%
32.42_874.7990n	GL03010039	TG(53:1)	897,788	1	32,42415	0,945583	0,020521	1,428417	Folch	Bligh	x	GL030100	TG(53:1)	M+NH4, M	C56H110N	55,2	399,1493	498,4573	395,8986	348,9578	436,9464	498,4573	80,08%	100,00%	79,42%	70,01%	87,66%
33.61_888.8151n	GL03010040	TG(54:1)	906,8489	1	33,6073	1,380217	0,004953	1,367249	Folch	Bligh	x	GL030100	TG(54:1)	M+NH4, M	C57H112N	56,9	3182,791	4248,561	3617,339	3107,379	4053,714	4248,561	74,91%	100,00%	85,14%	73,14%	95,41%
22.06_680.5831m/z	GL03010044	TG(38:2)	680,5831	1	22,058	0,2367	0,000305	2,492653	MMC	MTBE	x	GL030100	TG(38:2)	M+NH4	C41H74O	51,9	6,353343	6,286347	2,931893	4,437833	7,308191	7,308191	86,93%	86,02%	40,12%	60,72%	100,00%
25.25_746.6429n	GL03010047	TG(44:2)	764,6767	1	25,24945	0,480183	0,153799	1,165037	MMC	Pooled	x	GL030100	TG(44:2)	M+NH4, M	C47H90N	59,1	1066,406	1094,703	1091,129	1083,579	1242,403	1242,403	85,83%	88,11%	87,82%	87,22%	100,00%
26.11_774.6744n	GL03010048	TG(46:2)	792,7083	1	26,108	0,570867	0,003033	1,19012	MMC	Pooled	x	GL030100	TG(46:2)	M+NH4, M	C49H94N	51,3	1496,131	1589,08	1693,446	1687,464	1780,575	1780,575	84,03%	89,25%	95,11%	94,77%	100,00%
27.12_802.7060n	GL03010049	TG(48:2)	820,7395	1	27,11772	0,8468	0,004438	1,116734	MMC	Pooled		GL030100	TG(48:2)	M+NH4, M	C51H98N	48	4939,595	5402,463	5411,697	5142,772	5516,216	5516,216	89,55%	97,94%	98,11%	93,23%	100,00%
27.63_834.7552m/z	GL03010050	TG(49:2)	834,7552	1	27,62717	0,9109	0,000166	1,129572	MMC	Pooled		GL030100	TG(49:2)	M+NH4	C52H100N	46,7	495,8891	555,4617	546,3305	546,5311	560,1422	560,1422	88,53%	99,16%	97,53%	97,57%	100,00%
28.27_830.7373n	GL03010051	TG(50:2)	848,7712	1	28,27063	0,965017	0,017942	1,12743	Folch	Pooled	x	GL030100	TG(50:2)	M+NH4, M	C53H102N	59,2	14695,79	16568,47	15979,1	16142,41	16424,06	16568,47	88,70%	100,00%	96,44%	97,43%	99,13%
29.04_844.7521n	GL03010052	TG(51:2)	862,7866	1	29,03742	0,9756	0,075284	1,132903	Folch	Pooled		GL030100	TG(51:2)	M+NH4, M	C54H104N	47,8	2545,508	2883,815	2795,848	2780,365	2843,364	2883,815	88,27%	100,00%	96,95%	96,41%	98,60%
29.81_858.7683n	GL03010053	TG(52:2)	881,7575	1	29,81412	1,9885	0,268291	1,178537	Bligh	Pooled	x	GL030100	TG(52:2)	M+Na, M+	C55H106N	56,6	3733,713	4310,023	3928,718	4400,319	3857,385	4400,319	84,85%	97,95%	89,28%	100,00%	87,66%
29.74_858.7687n	GL03010053	TG(52:2)	876,8025	1	29,74053	1,137533	0,02298	1,216394	Folch	Pooled	x	GL030100	TG(52:2)	M+H, M+N	C55H106N	60,8	48119,13	58531,82	54486,43	54413,55	55050,77	58531,82	82,21%	100,00%	93,09%	92,96%	94,05%
30.61_872.7838n	GL03010055	TG(53:2)	890,8176	1	30,61445	1,319183	0,000193	1,446862	Folch	Pooled	x	GL030100	TG(53:2)	M+NH4, M	C56H108N	54,2	4337,254	6275,408	6096,629	5217,315	6030,583	6275,408	69,12%	100,00%	97,15%	83,14%	96,10%
31.64_886.7996n	GL03010057	TG(54:2)	904,8335	1	31,64417	1,727867	0,000284	1,410359	MMC	Pooled	x	GL030100	TG(54:2)	M+H, M+N	C57H110N	70,4	29644,48	41593,37	41185,37	36541,67	41809,35	41809,35	70,90%	99,48%	98,51%	87,40%	100,00%
34.00_914.8309n	GL03010058	TG(56:2)	932,8644	1	34,00362	1,517067	0,005636	1,488698	MMC	Pooled	x	GL030100	TG(56:2)	M+NH4, M	C59H114N	58,3	2059,697	2876,071	2700,193	2301,994	3066,266	3066,266	67,17%	93,80%	88,06%	75,07%	100,00%
36.98_942.8613n	GL03010059	TG(58:2)	965,8505	1	36,98078	1,435517	0,008945	1,99308	MMC	Pooled		GL030100	TG(58:2)	M+NH4, M	C61H114	47,2	510,4226	767,0819	723,5659	562,3249	1017,313	1017,313	50,17%	75,40%	71,13%	55,28%	100,00%
24.54_744.6273n	GL03010061	TG(44:3)	762,6611	1	24,544	0,426217	0,000183	1,322055	MMC	Folch	x	GL030100	TG(44:3)	M+NH4, M	C47H84O	57,3	462,9498	394,1425	452,6087	430,1828	521,078	521,078	88,84%	75,64%	86,86%	82,56%	100,00%
25.43_772.6586n	GL03010062	TG(46:3)	790,6924	1	25,43293	0,531733	0,557328	1,163513	Bligh	Pooled	x	GL030100	TG(46:3)	M+NH4, M	C49H92N	58,3	875,9269	986,545	919,8428	1019,152	996,5817	1019,152	85,95%	96,80%	90,26%	100,00%	97,79%

26.40_800.6899n	GL03010064	TG(48:3)	818,7237	1	26,40122	1,058933	0,050025	1,120769	MMC	Pooled		GL030100	TG(48:3)	M+NH4, M	C51H96N	49,2	1740,888	1889,705	1842,379	1885,482	1951,133	1951,133	89,22%	96,85%	94,43%	96,64%	100,00%
26.81_814.7058n	GL03010065	TG(49:3)	832,7396	1	26,8077	0,857767	0,019196	1,151719	MMC	Pooled	x	GL030100	TG(49:3)	M+NH4, M	C52H98N	51,1	226,715	254,2857	259,2818	250,1805	261,112	261,112	86,83%	97,39%	99,30%	95,81%	100,00%
27.39_828.7215n	GL03010066	TG(50:3)	846,7554	1	27,39055	1,615017	0,003454	1,105252	MMC	Pooled	x	GL030100	TG(50:3)	M+H, M+N	C53H100N	53,8	11521,36	12653,31	12638,41	12439,56	12734	12734	90,48%	99,37%	99,25%	97,69%	100,00%
27.96_842.7370n	GL03010067	TG(51:3)	860,7709	1	27,95852	0,917583	0,007732	1,125169	MMC	Pooled		GL030100	TG(51:3)	M+NH4, M	C54H102N	48,3	1153,909	1291,128	1291,354	1284,876	1298,342	1298,342	88,88%	99,44%	99,46%	98,96%	100,00%
28.61_856.7531n	GL03010068	TG(52:3)	874,7869	1	28,6147	2,3752	0,0322	1,129341	Folch	Pooled	x	GL030100	TG(52:3)	M+H-H2O	C55H104N	63,7	41010,8	46315,17	45402,98	45729,54	45946,25	46315,17	88,55%	100,00%	98,03%	98,74%	99,20%
29.38_870.7686n	GL03010069	TG(53:3)	888,8024	1	29,37607	0,967783	0,018883	1,118528	MTBE	Pooled	x	GL030100	TG(53:3)	M+NH4, M	C56H106N	50,5	1976,204	2209,054	2210,44	2132,735	2196,776	2210,44	89,40%	99,94%	100,00%	96,48%	99,38%
30.04_884.7836n	GL03010070	TG(54:3)	902,818	1	30,03732	0,998133	0,133031	1,148556	Bligh	Pooled	x	GL030100	TG(54:3)	M+H, M+N	C57H108N	63,1	50984,38	55999	53512,59	58558,4	58283,04	58558,4	87,07%	95,63%	91,38%	100,00%	99,53%
30.90_898.7992n	GL03010071	TG(55:3)	916,833	1	30,90212	1,394483	0,000231	1,328315	MMC	Pooled	x	GL030100	TG(55:3)	M+NH4, M	C58H106	51,4	556,8471	735,2854	728,653	630,5104	739,6681	739,6681	75,28%	99,41%	98,51%	85,24%	100,00%
31.91_930.8486m/z	GL03010072	TG(56:3)	930,8486	1	31,90868	1,6076	0,000316	1,491592	MMC	Pooled	x	GL030100	TG(56:3)	M+NH4	C59H112N	52,6	2663,283	3824,519	3736,189	3242,967	3972,531	3972,531	67,04%	96,27%	94,05%	81,63%	100,00%
34.34_940.8461n	GL03010073	TG(58:3)	958,8799	1	34,34002	0,941467	0,013414	2,033309	MMC	Pooled	x	GL030100	TG(58:3)	M+NH4, M	C61H112	51,8	237,196	365,1926	330,0538	266,4879	482,2927	482,2927	49,18%	75,72%	68,43%	55,25%	100,00%
26.60_826.7053n	GL03010077	TG(50:4)	844,7391	1	26,60302	1,30345	0,00228	1,165116	Bligh	Pooled	x	GL030100	TG(50:4)	M+H, M+N	C53H98N	58,2	4002,454	4539,882	4422,014	4663,322	4562,031	4663,322	85,83%	97,35%	94,83%	100,00%	97,83%
27.67_854.7367n	GL03010080	TG(52:4)	872,7706	1	27,67018	1,913367	0,001844	1,113044	MMC	Pooled		GL030100	TG(52:4)	M+H, M+N	C55H102N	47,2	23176,07	25730,38	25592,01	25365,19	25795,99	25795,99	89,84%	99,75%	99,21%	98,33%	100,00%
28.13_868.7517n	GL03010081	TG(53:4)	886,7856	1	28,12928	1,911717	0,00025	1,219909	MMC	Pooled		GL030100	TG(53:4)	M+NH4, M	C56H104N	49,5	540,8441	631,1626	636,7819	599,3604	659,7807	659,7807	81,97%	95,66%	96,51%	90,84%	100,00%
28.87_900.8024m/z	GL03010082	TG(54:4)	900,8024	1	28,86603	0,842717	0,024368	1,162928	Folch	Pooled	x	GL030100	TG(54:4)	M+NH4	C57H106N	63,4	24344,65	28311,07	27123,52	26788,66	27501,58	28311,07	85,99%	100,00%	95,81%	94,62%	97,14%
32.44_938.8303n	GL03010086	TG(58:4)	956,8641	1	32,43985	0,898683	0,030181	1,487491	MMC	Pooled	x	GL030100	TG(58:4)	M+NH4, M	C61H110	55,5	250,0276	350,7535	332,6111	261,8422	371,9138	371,9138	67,23%	94,31%	89,43%	70,40%	100,00%
26.95_852.7208n	GL03010089	TG(52:5)	870,7551	1	26,95072	1,033133	0,004913	1,145794	MMC	Pooled		GL030100	TG(52:5)	M+H, M+N	C55H100N	48,8	5282,3	5950,016	5994,474	5756,163	6052,425	6052,425	87,28%	98,31%	99,04%	95,11%	100,00%
27.35_884.7702m/z	GL03010092	TG(53:5)	884,7702	1	27,34758	1,00365	0,000688	1,165563	MMC	Pooled	x	GL030100	TG(53:5)	M+NH4	C56H102N	51,1	219,3209	249,9701	247,8244	241,8393	255,6323	255,6323	85,80%	97,79%	96,95%	94,60%	100,00%
27.86_880.7520n	GL03010093	TG(54:5)	898,7858	1	27,86165	1,2654	0,000771	1,161277	MMC	Pooled	x	GL030100	TG(54:5)	M+H, M+N	C57H104N	54	9319,41	10277,18	10252,93	10251,06	10822,42	10822,42	86,11%	94,96%	94,74%	94,72%	100,00%
28.40_880.7528n	GL03010094	TG(54:5)	898,7866	1	28,40127	0,692867	0,169111	1,101635	Bligh	Pooled		GL030100	TG(54:5)	M+NH4, M	C57H104N	49,7	3001,696	3286,909	3187,435	3306,772	3212,643	3306,772	90,77%	99,40%	96,39%	100,00%	97,15%
29.09_912.8025m/z	GL03010096	TG(55:5)	912,8025	1	29,08755	0,272283	0,035305	1,212842	Folch	Pooled	x	GL030100	TG(55:5)	M+NH4	C58H106N	56,3	121,1447	146,9294	138,94	133,0567	142,477	146,9294	82,45%	100,00%	94,56%	90,56%	96,97%
29.43_926.8175m/z	GL03010098	TG(56:5)	926,8175	1	29,42977	0,659533	0,066038	1,126524	MTBE	Pooled	x	GL030100	TG(56:5)	M+NH4	C59H108N	53,7	1316,952	1481,154	1483,579	1418,296	1443,393	1483,579	88,77%	99,84%	100,00%	95,60%	97,29%
29.89_908.7840n	GL03010099	TG(56:5)	926,8178	1	29,89308	0,807133	0,044965	1,180634	Folch	Pooled		GL030100	TG(56:5)	M+NH4, M	C59H108N	50	2516,545	2971,119	2833,381	2920,978	2866,574	2971,119	84,70%	100,00%	95,36%	98,31%	96,48%
31.38_954.8484m/z	GL03010100	TG(58:5)	954,8484	1	31,38428	0,653	4,64E-05	1,528257	Folch	Pooled	x	GL030101	TG(58:5)	M+NH4	C61H112N	51,5	226,1467	345,6103	315,5703	256,5798	331,6719	345,6103	65,43%	100,00%	91,31%	74,24%	95,97%
27.13_878.7355n	GL03010102	TG(54:6)	896,7693	1	27,13363	1,300567	0,000592	1,205218	MMC	Pooled		GL030101	TG(54:6)	M+NH4, M	C57H102N	48	4620,579	5096,722	4970,635	4924,371	5568,805	5568,805	82,97%	91,52%	89,26%	88,43%	100,00%
27.48_878.7370n	GL03010103	TG(54:6)	896,7708	1	27,48368	0,728333	0,000112	1,151542	MTBE	Pooled		GL030101	TG(54:6)	M+NH4, M	C57H102N	45,8	3269,648	3751,283	3765,138	3655,176	3664,171	3765,138	86,84%	99,63%	100,00%	97,08%	97,32%
28.54_924.8022m/z	GL03010106	TG(56:6)	924,8022	1	28,54118	1,24615	0,008305	1,170523	Folch	Pooled	x	GL030101	TG(56:6)	M+NH4	C59H106N	50,6	3468,998	4060,541	3875,29	3897,575	3878,452	4060,541	85,43%	100,00%	95,44%	95,99%	95,52%
28.76_920.7838n	GL03010110	TG(57:6)	938,8177	1	28,75682	0,889017	0,00266	1,156007	Bligh	Pooled		GL030101	TG(57:6)	M+NH4, M	C60H108N	66,3	994,0284	1136,584	1031,818	1149,104	1132,963	1149,104	86,50%	98,91%	89,79%	100,00%	98,60%
30.15_952.8336m/z	GL03010112	TG(58:6)	952,8336	1	30,14747	1,263167	0,003765	1,269456	Folch	Pooled		GL030101	TG(58:6)	M+NH4	C61H110N	61,9	684,9264	869,4839	804,4898	818,1995	828,7144	869,4839	78,77%	100,00%	92,52%	94,10%	95,31%
26.43_876.7204n	GL03010114	TG(54:7)	894,7542	1	26,43242	0,7255	0,080379	1,150951	MMC	Pooled		GL030101	TG(54:7)	M+H, M+N	C57H100N	49,3	1225,384	1354,335	1335,826	1383,629	1410,357	1410,357	86,88%	96,03%	94,72%	98,10%	100,00%
26.78_876.7211n	GL03010116	TG(54:7)	894,7549	1	26,78033	0,601333	0,011304	1,221893	Bligh	Pooled	x	GL030101	TG(54:7)	M+NH4, M	C57H100N	53,2	1318,222	1529,864	1522,96	1610,726	1542,483	1610,726	81,84%	94,98%	94,55%	100,00%	95,76%
27.63_922.7863m/z	GL03010118	TG(56:7)	922,7863	1	27,62717	0,373917	0,036716	1,132859	Folch	Pooled		GL030101	TG(56:7)	M+NH4	C59H104N	46,9	1993,268	2258,091	2243,228	2196,308	2222,44	2258,091	88,27%	100,00%	99,34%	97,26%	98,42%
30.20_978.8486m/z	GL03010124	TG(60:7)	978,8486	1	30,20258	0,54905	0,045148	1,515878	MMC	Pooled		GL030101	TG(60:7)	M+NH4	C63H108	46	43,72546	64,30655	60,84975	48,91156	66,28247	66,28247	65,97%	97,02%	91,80%	73,79%	100,00%
25.83_913.6679m/z	GL03010125	TG(54:8)	913,6679	1	25,8272	0,1956	0,365142	1,473932	MMC	Folch	x	GL030101	TG(54:8)	M+K	C57H98N	51,8	6,613079	5,976799	6,982268	6,358079	8,809396	8,809396	75,07%	67,85%	79,26%	72,17%	100,00%
25.84_874.7058n	GL03010125	TG(54:8)	892,7396	1	25,84273	0,26925	0,550597	1,187964	MMC	Pooled	x	GL030101	TG(54:8)	M+NH4, M	C57H98N	52,6	168,3353	178,7282	176,1864	184,5143	199,9762	199,9762	84,18%	89,37%	88,10%	92,27%	100,00%
26.25_892.7381m/z	GL03010127	TG(54:8)	892,7381	1	26,24663	0,547083	0,067632	1,156296	MTBE	Pooled		GL030101	TG(54:8)	M+NH4	C57H98N	49,1	280,5158	313,6875	324,3593	324,0295	320,5492	324,3593	86,48%	96,71%	100,00%	99,90%	98,83%
27.17_920.7700m/z	GL03010129	TG(56:8)	920,77	1	27,16908	1,811283	0,028186	1,123367	MMC	Pooled		GL030101	TG(56:8)	M+NH4	C59H102N	47,9	2936,158	3255,128	3276,232	3110,764	3298,384	3298,384	89,02%	98,69%	99,33%	94,31%	100,00%
28.27_948.8018m/z	GL03010131	TG(58:8)	948,8018	1	28,27063	1,107967	0,002964	1,150614	MMC	Pooled	x	GL030101	TG(58:8)	M+NH4	C61H106N	57,9	710,6658	817,0718	817,3417	799,9939	817,7018	817,7018	86,91%	99,92%	99,96%	97,83%	100,00%
29.13_976.8321m/z	GL03010133	TG(60:8)	976,8321	1	29,12667	0,558017	0,045747	1,281283	Folch	Pooled	x	GL030101	TG(60:8)	M+NH4	C63H106	50,4	42,99199	55,08492	54,0451	43,11736	54,52783	55,08492	78,05%	100,00%	98,11%	78,27%	98,99%
27.44_928.7525n	GL03010135	TG(58:9)	946,7863	1	27,44428	1,437033	0,010929	1,114602	MMC	Pooled		GL030101	TG(58:9)	M+NH4, M	C61H104N	49,5	1023,53	1135,411	1132,913	1105,442	1140,828	1140,828	89,72%	99,53%	99,31%	96,90%	100,00%
28.76_974.8171m/z	GL03010138	TG(60:9)	974,8171	1	28,75682	0,324533	0,323771	1,18612	MTBE	Pooled		GL030101	TG(60:9)	M+NH4	C63H104	45,6	18,33466	20,73959	21,7471	19,0635	20,99883	21,7471	84,31%	95,37%	100,00%	87,66%	96,56%
26.64_926.7357n	GL03010139	TG(58:10)	944,7695	1	26,64078	0,99275	0,00175	1,184027	Bligh	Pooled	x	GL030101	TG(58:10)	M+NH4, M	C61H102N	51,8	546,0689	634,2996	626,3688	646,5601	627,6732	646,5601	84,46%	98,10%	96,88%	100,00%	97,08%
27.08_926.7365n	GL03010140	TG(58:10)	944,7703	1	27,07583	0,226317	0,075177	1,136205	MTBE	Pooled		GL030101	TG(58:10)	M+NH4, M	C61H102N	47,1	88,70218	98,1226	100,7839	97,53213	97,28201	100,7839	88,01%	97,36%	100,00%	96,77%	96,53%
27.58_972.8012m/z	GL03010141	TG(60:10)	972,8012	1	27,5843	0,384233	0,021304	1,204576	MTBE	Pooled	x	GL030101	TG(60:10)	M+NH4	C63H102	51,5	46,54442	54,73281	56,06627	54,67094	54,38876	56,06627	83,02%	97,62%	100,00%	97,51%	97,01%
28.13_972.8018m/z	GL03010142	TG(60:10)	972,8018	1	28,12928	0,5398	0,004288	1,313738	MTBE	Pooled	x	GL030101	TG(60:10)	M+NH4	C63H102	53,6	54,73381	69,59668	71,90591	63,4722	71,39539	71,90591	76,12%	96,79%	100,00%	88,27%	99,29%
27.17_952.7523n	GL03010143	TG(60:11)	970,7861	1	27,16908	0,498017	0,196069	1,127802	MTBE	Pooled	x	GL030101	TG(60:11)	M+NH4, M	C63H104N	54,6	174,2389	195,2337	196,5071	184,5155	196,3087	196,5071	88,67%	99,35%	100,00%	93,90%	99,90%
26.51_968.7701m/z	GL03010146	TG(60:12)	968,7701	1	26,51333	0,483567	0,209757	1,157503	Folch	Pooled		GL030101	TG(60:12)	M+NH4	C63H98O	48,6	86,0977	99,65839	94,64583	91,47578	96,07236	99,65839	86,39%	100,00%	94,97%	91,79%	96,40%
26.80_950.7353n	GL03010147	TG(60:12)	968,7691	1	26,79985	0,2827	0,095697	1,27888	Bligh	Pooled	x	GL030101	TG(60:12)	M+NH4, M	C63H98O	51,3	58,47735	72,08762	72,46579	74,78554	69,04443	74,78554	78,19%	96,39%	96,90%	100,00%	92,32%
26.31_992.7704m/z	GL03010148	TG(62:14)	992,7704	1	26,30907	0,355567	0,342908	1,313498	Folch	Bligh		GL030101	TG(62:14)	M+NH4	C65H98O	48,3	15,32766	16,93272	15,91314	12,89132	16,7421	16,93272	90,52%	100,00%	93,98%	76,13%	98,87%
26.31_850.7059n	14711351	TG(52:6)	868,7391	1	26,30907	0,88735	0,01199	1,156075	Bligh	Pooled		14711351	TG(52:6)	M+NH4, M	C55H94O	44,1	1525,798	1717,836	1741,611	1763,936	1692,362	1763,936	86,50%	97,39%	98,73%	100,00%	95,94%
30.21_914.8173m/z	14711632	TG(55:4)	914,8173	1	30,21057	0,27695	0,141582	1,517972	MMC	Pooled		14711632	TG(55:4)	M+NH4	C58H104	36,1	21,59038	24,14547	26,10675	22,39253	32,77359	32,77359	65,88%	73,67%	79,66%	68,32%	100,00%
30.75_910.7992n	14711794	TG(56:4)	928,8331	1	30,74658	1,1585	0,000209	1,460025	Folch	Pooled		14711794	TG(56:4)	M+NH4, M	C59H106	43,7	1591,341	2323,398	2149,708	1814,384	2051,601	2323,398	68,49%	100,00%	92,52%	78,09%	88,30%
26.27_900.7203n	14712035	TG(56:9)	918,7541	1	26,27023	0,83435	0,015303	1,129294	MMC	Pooled		14712035	TG(56:9)	M+NH4, M	C59H96O	40,7	676,7211	758,0711	747,8768	748,7938	764,217	764,217	88,55%	99,20%	97,86%	97,98%	100,00%
31.76_954.8483m/z	14712376	TG(58:5)	954,8483	1	31,75722	0,583183	0,024937	1,64046	MMC	Pooled		14712376	TG(58:5)	M+NH4	C61H108	36	111,6944	159,3876	165,2764	140,0128	183,2303	183,2303	60,96%	86,99%	90,20%	76,41%	100,00%
26.09_924.7205n	14712778	TG(58:11)	942,7544	1	26,09242	0,667	0,003458	1,182175	MTBE	Pooled		14712778	TG(58:11)	M+NH4, M	C61H96O	39,4	163,9806	184,8313	193,8538	180,7455	187,8774	193,8538	84,59%	95,35%	100,00%	93,24%	96,92%
25.92_948.7208n	14713067	TG(60:13)	966,7546	1	25,91502	0,308017	0,332876	1,293962	MMC	Bligh		14713067	TG(60:13)	M+NH4, M	C63H96O	37,3	20,68959	21,25738	22,25069	19,61347	25,37908	25,37908	81,52%	83,76%	87,67%	77,28%	100,00%
26.99_994.7863m/z	14713558	TG(62:13)	994,7863	1	26,99365	0,325783	0,140017	1,217351	MMC	Pooled		14713558	TG(62:13)	M+NH4	C65H100	39,8	20,48243	22,56576	23,28875	21,56231	24,93431	24,93431	82,15%	90,50%	93,40%	86,48%	100,00%
25.14_770.6429n	135638342	TG(46:4)	793,6318	1	25,13532	0,21325	0,35841	1,184638	MMC	MTBE		1,36E+08	TG(46:4)	M+NH4, M	C49H86O	35,5	35,075	35,49378	33,6526	35,24864	39,86616	39,86616	87,98%	89,03%	84,41%	88,42%	100,00%
24.15_768.6269n	135638509	TG(46:5)	791,6163	1	24,148	0,224617	1,84E-06	1,642649	MMC	Pooled		1,36E+08	TG(46:5)	M+NH4, M	C49H84O	37,5	46,81406	48,0014	52,44148	52,8066	76,89907	76,89907	60,88%	62,42%	68,20%	68,67%	100,00%
28.03_804.7215n	135638564	TG(48:1)	822,7553	1	28,03297	0,730667	0,00053	1,190399	Folch	Pooled		1,36E+08	TG(48:1)	M+NH4, M	C51H96O	41	5849,824	6963,624	6408,505	6301,715	6579,34	6963,624	84,01%	100,00%	92,03%	90,49%	94,48%
23.91_742.6109n	135638935	TG(44:4)	765,6005	1	23,91107	0,318117	4,26E-07	1,570219	MMC	Folch		1,36E+08	TG(44:4)	M+NH4, M	C47H82O	37,4	164,1456	138,9342	153,948	151,1541	218,1572	218,1572	75,24%	63,69%	70,57%	69,29%	100,00%
24.81_770.6429n	135639016	TG(46:4)	793,6324	1	24,80748	0,36255	0,000823	1,38383	MMC	Pooled		1,36E+08	TG(46:4)	M+NH4, M	C49H86O	38,8	398,4274	460,662	412,4551	427,1169	551,3559	551,3559	72,26%	83,55%	74,81%	77,47%	100,00%
25.09_814.6918m/z	135639169	TG(48:5)	814,6918	1	25,0941	0,198583	0,085744	1,282191	MMC	Bligh		1,36E+08	TG(48:5)	M+NH4	C51H88O	39,4	38,03763	38,47186	36,48581	34,07658	43,69269	43,69269	87,06%	88,05%	83,51%	77,99%	100,00%
25.12_820.6582n	135639227	TG(50:9)	843,6475	1	25,11985	0,175617	0,240776	1,370426	MMC	Bligh		1,36E+08	TG(50:9)	M+NH4, M	C53H88O	39,7	21,99824	23,8971	21,83189	19,58046	26,83357	26,83357	81,98%	89,06%	81,36%	72,97%	100,00%
25.93_778.6916m/z	135639368	TG(45:2)	778,6916	1	25,93472	0,153783	0,135474	1,766228	MMC	Bligh		1,36E+08	TG(45:2)	M+NH4	C48H88O	34,9	5,192637	4,242092	5,322049	3,76655	6,652585	6,652585	78,05%	63,77%	80,00%	56,62%	100,00%
25.29_796.6581n	135639462	TG(48:5)	819,6477	1	25,28772	0,396917	0,169322	1,188375	MMC	Bligh		1,36E+08	TG(48:5)	M+NH4, M	C51H88O	39,5	80,33825	89,32769	84,40204	78,79412	93,63694	93,63694	85,80%	95,40%	90,14%	84,15%	100,00%
24.74_794.6426n	135639843	TG(48:6)	817,6321	1	24,74227	0,2029	0,043703	1,373511	MMC	Bligh		1,36E+08	TG(48:6)	M+NH4, M	C51H86O	39	34,04107	32,00173	33,44956	29,88639	41,04926	41,04926	82,93%	77,96%	81,49%	72,81%	100,00%
26.60_788.6897n	135639934	TG(47:2)	806,7235	1	26,59522	0,64165	0,023494	1,246249	MMC	Pooled		1,36E+08	TG(47:2)	M+NH4, M	C50H92O	39,2	110,0603	125,8403	126,1755	123,5613	137,1626	137,1626	80,24%	91,75%	91,99%	90,08%	100,00%
26.25_830.7237m/z	135639965	TG(49:4)	830,7237	1	26,24663	0,5912	0,361052	1,381378	MMC	Pooled		1,36E+08	TG(49:4)	M+NH4	C52H92O	37,7	25,13863	29,02465	32,56386	25,48669	34,72596	34,72596	72,39%	83,58%	93,77%	73,39%	100,00%
25.52_822.6738n	135640070	TG(50:6)	840,7076	1	25,52402	0,753883	0,145686	1,180875	Folch	Pooled		1,36E+08	TG(50:6)	M+NH4, M	C53H90O	39,9	213,7507	252,4129	233,4869	233,287	234,7393	252,4129	84,68%	100,00%	92,50%	92,42%	93,00%
26.68_856.7389m/z	135640513	TG(51:5)	856,7389	1	26,67585	0,60325	0,114884	1,181377	MMC	Pooled		1,36E+08	TG(51:5)	M+NH4	C54H94O	41,2	84,79325	96,32838	97,34434	91,61727	100,1728	100,1728	84,65%	96,16%	97,18%	91,46%	100,00%
30.08_838.7865m/z	135640695	TG(49:0)	838,7865	1	30,0839	0,681317	0,030835	1,410468	Bligh	MTBE		1,36E+08	TG(49:0)	M+NH4	C52H100	39,8	95,76186	86,99205	71,46151	100,7942	88,21489	100,7942	95,01%	86,31%	70,90%	100,00%	87,52%
30.59_888.8015m/z	135640892	TG(53:3)	888,8015	1	30,58713	0,539767	0,025163	1,704634	Folch	Pooled		1,36E+08	TG(53:3)	M+NH4	C56H102	36,3	45,74497	77,9784	74,47426	58,64941	75,34307	77,9784	58,66%	100,00%	95,51%	75,21%	96,62%
28.03_904.7526n	135640950	TG(56:7)	922,7864	1	28,03297	0,967283	0,006158	1,152469	Folch	Pooled	x	1,36E+08	TG(56:7)	M+NH4, M	C59H100	41,9	2874,23	3312,46	3256,055	3274,76	3245,748	3312,46	86,77%	100,00%	98,30%	98,86%	97,99%
27.99_910.7861m/z	135640977	TG(55:6)	910,7861	1	27,98987	0,687417	0,030646	1,165344	MMC	Pooled		1,36E+08	TG(55:6)	M+NH4	C58H100	42	102,1972	114,5935	116,2956	109,9133	119,0948	119,0948	85,81%	96,22%	97,65%	92,29%	100,00%
28.13_936.8021m/z	135641009	TG(57:7)	936,8021	1	28,12928	0,324717	0,093391	1,567068	MMC	Bligh		1,36E+08	TG(57:7)	M+NH4	C60H102	34,4	7,483703	8,79956	10,95305	7,257541	11,37306	11,37306	65,80%	77,37%	96,31%	63,81%	100,00%
28.73_936.8018m/z	135641009	TG(57:7)	936,8018	1	28,72552	0,251333	0,078119	1,327969	MTBE	Pooled		1,36E+08	TG(57:7)	M+NH4	C60H102	35,5	9,876228	12,33404	13,11533	10,21903	12,50432	13,11533	75,30%	94,04%	100,00%	77,92%	95,34%
26.00_848.6889n	135641131	TG(52:7)	866,7227	1	26,00407	0,746167	0,090696	1,176663	Folch	Pooled		1,36E+08	TG(52:7)	M+NH4, M	C55H92O	40,4	329,3247	387,5041	360,2966	380,2278	362,7305	387,5041	84,99%	100,00%	92,98%	98,12%	93,61%
26.68_906.7544m/z	135641254	TG(55:8)	906,7544	1	26,67585	0,410417	0,774217	1,168601	Folch	Bligh		1,36E+08	TG(55:8)	M+NH4	C58H96O	38,4	22,70427	25,11356	21,81269	21,49028	22,73298	25,11356	90,41%	100,00%	86,86%	85,57%	90,52%
27.71_934.7861m/z	135641615	TG(57:8)	934,7861	1	27,71327	0,653367	0,2993	1,23171	MTBE	Pooled		1,36E+08	TG(57:8)	M+NH4	C60H100	36	16,20666	18,71165	19,96191	19,00301	19,24021	19,96191	81,19%	93,74%	100,00%	95,20%	96,38%
32.75_900.8146n	135641719	TG(55:2)	923,8037	1	32,74652	0,81005	0,018712	1,504098	MMC	Pooled		1,36E+08	TG(55:2)	M+NH4, M	C58H108	41,4	255,8331	337,7606	352,3582	257,2648	384,7979	384,7979	66,49%	87,78%	91,57%	66,86%	100,00%
30.30_862.7862m/z	TG(16:0/17:1/18:1)	TG(51:2)	862,7862	1	30,30355	0,475667	0,016196	1,182419	MMC	Pooled		TG(16:0/1	TG(51:2)	M+NH4	C54H100	39,2	106,4565	124,9793	122,8003	108,83	125,8762	125,8762	84,57%	99,29%	97,56%	86,46%	100,00%
36.07_890.8302n	TG(16:0/18:0/20:0)	TG(54:0)	913,8198	1	36,0667	0,39495	0,198496	2,20009	MMC	Folch		TG(16:0/1	TG(54:0)	M+NH4, M	C57H110	37,9	87,42437	62,69199	75,12856	102,7215	137,928	137,928	63,38%	45,45%	54,47%	74,47%	100,00%
25.98_842.7237m/z	TG(16:1/16:1/18:3)	TG(50:5)	842,7237	1	25,9812	0,795083	0,026705	1,177847	Folch	Pooled		TG(16:1/1	TG(50:5)	M+NH4	C53H92O	40,4	519,5669	611,9702	585,3854	601,863	587,3054	611,9702	84,90%	100,00%	95,66%	98,35%	95,97%
27.13_840.7216n	TG(16:1/17:1/18:2)	TG(51:4)	858,7554	1	27,12563	0,959	0,008067	1,128688	MMC	Pooled		TG(16:1/1	TG(51:4)	M+NH4, 2	C54H96O	44,2	477,9971	533,5022	522,9358	518,176	539,5095	539,5095	88,60%	98,89%	96,93%	96,05%	100,00%
28.83_912.8024m/z	TG(17:0/18:1/20:4)	TG(55:5)	912,8024	1	28,82667	0,628233	0,36792	1,111763	Bligh	Pooled		TG(17:0/1	TG(55:5)	M+NH4	C58H102	42,8	70,51683	78,15255	76,62876	78,39799	76,51182	78,39799	89,95%	99,69%	97,74%	100,00%	97,59%
27.16_908.7694m/z	TG(17:0/18:2/20:5)	TG(55:7)	908,7694	1	27,16113	0,353933	0,076381	1,186277	MMC	Pooled		TG(17:0/1	TG(55:7)	M+NH4	C58H98O	38,9	59,05477	67,75966	65,50971	60,18467	70,05531	70,05531	84,30%	96,72%	93,51%	85,91%	100,00%
29.61_914.8177m/z	TG(17:1/18:1/20:2)	TG(55:4)	914,8177	1	29,61418	0,425983	0,023314	1,205218	MMC	Pooled		TG(17:1/1	TG(55:4)	M+NH4	C58H104	40,7	39,94483	46,25449	47,65923	45,57006	48,14224	48,14224	82,97%	96,08%	99,00%	94,66%	100,00%
26.90_882.7547m/z	TG(17:2/18:2/18:2)	TG(53:6)	882,7547	1	26,89738	0,916533	0,03338	1,211337	MMC	Pooled		TG(17:2/1	TG(53:6)	M+NH4	C56H96O	41,2	97,41542	114,6473	115,3418	100,3369	118,0029	118,0029	82,55%	97,16%	97,74%	85,03%	100,00%
36.53_916.8461n	TG(18:0/18:1/20:0)	TG(56:1)	939,8353	1	36,5267	1,202533	0,028946	1,95462	MMC	Bligh		TG(18:0/1	TG(56:1)	M+NH4, M	C59H112	37,7	299,2071	307,1622	287,8795	232,177	453,8177	453,8177	65,93%	67,68%	63,44%	51,16%	100,00%
30.44_928.8328m/z	TG(18:1/18:1/20:2)	TG(56:4)	928,8328	1	30,43932	0,4473	0,007356	1,345821	Folch	Pooled		TG(18:1/1	TG(56:4)	M+NH4	C59H106	45,4	2009,734	2704,741	2433,606	2374,413	2702,882	2704,741	74,30%	100,00%	89,98%	87,79%	99,93%
29.84_914.8181m/z	TG(18:2/18:2/19:0)	TG(55:4)	914,8181	1	29,84198	0,340833	0,284116	1,280506	MTBE	Pooled		TG(18:2/1	TG(55:4)	M+NH4	C58H104	36,8	29,37908	37,18932	37,6201	35,10764	35,60729	37,6201	78,09%	98,85%	100,00%	93,32%	94,65%
25.84_898.7054n	TG(18:2/18:3/20:5)	TG(55:10)	916,7393	1	25,84273	0,387783	0,655644	1,123099	MTBE	Pooled		TG(18:2/1	TG(55:10)	M+NH4, M	C59H94O	36,6	86,90812	90,50098	97,60646	90,9016	96,83119	97,60646	89,04%	92,72%	100,00%	93,13%	99,21%
25.57_922.7040n	TG(18:2/20:5/20:5)	TG(58:12)	940,7379	1	25,57412	0,195433	0,036202	1,651024	MMC	Pooled		TG(18:2/2	TG(58:12)	M+NH4, M	C61H94O	37	19,06046	24,88708	23,82265	22,22659	31,46929	31,46929	60,57%	79,08%	75,70%	70,63%	100,00%
20.79_568.5073n	GL03010149	DG(32:0)	551,504	1	20,78577	0,292367	5,64E-09	1,417026	MMC	Folch	x	GL030101	DG(32:0)	M+H-H2O	C35H68O	57,3	182,3108	164,011	177,879	205,7903	232,4078	232,4078	78,44%	70,57%	76,54%	88,55%	100,00%
19.81_566.4918n	GL03010150	DG(32:1)	549,4885	1	19,80932	0,3436	1,22E-15	1,63476	MMC	Folch	x	GL030101	DG(32:1)	M+H-H2O	C35H70N	51,7	102,7779	80,28922	89,54408	106,5094	131,2536	131,2536	78,30%	61,17%	68,22%	81,15%	100,00%
21.05_594.5233n	GL03010151	DG(34:1)	577,52	1	21,04648	0,48915	7,77E-16	1,479794	MMC	Folch		GL030101	DG(34:1)	M+H-H2O	C37H74N	61,4	1865,409	1530,697	1653,936	1940,032	2265,117	2265,117	82,35%	67,58%	73,02%	85,65%	100,00%
22.27_622.5543n	GL03010153	DG(36:1)	645,5435	1	22,2712	0,283	2,91E-05	1,334489	MMC	Folch	x	GL030101	DG(36:1)	M+H-H2O	C39H78N	51,5	194,1879	177,8476	187,1819	210,4426	237,3356	237,3356	81,82%	74,94%	78,87%	88,67%	100,00%
20.23_592.5075n	GL03010155	DG(34:2)	575,5042	1	20,22685	0,51615	0	1,480213	MMC	Folch	x	GL030101	DG(34:2)	M+H-H2O	C37H72N	53,1	429,6736	369,9347	385,6342	477,6759	547,5821	547,5821	78,47%	67,56%	70,42%	87,23%	100,00%
21.31_620.5390n	GL03010157	DG(36:2)	603,5357	1	21,31205	0,54065	1,68E-05	1,298361	MMC	Folch	x	GL030101	DG(36:2)	M+H-H2O	C39H76N	58,3	714,3691	686,744	691,9642	806,5474	891,6414	891,6414	80,12%	77,02%	77,61%	90,46%	100,00%
22.44_648.5701n	GL03010159	DG(38:2)	631,5669	1	22,44057	0,524383	0,06418	1,111939	MTBE	Folch	x	GL030101	DG(38:2)	M+H-H2O	C41H76O	54,3	2166,221	2143,705	2383,67	2197,463	2172,274	2383,67	90,88%	89,93%	100,00%	92,19%	91,13%
20.43_618.5234n	GL03010161	DG(36:3)	601,5201	1	20,42967	0,600317	2,56E-09	1,343067	MMC	Folch		GL030101	DG(36:3)	M+H-H2O	C39H70O	49,4	458,601	405,8059	424,4654	491,0666	545,0247	545,0247	84,14%	74,46%	77,88%	90,10%	100,00%
19.75_634.5412m/z	14710916	DG(36:4)	634,5412	1	19,75462	0,350433	1,81E-06	1,328457	Folch	Pooled		14710916	DG(36:4)	M+NH4	C39H68O	37,8	21,25614	28,23786	23,5765	24,71174	23,97556	28,23786	75,28%	100,00%	83,49%	87,51%	84,91%
20.11_616.5067n	14710921	DG(36:4)	617,514	1	20,1112	0,251183	0,000912	1,734125	MMC	Folch		14710921	DG(36:4)	M+H-H2O	C39H68O	36,6	12,99514	11,27501	13,40196	15,79615	19,55228	19,55228	66,46%	57,67%	68,54%	80,79%	100,00%
20.30_642.5231n	GL03010167	DG(38:5)	660,5569	1	20,30497	0,477867	0,000112	1,270263	Bligh	MTBE		GL030101	DG(38:5)	M+H-H2O	C41H74N	49,1	66,35357	66,76586	58,80921	74,70315	74,09214	74,70315	88,82%	89,37%	78,72%	100,00%	99,18%
19.44_658.5418m/z	GL03010168	DG(38:6)	658,5418	1	19,4432	0,334733	2,39E-05	2,353882	Folch	MMC	x	GL030101	DG(38:6)	M+NH4	C41H68O	50,4	3,295107	7,111287	3,938709	4,474308	3,02109	7,111287	46,34%	100,00%	55,39%	62,92%	42,48%
19.49_641.5154m/z	GL03010168	DG(38:6)	641,5154	1	19,48647	0,143167	0,017724	1,958191	MMC	Folch	x	GL030101	DG(38:6)	M+H	C41H68O	52,5	2,921113	2,152574	3,190837	3,659897	4,215152	4,215152	69,30%	51,07%	75,70%	86,83%	100,00%

20.64_686.5725m/z	GL03010169	DG(40:6)	686,5725	1	20,63557	0,251167	0,809062	1,177763	Folch	Bligh	x	GL030101	DG(40:6)	M+NH4	C43H72O!	53,5	4,941098	5,099634	4,541991	4,329931	4,791202	5,099634	96,89%	100,00%	89,07%	84,91%	93,95%
20.23_666.5230n	GL03010170	DG(40:7)	684,5568	1	20,22685	0,317517	0,000212	1,361249	MMC	MTBE	x	GL030101	DG(40:7)	M+H, M+N	C43H70O!	50,6	27,15369	24,49491	24,11288	29,51684	32,82364	32,82364	82,73%	74,63%	73,46%	89,93%	100,00%
16.42_677.5003n	7982877	PC(28:0)	678,5075	1	16,41882	0,254083	0,879424	1,107467	MTBE	Folch		7982877	PC(28:0)	M+H, M+N	C36H72N(	38,8	134,7555	124,239	137,5905	136,5118	126,5447	137,5905	97,94%	90,30%	100,00%	99,22%	91,97%
17.72_705.5320n	GP01010003	PC(30:0)	706,5393	1	17,71985	0,600067	0,234023	1,086383	MTBE	MMC		GP01010(	PC(30:0)	M+H, M+N	C38H76N(	50,6	795,1483	775,0232	833,6569	833,1724	767,3691	833,6569	95,38%	92,97%	100,00%	99,94%	92,05%
18.10_720.5547m/z	GP01010004	PC(31:0)	720,5547	1	18,10312	0,451867	0,117085	1,102872	Bligh	Folch		GP01010(	PC(31:0)	M+H	C39H78N(	49,3	122,8436	115,0597	123,2296	126,8962	116,2013	126,8962	96,81%	90,67%	97,11%	100,00%	91,57%
18.92_733.5633n	GP01010008	PC(32:0)	734,5705	1	18,92257	0,556017	0,118975	1,167324	Bligh	MMC	x	GP01010(	PC(32:0)	M+H, M+N	C40H80N(	62,3	4257,394	4227,943	4255,92	4499,966	3854,941	4499,966	94,61%	93,95%	94,58%	100,00%	85,67%
20.16_762.6014m/z	GP01010011	PC(34:0)	762,6014	1	20,16463	0,59005	0,032016	1,192615	Bligh	MMC	x	GP01010(	PC(34:0)	M+H	C42H84N(	74,4	980,1062	948,1363	918,1545	1067,185	894,8273	1067,185	91,84%	88,84%	86,04%	100,00%	83,85%
20.57_776.6178m/z	GP01010012	PC(35:0)	776,6178	1	20,56958	0,271	0,00405	1,279048	Pooled	MMC		GP01010(	PC(35:0)	M+H	C43H86N(	41,3	14,35419	11,24197	11,50405	14,13435	11,22256	14,35419	100,00%	78,32%	80,14%	98,47%	78,18%
20.79_776.6172m/z	GP01010012	PC(35:0)	776,6172	1	20,78577	0,215867	0,134803	1,359609	Bligh	MTBE		GP01010(	PC(35:0)	M+H	C43H86N(	48,3	6,358852	5,369458	4,922367	6,692494	5,014698	6,692494	95,01%	80,23%	73,55%	100,00%	74,93%
21.35_790.6328m/z	GP01010013	PC(36:0)	790,6328	1	21,35497	0,5862	0,038153	1,327914	Bligh	MTBE	x	GP01010(	PC(36:0)	M+H	C44H88N(	58,4	435,906	434,532	383,6114	509,403	475,4786	509,403	85,57%	85,30%	75,31%	100,00%	93,34%
22.46_817.6567n	GPCho(15:0/23:0)	PC(38:0)	818,664	1	22,46377	0,633667	0,022394	1,372229	Bligh	MMC	x	GPCho(15	PC(38:0)	M+H, M+N	C46H92N(	41,7	3246,71	3019,24	3071,273	3718,023	2709,477	3718,023	87,32%	81,21%	82,60%	100,00%	72,87%
23.57_846.6949m/z	7983144	PC(40:0)	846,6949	1	23,56672	0,21315	0,001667	1,902073	Bligh	Folch		7983144	PC(40:0)	M+H	C48H96N(	34,7	22,75807	12,46569	19,10157	23,71066	13,74042	23,71066	95,98%	52,57%	80,56%	100,00%	57,95%
16.71_703.5160n	GP01010015	PC(30:1)	704,5233	1	16,70878	0,501733	0,017239	1,258619	Pooled	MMC	x	GP01010(	PC(30:1)	M+H, M+N	C38H74N(	53,8	246,2883	218,7085	223,9598	223,1998	195,6813	246,2883	100,00%	88,80%	90,93%	90,63%	79,45%
17.31_717.5312n	GP01010016	PC(31:1)	718,5385	1	17,30612	0,356733	0,009963	1,372285	Bligh	MMC	x	GP01010(	PC(31:1)	M+H, M+N	C39H76N(	56,5	56,66373	63,77731	58,61263	74,11898	54,01136	74,11898	76,45%	86,05%	79,08%	100,00%	72,87%
19.19_759.5785n	GP01010021	PC(34:1)	760,5858	1	19,18982	1,522017	0,303992	1,133071	Bligh	MMC	x	GP01010(	PC(34:1)	M+H, 2M+	C42H82N(	60,5	39704,9	42090,53	40847,85	44546,91	39315,2	44546,91	89,13%	94,49%	91,70%	100,00%	88,26%
19.82_774.6017m/z	GP01010023	PC(35:1)	774,6017	1	19,82493	0,764483	0,043695	1,109491	Bligh	MMC	x	GP01010(	PC(35:1)	M+H	C43H84N(	54	1141,301	1113,219	1166,788	1176,35	1060,261	1176,35	97,02%	94,63%	99,19%	100,00%	90,13%
20.37_787.6102n	GP01010024	PC(36:1)	788,6175	1	20,36612	0,716017	0,025122	1,128668	Bligh	MMC	x	GP01010(	PC(36:1)	M+H, 2M+	C44H86N(	53,6	15260,25	15225,74	15302,22	17102,25	15152,6	17102,25	89,23%	89,03%	89,47%	100,00%	88,60%
20.98_802.6328m/z	GP01010025	PC(37:1)	802,6328	1	20,98462	0,596117	5,57E-05	1,171146	Pooled	MMC		GP01010(	PC(37:1)	M+H	C45H88N(	55,8	113,6725	97,6157	111,9449	111,2217	97,06086	113,6725	100,00%	85,87%	98,48%	97,84%	85,39%
21.52_815.6418n	GP01010026	PC(38:1)	816,6491	1	21,51697	0,4157	0,424073	1,126331	Bligh	MMC	x	GP01010(	PC(38:1)	M+H, M+N	C46H90N(	51,2	349,7405	315,627	325,8294	351,313	311,9093	351,313	99,55%	89,84%	92,75%	100,00%	88,78%
22.70_844.6794m/z	GP01010027	PC(40:1)	844,6794	1	22,70057	0,332617	0,100271	1,337293	Bligh	Folch		GP01010(	PC(40:1)	M+H	C48H94N(	47,6	32,4911	26,7847	32,01756	35,819	29,80471	35,819	90,71%	74,78%	89,39%	100,00%	83,21%
23.78_872.7102m/z	GP01010028	PC(42:1)	872,7102	1	23,78485	0,156583	0,076168	1,934913	Pooled	Folch		GP01010(	PC(42:1)	M+H	C50H98N(	53,5	15,81179	8,171837	13,78603	14,11159	10,67642	15,81179	100,00%	51,68%	87,19%	89,25%	67,52%
17.14_729.5316n	GP01010031	PC(32:2)	730,5388	1	17,1383	0,533117	0,007046	1,143283	Bligh	MMC	x	GP01010(	PC(32:2)	M+H, M+N	C40H76N(	51	1549,25	1453,757	1558,432	1608,057	1406,526	1608,057	96,34%	90,40%	96,91%	100,00%	87,47%
17.83_744.5546m/z	123060906	PC(33:2)	744,5546	1	17,82552	0,736133	0,063658	1,116501	Pooled	MMC		1,23E+08	PC(33:2)	M+H	C41H78N(	49,5	891,4632	800,5596	855,7766	878,2388	798,4436	891,4632	100,00%	89,80%	96,00%	98,52%	89,57%
19.63_786.6020m/z	GP01010039	PC(36:2)	786,602	1	19,63072	0,783867	0,307294	1,126044	Bligh	MMC		GP01010(	PC(36:2)	M+H	C44H84N(	52	31025,52	32309,28	31446,44	34277,81	30440,91	34277,81	90,51%	94,26%	91,74%	100,00%	88,81%
20.23_800.6163m/z	GP01010040	PC(37:2)	800,6163	1	20,22685	0,582983	9,43E-05	1,171446	Bligh	Folch		GP01010(	PC(37:2)	M+H	C45H86N(	53,2	297,8609	274,6019	292,7572	321,6813	280,9387	321,6813	92,60%	85,36%	91,01%	100,00%	87,33%
20.75_814.6327m/z	GP01010041	PC(38:2)	814,6327	1	20,74695	0,786917	0,005846	1,142415	Bligh	MMC	x	GP01010(	PC(38:2)	M+H	C46H88N(	63,2	1718,855	1584,915	1665,846	1793,485	1569,906	1793,485	95,84%	88,37%	92,88%	100,00%	87,53%
21.76_842.6636m/z	GP01010043	PC(40:2)	842,6636	1	21,75913	0,30805	0,732109	1,108916	Pooled	MMC		GP01010(	PC(40:2)	M+H	C48H92N(	53,6	41,98656	39,39746	39,97072	40,11729	37,86272	41,98656	100,00%	93,83%	95,20%	95,55%	90,18%
22.00_842.6637m/z	123060899	PC(40:2)	842,6637	1	21,99643	0,271517	0,030248	1,331935	Pooled	MMC		1,23E+08	PC(40:2)	M+H	C48H92N(	37,1	28,66169	23,6327	24,58238	28,10647	21,51883	28,66169	100,00%	82,45%	85,77%	98,06%	75,08%
22.85_870.6953m/z	160779942	PC(42:2)	870,6953	1	22,84907	0,250233	0,056874	1,650202	Pooled	Folch		1,61E+08	PC(42:2)	M+H	C50H96N(	34,7	18,68314	11,32173	15,00502	16,71585	16,07762	18,68314	100,00%	60,60%	80,31%	89,47%	86,05%
23.12_870.6946m/z	GP01010044	PC(42:2)	870,6946	1	23,12285	0,270717	0,013789	1,473377	Pooled	Folch	x	GP01010(	PC(42:2)	M+H	C50H96N(	55,3	29,26794	19,86453	27,31343	27,82739	23,99451	29,26794	100,00%	67,87%	93,32%	95,08%	81,98%
16.43_728.5230m/z	123060657	PC(32:3)	728,523	1	16,4265	0,273467	0,254832	1,266264	MTBE	Folch		1,23E+08	PC(32:3)	M+H	C40H74N(	37,9	34,00361	29,60111	37,48284	37,07782	31,79356	37,48284	90,72%	78,97%	100,00%	98,92%	84,82%
17.05_742.5401m/z	123060704	PC(33:3)	742,5401	1	17,05445	0,4028	0,001895	1,226216	Pooled	Bligh		1,23E+08	PC(33:3)	M+H	C41H76N(	35,8	39,11334	32,54489	37,7741	31,89759	32,28555	39,11334	100,00%	83,21%	96,58%	81,55%	82,54%
17.38_778.5362m/z	GP01010048	PC(34:3)	778,5362	1	17,38255	0,25115	0,102376	1,376808	Bligh	MTBE		GP01010(	PC(34:3)	M+Na	C42H78N(	56	90,81186	90,93707	74,14393	102,082	76,78384	102,082	88,96%	89,08%	72,63%	100,00%	75,22%
17.67_755.5479n	GP01010049	PC(34:3)	756,5546	1	17,66652	1,009933	0,05886	1,124236	Bligh	MMC	x	GP01010(	PC(34:3)	M+H, M+N	C42H78N(	53,4	4357,743	4134,296	4263,904	4571,278	4066,119	4571,278	95,33%	90,44%	93,28%	100,00%	88,95%
18.78_784.5859m/z	GP01010055	PC(36:3)	784,5859	1	18,78125	1,513583	0,236098	1,127324	Bligh	MMC	x	GP01010(	PC(36:3)	M+H	C44H82N(	58,2	28751,69	29002,04	28562,39	31350,3	27809,48	31350,3	91,71%	92,51%	91,11%	100,00%	88,71%
20.06_811.6099n	GP01010060	PC(38:3)	812,6172	1	20,06025	0,725933	0,006332	1,125285	Bligh	MMC	x	GP01010(	PC(38:3)	M+H, 2M+	C46H86N(	52,5	12631,66	12234,48	12299,22	13660,54	12139,62	13660,54	92,47%	89,56%	90,03%	100,00%	88,87%
20.97_840.6473m/z	123060954	PC(40:3)	840,6473	1	20,96922	0,650967	2,15E-06	1,185528	Bligh	Folch		1,23E+08	PC(40:3)	M+H	C48H90N(	41	110,0576	93,43924	106,8184	110,7749	97,9578	110,7749	99,35%	84,35%	96,43%	100,00%	88,43%
22.13_868.6790m/z	123061284	PC(42:3)	868,679	1	22,13043	0,274767	0,014117	1,332228	Pooled	Bligh		1,23E+08	PC(42:3)	M+H	C50H94N(	36,2	22,93508	19,15347	19,67438	17,21559	17,47893	22,93508	100,00%	83,51%	85,78%	75,06%	76,21%
17.11_753.5310n	GP01010063	PC(34:4)	754,5383	1	17,10748	0,704017	2,97E-05	1,164791	Bligh	MMC	x	GP01010(	PC(34:4)	M+H, M+N	C42H76N(	50,7	588,3394	518,3518	544,889	593,5645	509,589	593,5645	99,12%	87,33%	91,80%	100,00%	85,85%
17.76_768.5544m/z	GP01010065	PC(35:4)	768,5544	1	17,76295	0,654467	0,003213	1,116673	Pooled	Folch		GP01010(	PC(35:4)	M+H	C43H78N(	47,7	286,0463	256,1595	280,4632	284,8191	264,4742	286,0463	100,00%	89,55%	98,05%	99,57%	92,46%
17.87_781.5617n	7983442	PC(36:4)	782,569	1	17,86705	1,052517	0,00247	1,139354	Bligh	Folch		7983442	PC(36:4)	M+H, M+N	C44H80N(	40,1	4624,117	4252,492	4598,193	4845,093	4365,791	4845,093	95,44%	87,77%	94,90%	100,00%	90,11%
18.92_795.5785n	GP01010073	PC(37:4)	796,5858	1	18,92257	0,808933	0,04557	1,13641	Bligh	MMC	x	GP01010(	PC(37:4)	M+H, M+N	C45H82N(	58,5	889,5137	852,9716	887,3434	950,6922	836,5749	950,6922	93,56%	89,72%	93,34%	100,00%	88,00%
19.49_809.5941n	GP01010077	PC(38:4)	810,6013	1	19,48647	1,679367	0,211694	1,131418	Bligh	MMC	x	GP01010(	PC(38:4)	M+H, M+N	C46H84N(	51,3	18574,49	19039,96	18547,54	20868,67	18444,7	20868,67	89,01%	91,24%	88,88%	100,00%	88,38%
21.91_866.6633m/z	123061285	PC(42:4)	866,6633	1	21,90537	0,241683	0,609485	1,208124	Bligh	MMC		1,23E+08	PC(42:4)	M+H	C50H92N(	35,7	17,41505	15,82123	16,42645	17,63662	14,59835	17,63662	98,74%	89,71%	93,14%	100,00%	82,77%
21.49_866.6621m/z	GP01010081	PC(42:4)	866,6621	1	21,48597	0,30875	0,05249	1,336618	Pooled	MMC		GP01010(	PC(42:4)	M+H	C50H92N(	50	21,68652	18,38374	19,78146	19,52167	16,22492	21,68652	100,00%	84,77%	91,22%	90,02%	74,82%
17.00_766.5386m/z	123060713	PC(35:5)	766,5386	1	17,00393	0,284767	1,34E-05	1,224256	Pooled	Folch		1,23E+08	PC(35:5)	M+H	C43H76N(	41,8	72,15425	58,93725	72,09969	68,15246	63,52456	72,15425	100,00%	81,68%	99,92%	94,45%	88,04%
17.64_779.5475n	GP01010086	PC(36:5)	780,5548	1	17,63515	1,07225	0,000849	1,189507	Bligh	MMC	x	GP01010(	PC(36:5)	M+H, M+N	C44H78N(	51,1	7553,679	7383,19	7665,713	8558,275	7194,808	8558,275	88,26%	86,27%	89,57%	100,00%	84,07%
18.10_794.5704m/z	GP01010089	PC(37:5)	794,5704	1	18,10312	0,661033	0,003216	1,150914	Bligh	Folch		GP01010(	PC(37:5)	M+H	C45H80N(	48	218,7501	200,5021	217,8801	230,7606	200,5097	230,7606	94,80%	86,89%	94,42%	100,00%	86,89%
18.55_807.5788n	GP01010091	PC(38:5)	808,5861	1	18,54758	0,7091	0,267586	1,113592	Bligh	MMC	x	GP01010(	PC(38:5)	M+H, M+N	C46H82N(	56,9	11296,35	11030,06	11332,17	12020,84	10794,65	12020,84	93,97%	91,76%	94,27%	100,00%	89,80%
18.96_822.6011m/z	GP01010094	PC(39:5)	822,6011	1	18,96173	0,520633	3,67E-05	1,190071	Bligh	MMC		GP01010(	PC(39:5)	M+H	C47H84N(	46,2	80,16477	78,58896	78,61086	88,44847	74,32199	88,44847	90,63%	88,85%	88,88%	100,00%	84,03%
19.82_835.6094n	GP01010095	PC(40:5)	836,6167	1	19,81713	0,654767	0,003311	1,117814	Bligh	MMC	x	GP01010(	PC(40:5)	M+H, M+N	C48H86N(	53,9	3327,518	3163,882	3349,045	3438,605	3076,186	3438,605	96,77%	92,01%	97,40%	100,00%	89,46%
22.06_892.6783m/z	123061381	PC(44:5)	892,6783	1	22,058	0,312533	0,053528	1,187389	Pooled	MMC		1,23E+08	PC(44:5)	M+H	C52H94N(	35,8	22,34808	20,23427	18,93052	22,34769	18,8212	22,34808	100,00%	90,54%	84,71%	100,00%	84,22%
18.78_820.5857m/z	GP01010108	PC(39:6)	820,5857	1	18,78125	0,572017	0,107651	1,150308	Bligh	MMC	x	GP010101	PC(39:6)	M+H	C47H82N(	58,5	202,2122	215,8212	201,3574	228,5156	198,656	228,5156	88,49%	94,44%	88,12%	100,00%	86,93%
19.40_833.5944n	GP01010111	PC(40:6)	834,6016	1	19,40015	1,039917	0,224361	1,114776	Bligh	Pooled	x	GP010101	PC(40:6)	M+H, M+N	C48H84N(	58,7	6694,906	6764,415	6921,949	7463,321	6736,743	7463,321	89,70%	90,64%	92,75%	100,00%	90,26%
21.20_876.6477m/z	123061409	PC(43:6)	876,6477	1	21,19673	0,4624	0,140596	1,101645	Bligh	MMC		1,23E+08	PC(43:6)	M+H	C51H90N(	32,8	84,90826	82,15835	84,22762	89,79386	81,50886	89,79386	94,56%	91,50%	93,80%	100,00%	90,77%
17.31_803.5468n	GP01010113	PC(38:7)	804,5541	1	17,30612	0,275533	0,000613	1,421532	Bligh	MTBE		GP010101	PC(38:7)	M+H, M+N	C46H78N(	52	302,4806	333,3925	291,0835	413,7845	299,8353	413,7845	73,10%	80,57%	70,35%	100,00%	72,46%
17.09_803.5470n	GP01010113	PC(38:7)	804,5542	1	17,0881	0,241867	0,050911	1,222654	Bligh	MMC		GP010101	PC(38:7)	M+H, M+N	C46H78N(	45,6	215,3396	189,2947	198,3257	216,6562	177,2016	216,6562	99,39%	87,37%	91,54%	100,00%	81,79%
18.47_831.5787n	GP01010116	PC(40:7)	832,586	1	18,47467	0,958017	0,001036	1,169151	Bligh	MMC	x	GP010101	PC(40:7)	M+H, M+N	C48H82N(	50,2	1077,888	1045,707	1064,149	1188,326	1016,4	1188,326	90,71%	88,00%	89,55%	100,00%	85,53%
17.72_830.5693m/z	GP01010119	PC(40:8)	830,5693	1	17,71985	0,65445	1,69E-05	1,17732	Bligh	MMC		GP010101	PC(40:8)	M+H	C48H80N(	47,7	213,3046	202,8167	217,1502	238,3128	202,4197	238,3128	89,51%	85,11%	91,12%	100,00%	84,94%
17.59_854.5707m/z	GP01010124	PC(42:10)	854,5707	1	17,58832	0,609267	0,003842	1,318027	MTBE	Folch		GP010101	PC(42:10)	M+H	C50H80N(	51,7	46,82614	36,13221	47,62324	44,91384	43,98646	47,62324	98,33%	75,87%	100,00%	94,31%	92,36%
19.67_720.5910m/z	GP01020001	PC(O-32:0)	720,591	1	19,6738	0,473883	0,092712	1,237679	Bligh	MMC	x	GP01020(	PC(O-32:0)	M+H	C40H82N(	53,3	308,0462	317,3081	304,9136	349,3852	282,2907	349,3852	88,17%	90,82%	87,27%	100,00%	80,80%
20.90_747.6147n	GP01020002	PC(O-34:0)	748,622	1	20,9044	0,4068	0,058256	1,353344	Bligh	MMC	x	GP01020(	PC(O-34:0)	M+H, M+N	C42H86N(	55	102,2389	100,345	99,11292	123,1238	90,97743	123,1238	83,04%	81,50%	80,50%	100,00%	73,89%
19.44_718.5753m/z	GP01020004	PC(O-32:1)//PC(P-32:0)	718,5753	1	19,4432	0,444383	0,242997	1,137002	Bligh	MMC		GP01020(	PC(O-32:1)//PC(P-32:0)	M+H	C40H80N(	58,1	184,1759	194,5135	183,4751	195,9199	172,3128	195,9199	94,01%	99,28%	93,65%	100,00%	87,95%
19.92_745.5989n	GP01020005	PC(O-34:1)//PC(P-34:0)	746,6061	1	19,92128	0,67425	0,389321	1,199377	Bligh	MMC		GP01020(	PC(O-34:1)//PC(P-34:0)	M+H, M+N	C42H84N(	65,2	1905,154	1857,024	1941,904	2023,485	1687,113	2023,485	94,15%	91,77%	95,97%	100,00%	83,38%
21.13_774.6363m/z	GP01020006	PC(O-36:1)//PC(P-34:0)	774,6363	1	21,13432	0,581183	0,002148	1,150515	Bligh	MMC	x	GP01020(	PC(O-36:1)//PC(P-34:0)	M+H	C44H88N(	52,4	191,9874	176,5647	191,7377	195,7088	170,1054	195,7088	98,10%	90,22%	97,97%	100,00%	86,92%
23.33_830.6999m/z	GP01020008	PC(O-40:1)//PC(P-40:0)	830,6999	1	23,3315	0,32955	0,184622	1,172301	Bligh	Folch	x	GP01020(	PC(O-40:1)//PC(P-40:0)	M+H	C48H96N(	52,4	85,83769	77,20765	82,20924	90,51058	78,45888	90,51058	94,84%	85,30%	90,83%	100,00%	86,68%
19.09_744.5908m/z	GP01020009	PC(O-34:2)//PC(P-34:1)	744,5908	1	19,08998	0,450033	0,46437	1,122632	Bligh	MMC		GP01020(	PC(O-34:2)//PC(P-34:1)	M+H	C42H82N(	59,2	367,6313	372,1227	369,7801	390,2382	347,6101	390,2382	94,21%	95,36%	94,76%	100,00%	89,08%
19.79_743.5839n	GP01020010	PC(O-34:2)//PC(P-34:1)	744,5911	1	19,7858	0,461	0,009622	1,11481	Bligh	Folch		GP01020(	PC(O-34:2)//PC(P-34:1)	M+H, M+N	C42H82N(	49,5	608,9434	586,6271	625,5163	653,9779	589,9418	653,9779	93,11%	89,70%	95,65%	100,00%	90,21%
20.16_772.6216m/z	GP01020011	PC(O-36:2)//PC(P-36:1)	772,6216	1	20,16463	0,697467	0,000647	1,158345	Bligh	MMC		GP01020(	PC(O-36:2)//PC(P-36:1)	M+H	C44H86N(	64,4	507,5217	478,2711	485,2285	544,0696	469,6957	544,0696	93,28%	87,91%	89,18%	100,00%	86,33%
20.95_771.6145n	GP01020013	PC(O-36:2)//PC(P-36:1)	772,6217	1	20,95378	0,438117	0,231194	1,135591	Bligh	MMC		GP01020(	PC(O-36:2)//PC(P-36:1)	M+H, M+N	C44H86N(	49,9	160,0345	147,7359	162,233	165,7022	145,9171	165,7022	96,58%	89,16%	97,91%	100,00%	88,06%
22.68_828.6848m/z	123061475	PC(O-40:2)//PC(P-40:1)	828,6848	1	22,68118	0,236467	0,591073	1,204388	Bligh	Folch		1,23E+08	PC(O-40:2)//PC(P-40:1)	M+H	C48H94N(	34,8	26,26802	24,82374	28,05499	29,89741	25,12396	29,89741	87,86%	83,03%	93,84%	100,00%	84,03%
20.75_798.6375m/z	GP01020020	PC(O-38:3)//PC(P-38:2)	798,6375	1	20,74695	0,307767	0,013444	1,153637	Bligh	Folch	x	GP01020(	PC(O-38:3)//PC(P-38:2)	M+H	C46H88N(	51,1	97,16368	88,86727	99,16716	102,5206	90,15099	102,5206	94,77%	86,68%	96,73%	100,00%	87,93%
19.83_796.6213m/z	GP01020024	PC(O-38:4)//PC(P-38:3)	796,6213	1	19,83273	0,290567	0,0216	1,197444	Bligh	Folch		GP01020(	PC(O-38:4)//PC(P-38:3)	M+H	C46H86N(	56,2	301,1117	260,8567	294,3879	312,3613	269,2005	312,3613	96,40%	83,51%	94,25%	100,00%	86,18%
19.73_782.6047m/z	7983844	PC(O-37:4)//PC(P-37:3)	782,6047	1	19,73488	0,357467	0,008124	1,195765	Bligh	Folch		7983844	PC(O-37:4)//PC(P-37:3)	M+H	C45H84N(	34,3	43,6138	40,97041	42,14526	48,99099	41,32886	48,99099	89,02%	83,63%	86,03%	100,00%	84,36%
20.25_795.6150n	GP01020026	PC(O-38:4)//PC(P-38:3)	796,6223	1	20,25408	0,905933	0,0058	1,168607	Bligh	MMC		GP01020(	PC(O-38:4)//PC(P-38:3)	M+H, M+N	C46H86N(	54,5	1875,625	1866,09	1871,713	2057,353	1760,517	2057,353	91,17%	90,70%	90,98%	100,00%	85,57%
21.49_823.6474n	GP01020027	PC(O-40:4)//PC(P-40:3)	824,6547	1	21,4937	0,4242	0,555614	1,121194	Bligh	MMC	x	GP01020(	PC(O-40:4)//PC(P-40:3)	M+H, M+N	C48H90N(	52,7	345,354	324,9529	336,9133	352,3847	314,2942	352,3847	98,00%	92,22%	95,61%	100,00%	89,19%
22.02_852.6838m/z	GP01020028	PC(O-42:4)//PC(P-42:3)	852,6838	1	22,0193	0,577817	0,001296	1,17894	Pooled	MMC	x	GP01020(	PC(O-42:4)//PC(P-42:3)	M+H	C50H94N(	52,4	140,9335	126,4158	131,1515	136,5887	119,5425	140,9335	100,00%	89,70%	93,06%	96,92%	84,82%
22.63_851.6779n	GP01020029	PC(O-42:4)//PC(P-42:3)	852,6852	1	22,63498	0,620983	0,713083	1,114225	Bligh	MMC		GP01020(	PC(O-42:4)//PC(P-42:3)	M+H, M+N	C50H94N(	46	509,6853	516,701	486,9094	526,9692	472,9469	526,9692	96,72%	98,05%	92,40%	100,00%	89,75%
23.19_879.7075n	GP01020030	PC(O-44:4)//PC(P-44:3)	880,7148	1	23,19103	0,5022	0,000488	1,364893	Bligh	Folch	x	GP01020(	PC(O-44:4)//PC(P-44:3)	M+H, M+N	C52H98N(	52,9	194,3301	144,5201	159,2303	197,2545	165,0612	197,2545	98,52%	73,27%	80,72%	100,00%	83,68%
23.72_880.7145m/z	GP01020031	PC(O-44:4)//PC(P-44:3)	880,7145	1	23,71565	0,320983	0,072997	1,233115	Pooled	Folch		GP01020(	PC(O-44:4)//PC(P-44:3)	M+H	C52H98N(	48,2	156,3078	126,7585	136,2509	141,7735	130,6051	156,3078	100,00%	81,10%	87,17%	90,70%	83,56%
18.83_765.5682n	GP01020032	PC(O-36:5)//PC(P-36:4)	766,5755	1	18,82507	0,956633	0,217805	1,13362	Bligh	Pooled	x	GP01020(	PC(O-36:5)//PC(P-36:4)	M+H, M+N	C44H80N(	54,2	1244,886	1262,337	1272,098	1411,227	1276,795	1411,227	88,21%	89,45%	90,14%	100,00%	90,47%
19.30_793.5994n	GP01020033	PC(O-38:5)//PC(P-38:4)	794,6067	1	19,29592	0,569167	0,038819	1,097573	Bligh	Folch	x	GP01020(	PC(O-38:5)//PC(P-38:4)	M+H, M+N	C46H84N(	55,7	2827,27	2604,261	2708,012	2858,366	2613,198	2858,366	98,91%	91,11%	94,74%	100,00%	91,42%
21.62_849.6625n	GP01020039	PC(O-42:5)//PC(P-42:4)	850,6698	1	21,6208	0,7552	0,215569	1,139938	Bligh	MMC	x	GP01020(	PC(O-42:5)//PC(P-42:4)	M+H, M+N	C50H92N(	57,7	831,2509	774,0506	781,6441	871,6419	764,6399	871,6419	95,37%	88,80%	89,67%	100,00%	87,72%
22.72_877.6927n	GP01020040	PC(O-44:5)//PC(P-44:4)	878,7	1	22,71603	0,958067	0,260887	1,198026	Bligh	Folch	x	GP01020(	PC(O-44:5)//PC(P-44:4)	M+H, M+N	C52H96N(	55,7	1624,651	1547,143	1726,973	1853,517	1774,123	1853,517	87,65%	83,47%	93,17%	100,00%	95,72%
23.53_906.7300m/z	GP01020041	PC(O-46:5)//PC(P-46:4)	906,73	1	23,52898	0,242983	0,095915	1,268912	Pooled	Folch	x	GP01020(	PC(O-46:5)//PC(P-46:4)	M+H	C54H100N	52,7	70,75137	55,75752	67,98168	59,54648	55,85343	70,75137	100,00%	78,81%	96,09%	84,16%	78,94%
18.55_791.5813n	GP01020042	PC(O-38:6)//PC(P-38:5)	792,5886	1	18,54758	0,35715	0,165601	1,13656	Bligh	MMC		GP01020(	PC(O-38:6)//PC(P-38:5)	M+H, 2M+	C46H82N(	49,6	156,4736	156,5388	153,7856	167,1999	147,1104	167,1999	93,58%	93,62%	91,98%	100,00%	87,98%
20.83_847.6464n	GP01020045	PC(O-42:6)//PC(P-42:5)	848,6537	1	20,83495	0,767067	4,32E-05	1,155128	Bligh	MMC		GP01020(	PC(O-42:6)//PC(P-42:5)	M+H, M+N	C50H90N(	49,8	242,9605	226,7233	242,4439	258,0512	223,3963	258,0512	94,15%	87,86%	93,95%	100,00%	86,57%
21.46_848.6527m/z	GP01020046	PC(O-42:6)//PC(P-42:5)	848,6527	1	21,46268	0,424067	0,002681	1,156701	Bligh	Folch		GP01020(	PC(O-42:6)//PC(P-42:5)	M+H	C50H90N(	45,6	101,3188	98,00554	99,44805	113,3631	100,65	113,3631	89,38%	86,45%	87,73%	100,00%	88,79%
21.95_875.6772n	GP01020047	PC(O-44:6)//PC(P-44:5)	876,6845	1	21,94667	0,778133	0,310285	1,191558	Bligh	MMC	x	GP01020(	PC(O-44:6)//PC(P-44:5)	M+H, M+N	C52H94N(	55,4	648,0606	618,9287	649,739	689,9344	579,0187	689,9344	93,93%	89,71%	94,17%	100,00%	83,92%
22.46_876.6836m/z	GP01020048	PC(O-44:6)//PC(P-44:5)	876,6836	1	22,46377	0,614533	0,033029	1,115062	Bligh	MMC	x	GP01020(	PC(O-44:6)//PC(P-44:5)	M+H	C52H94N(	50,7	176,4907	162,1113	170,4974	177,9679	159,6036	177,9679	99,17%	91,09%	95,80%	100,00%	89,68%
22.97_904.7152m/z	GP01020049	PC(O-46:6)//PC(P-46:5)	904,7152	1	22,97395	0,669717	0,027434	1,261701	Bligh	Folch		GP01020(	PC(O-46:6)//PC(P-46:5)	M+H	C54H98N(	48,1	236,3992	214,6459	237,5405	270,8189	242,9458	270,8189	87,29%	79,26%	87,71%	100,00%	89,71%
18.72_789.5673n	GP01020050	PC(O-38:7)//PC(P-38:6)	790,5746	1	18,71842	0,234267	0,230369	1,10078	Bligh	Folch	x	GP01020(	PC(O-38:7)//PC(P-38:6)	M+H, M+N	C46H80N(	50,7	221,3128	220,4628	233,5869	242,6809	226,1444	242,6809	91,19%	90,84%	96,25%	100,00%	93,19%
19.13_818.6056m/z	GP01020052	PC(O-40:7)//PC(P-40:6)	818,6056	1	19,13457	0,7613	0,276423	1,199713	Bligh	MMC	x	GP01020(	PC(O-40:7)//PC(P-40:6)	M+H	C48H84N(	57	208,0213	226,2819	215,5069	243,1364	202,6621	243,1364	85,56%	93,07%	88,64%	100,00%	83,35%
19.94_817.5980n	GP01020053	PC(O-40:7)//PC(P-40:6)	818,6053	1	19,9368	0,480483	0,306159	1,238106	Bligh	MMC	x	GP01020(	PC(O-40:7)//PC(P-40:6)	M+H, M+N	C48H84N(	52,2	163,8155	167,0092	175,5654	182,2559	147,2055	182,2559	89,88%	91,63%	96,33%	100,00%	80,77%
22.55_901.6914n	GP01020055	PC(O-46:7)//PC(P-46:6)	902,6987	1	22,55157	0,554	0,470509	1,095322	Pooled	MMC	x	GP01020(	PC(O-46:7)//PC(P-46:6)	M+H, M+N	C54H96N(	53,5	249,3989	235,0947	229,7365	245,0397	227,6946	249,3989	100,00%	94,26%	92,12%	98,25%	91,30%
18.72_718.5746m/z	GP01030002	PC(P-32:0)//PC(O-32:1)	718,5746	1	18,71842	0,370933	0,752071	1,058874	MTBE	Folch		GP01030(	PC(P-32:0)//PC(O-32:1)	M+H	C40H80N(	51,6	80,03465	77,4601	82,02047	80,03218	78,78454	82,02047	97,58%	94,44%	100,00%	97,58%	96,05%
18.50_716.5584m/z	GP01030005	PC(P-32:1)//PC(O-32:2)	716,5584	1	18,50465	0,314083	0,237085	1,099213	Bligh	MMC	x	GP01030(	PC(P-32:1)//PC(O-32:2)	M+H	C40H78N(	54,1	49,75677	47,36301	47,56991	51,21976	46,59673	51,21976	97,14%	92,47%	92,87%	100,00%	90,97%
23.83_856.7156m/z	160780063	PC(P-42:1)//PC(O-42:2)	856,7156	1	23,82595	0,279433	0,040569	1,475725	Bligh	MMC		1,61E+08	PC(P-42:1)//PC(O-42:2)	M+H	C50H98N(	35,8	51,59821	41,135	46,81603	54,46681	36,90852	54,46681	94,73%	75,52%	85,95%	100,00%	67,76%
18.92_741.5681n	GP01030011	PC(P-34:2)//PC(O-34:3)	742,5754	1	18,92257	0,4714	0,329489	1,094956	Bligh	MMC		GP01030(	PC(P-34:2)//PC(O-34:3)	M+H, M+N	C42H80N(	52	964,1855	924,0354	960,5984	993,7886	907,6062	993,7886	97,02%	92,98%	96,66%	100,00%	91,33%
20.16_769.5980n	GP01030012	PC(P-36:2)//PC(O-36:3)	770,6052	1	20,16463	0,53945	0,211371	1,108674	Bligh	MMC		GP01030(	PC(P-36:2)//PC(O-36:3)	M+H, M+N	C44H84N(	42,8	361,5886	346,2028	355,4003	381,3739	343,9909	381,3739	94,81%	90,78%	93,19%	100,00%	90,20%
18.99_767.5836n	GP01030014	PC(P-36:3)//PC(O-36:4)	768,5909	1	18,99317	0,6618	0,032008	1,126047	Bligh	Folch		GP01030(	PC(P-36:3)//PC(O-36:4)	M+H, M+N	C44H82N(	50,3	2425,161	2378,686	2520,26	2678,511	2395,222	2678,511	90,54%	88,81%	94,09%	100,00%	89,42%
18.12_764.5597m/z	GP01030021	PC(P-36:5)//PC(O-36:6)	764,5597	1	18,11868	0,347483	0,119202	1,14352	Bligh	Folch		GP01030(	PC(P-36:5)//PC(O-36:6)	M+H	C44H78N(	53	98,53355	91,67536	97,75686	104,8326	95,06727	104,8326	93,99%	87,45%	93,25%	100,00%	90,68%
20.16_819.6144n	GP01030023	PC(P-40:5)//PC(O-40:6)	820,6217	1	20,16463	0,732433	0,031273	1,141065	Bligh	MMC		GP01030(	PC(P-40:5)//PC(O-40:6)	M+H, M+N	C48H86N(	53,8	334,059	329,0383	336,1174	364,8631	319,7565	364,8631	91,56%	90,18%	92,12%	100,00%	87,64%
6.32_468.3092m/z	GP01050001	LPC(14:0)	468,3092	1	6,3187	0,130717	2,39E-10	3,838401	MMC	Bligh	x	GP01050(	LPC(14:0)	M+H	C22H46N(	50,6	22,54881	18,78763	13,59571	6,554399	25,15841	25,15841	89,63%	74,68%	54,04%	26,05%	100,00%
6.72_467.3014n	GP01050002	LPC(14:0)	468,309	1	6,7242	0,4175	4,61E-14	2,386676	MMC	Bligh	x	GP01050(	LPC(14:0)	M+H-H2O	C22H46N(	55,7	165,173	169,8358	168,4124	90,94455	217,0551	217,0551	76,10%	78,25%	77,59%	41,90%	100,00%
7.83_481.3174n	GP01050003	LPC(15:0)	482,3247	1	7,825467	0,432617	8,58E-09	2,241804	MMC	Bligh	x	GP01050(	LPC(15:0)	M+H, M+N	C23H48N(	51,2	182,8337	207,4094	156,1914	95,47249	214,0306	214,0306	85,42%	96,91%	72,98%	44,61%	100,00%

8.42_495.3333n	GP01050004	LPC(16:0)	496,3406	1	8,423983	0,49905	2,04E-05	1,533888	MMC	Bligh	x	GP01050(	LPC(16:0)	M+H-H2O	C24H50N(	57,1	2514,09	2715,071	1966,86	1874,065	2874,605	2874,605	87,46%	94,45%	68,42%	65,19%	100,00%
8.93_495.3333n	GP01050005	LPC(16:0)	496,3406	1	8,926233	0,863133	3,59E-10	1,885618	MMC	Bligh		GP01050(	LPC(16:0)	M+H-H2O	C24H50N(	48,7	9313,095	10970,04	8776,287	6947,42	13100,18	13100,18	71,09%	83,74%	66,99%	53,03%	100,00%
9.99_509.3489n	GP01050007	LPC(17:0)	510,3562	1	9,991033	1,07225	4,06E-09	1,936984	MMC	Bligh	x	GP01050(	LPC(17:0)	M+H-H2O	C25H52N(	53,5	2392,566	2247,658	2317,495	1586,075	3072,202	3072,202	77,88%	73,16%	75,43%	51,63%	100,00%
10.54_523.3646n	GP01050008	LPC(18:0)	524,3717	1	10,54388	0,4438	2,82E-06	1,735094	MMC	Bligh	x	GP01050(	LPC(18:0)	M+H-H2O	C26H54N(	56,4	978,8564	1113,242	1041,74	752,3317	1305,366	1305,366	74,99%	85,28%	79,80%	57,63%	100,00%
11.01_523.3646n	GP01050009	LPC(18:0)	524,3719	1	11,01473	0,6847	1,03E-12	1,665345	MMC	Bligh	x	GP01050(	LPC(18:0)	M+H, M+N	C26H54N(	59,5	5023,98	5405,574	4882,423	3729,859	6211,502	6211,502	80,88%	87,03%	78,60%	60,05%	100,00%
12.01_538.3874m/z	GP01050010	LPC(19:0)	538,3874	1	12,00742	0,169083	1,11E-15	3,269936	MMC	Bligh		GP01050(	LPC(19:0)	M+H	C27H56N(	49,4	40,51939	24,32879	32,44789	19,2385	62,90867	62,90867	64,41%	38,67%	51,58%	30,58%	100,00%
12.93_552.4028m/z	GP01050011	LPC(20:0)	552,4028	1	12,93417	0,227217	5,47E-08	2,254917	MTBE	Bligh		GP01050(	LPC(20:0)	M+H	C28H58N(	46,4	32,61694	29,9964	40,47809	17,95103	33,93017	40,47809	80,58%	74,11%	100,00%	44,35%	83,82%
15.91_608.4659m/z	7983953	LPC(24:0)	608,4659	1	15,90697	0,172067	0,002918	1,416539	Pooled	Folch		7983953	LPC(24:0)	M+H	C32H66N(	36,6	19,35408	13,66294	17,60682	13,82363	14,8596	19,35408	100,00%	70,59%	90,97%	71,42%	76,78%
7.35_493.3175n	GP01050014	LPC(16:1)	494,3248	1	7,350567	0,76035	0	2,561073	MMC	Bligh	x	GP01050(	LPC(16:1)	M+H-H2O	C24H48N(	58	1254,349	1201,936	1022,333	609,4352	1560,808	1560,808	80,37%	77,01%	65,50%	39,05%	100,00%
8.42_508.3404m/z	GP01050015	LPC(17:1)	508,3404	1	8,423983	0,360883	3,88E-09	2,443172	Folch	Bligh	x	GP01050(	LPC(17:1)	M+H	C25H50N(	54	30,84495	37,77237	21,59247	15,46038	36,36545	37,77237	81,66%	100,00%	57,16%	40,93%	96,28%
11.42_549.3801n	GP01050018	LPC(20:1)	550,3874	1	11,42062	0,309633	1,40E-08	1,704251	MMC	Bligh	x	GP01050(	LPC(20:1)	M+H, M+N	C28H56N(	57,4	193,3034	177,199	194,1883	132,8635	226,4327	226,4327	85,37%	78,26%	85,76%	58,68%	100,00%
7.74_519.3332n	GP01050019	LPC(18:2)	520,3405	1	7,737883	0,43305	3,84E-08	1,541554	MMC	Bligh	x	GP01050(	LPC(18:2)	M+H, M+N	C26H50N(	51,6	806,8661	764,2936	778,9177	658,2189	1014,68	1014,68	79,52%	75,32%	76,76%	64,87%	100,00%
8.18_519.3333n	GP01050020	LPC(18:2)	520,3406	1	8,179783	0,6786	2,39E-08	1,864946	MMC	Bligh		GP01050(	LPC(18:2)	M+H-H2O	C26H50N(	49,5	4241,942	5411,76	4076,084	3174,33	5919,953	5919,953	71,66%	91,42%	68,85%	53,62%	100,00%
10.21_547.3646n	GP01050021	LPC(20:2)	548,3719	1	10,2087	0,323333	4,99E-13	2,416407	MMC	Bligh	x	GP01050(	LPC(20:2)	M+H, M+N	C28H54N(	54	146,4341	111,7835	134,4283	82,13807	198,479	198,479	73,78%	56,32%	67,73%	41,38%	100,00%
7.09_517.3174n	GP01050024	LPC(18:3)	518,3247	1	7,088083	0,493917	0	2,936771	MMC	Bligh		GP01050(	LPC(18:3)	M+H, M+N	C26H48N(	49,4	199,8216	194,0137	171,0589	105,066	308,5548	308,5548	64,76%	62,88%	55,44%	34,05%	100,00%
8.60_546.3560m/z	GP01050025	LPC(20:3)	546,356	1	8,597567	0,4032	1,97E-10	2,521738	MMC	Bligh		GP01050(	LPC(20:3)	M+H	C28H52N(	48,5	86,56136	70,6209	80,61346	50,64207	127,706	127,706	67,78%	55,30%	63,12%	39,66%	100,00%
9.03_545.3485n	GP01050026	LPC(20:3)	546,3558	1	9,034017	0,681367	9,14E-09	2,146314	MMC	MTBE	x	GP01050(	LPC(20:3)	M+H-H2O	C28H52N(	56,4	530,0704	638,3394	442,3104	448,0362	949,3371	949,3371	55,84%	67,24%	46,59%	47,19%	100,00%
7.83_543.3329n	GP01050029	LPC(20:4)	544,3401	1	7,825467	0,347317	7,44E-05	1,767943	MMC	Bligh	x	GP01050(	LPC(20:4)	M+H, M+N	C28H50N(	51,5	241,5648	208,9135	222,6117	178,9522	316,3772	316,3772	76,35%	66,03%	70,36%	56,56%	100,00%
8.21_543.3331n	GP01050030	LPC(20:4)	544,3404	1	8,209583	0,6031	2,18E-07	1,660116	MMC	Bligh		GP01050(	LPC(20:4)	M+H-H2O	C28H50N(	49,3	1203,612	1527,802	1114,684	1016,664	1687,78	1687,78	71,31%	90,52%	66,04%	60,24%	100,00%
9.89_572.3717m/z	GP01050031	LPC(22:4)	572,3717	1	9,892233	0,2572	4,34E-07	2,239538	MMC	Bligh		GP01050(	LPC(22:4)	M+H	C30H54N(	47,9	29,47948	22,33822	27,11886	17,95746	40,21642	40,21642	73,30%	55,55%	67,43%	44,65%	100,00%
7.14_541.3175n	GP01050032	LPC(20:5)	542,3248	1	7,137617	0,389133	3,44E-12	2,105416	MMC	Bligh	x	GP01050(	LPC(20:5)	M+H, M+N	C28H48N(	54,1	423,8176	420,1339	376,2526	279,7418	588,9727	588,9727	71,96%	71,33%	63,88%	47,50%	100,00%
8.84_569.3489n	GP01050035	LPC(22:5)	570,3562	1	8,8442	0,3637	0,034885	1,681355	MMC	Bligh	x	GP01050(	LPC(22:5)	M+H, M+N	C30H52N(	55,4	53,30143	62,65243	65,00152	51,25317	86,17475	86,17475	61,85%	72,70%	75,43%	59,48%	100,00%
7.84_568.3403m/z	GP01050036	LPC(22:6)	568,3403	1	7,840733	0,335217	0,001538	1,736958	MMC	Bligh		GP01050(	LPC(22:6)	M+H	C30H50N(	47,2	57,20242	46,85536	52,6058	39,01876	67,77396	67,77396	84,40%	69,13%	77,62%	57,57%	100,00%
8.23_567.3333n	GP01050037	LPC(22:6)	568,3406	1	8,228967	0,47615	1,48E-06	1,634918	MMC	MTBE	x	GP01050(	LPC(22:6)	M+H, M+N	C30H50N(	51,1	344,8516	414,6134	295,7318	310,5436	483,4973	483,4973	71,32%	85,75%	61,17%	64,23%	100,00%
10.02_481.3536n	GP01060001	LPC(O-16:0)	482,3609	1	10,02158	0,39575	9,74E-10	2,368258	MMC	Bligh		GP01060(	LPC(O-16:0)	M+H, M+N	C24H52N(	47	151,4968	144,7285	137,446	84,68916	200,5658	200,5658	75,53%	72,16%	68,53%	42,23%	100,00%
12.06_509.3850n	GP01060002	LPC(O-18:0)	510,3923	1	12,06317	0,261833	3,20E-06	2,1018	MMC	Bligh		GP01060(	LPC(O-18:0)	M+H, M+N	C26H56N(	48,8	76,34776	69,99172	64,21884	44,99488	94,57026	94,57026	80,73%	74,01%	67,91%	47,58%	100,00%
9.81_479.3383n	GP01070001	LPC(P-16:0)	480,3456	1	9,80955	0,429517	7,90E-07	2,17353	MMC	Bligh	x	GP01070(	LPC(P-16:0)	M+H, M+N	C24H50N(	51,8	408,5159	402,6138	371,3345	196,2111	426,4706	426,4706	95,79%	94,41%	87,07%	46,01%	100,00%
10.51_507.3693n	GP01070002	LPC(P-18:0)//LPC(O-18:1)	508,3766	1	10,51288	0,388433	4,40E-08	2,41285	Folch	Bligh		GP01070(	LPC(P-18:0)//LPC(O-18:1)	M+H, M+N	C26H54N(	51,5	179,7955	294,0497	187,5369	121,8682	271,3526	294,0497	61,14%	100,00%	63,78%	41,44%	92,28%
11.09_506.3611m/z	GP01070004	LPC(P-18:1)//LPC(O-18:2)	506,3611	1	11,09248	0,514983	9,18E-10	2,534338	MMC	Bligh		GP01070(	LPC(P-18:1)//LPC(O-18:2)	M+H	C26H52N(	49,8	135,3305	140,8841	125,4646	70,60778	178,944	178,944	75,63%	78,73%	70,11%	39,46%	100,00%
29.30_624.5852n	24702201	CE(16:0)	642,6184	1	29,30178	0,465167	0,010823	1,318418	Folch	Pooled		24702201	CE(16:0)	M+NH4, M	C43H76O	38,9	818,248	1078,793	979,7188	974,4192	1006,368	1078,793	75,85%	100,00%	90,82%	90,32%	93,29%
31.19_652.6160n	24702203	CE(18:0)	1328,221	1	31,19345	0,629867	0,655091	1,13461	MTBE	MMC		24702203	CE(18:0)	M+Na, 2M	C45H80O	40,5	201,2458	200,9312	213,1061	200,7636	242,7889	242,7889	82,89%	82,76%	87,77%	82,69%	100,00%
27.90_622.5696n	ST01020001	CE(16:1)	670,6497	1	27,90498	0,502267	0,248303	1,2422	Bligh	MMC	x	ST010200	CE(16:1)	M+NH4, M	C43H74O	52	230,5948	274,6216	249,4368	260,3246	263,684	274,6216	83,97%	100,00%	90,83%	94,79%	96,02%
29.56_650.6009n	ST01020002	CE(18:1)	673,5904	1	29,56063	0,778467	0,16616	1,30885	Bligh	MMC		ST010200	CE(18:1)	M+H, M+N	C45H78O	46	958,0571	1052,806	1006,353	1071,851	1009,452	1071,851	89,38%	98,22%	93,89%	100,00%	94,18%
28.17_648.5854n	ST01020003	CE(18:2)	671,5747	1	28,17245	1,311917	0,225692	1,25637	Bligh	MMC	x	ST010200	CE(18:2)	M+H, M+N	C45H76O	53,1	1793,368	1950,118	1816,479	1936,627	1873,72	1950,118	91,96%	100,00%	93,15%	99,31%	96,08%
27.35_646.5698n	ST01020004	CE(18:3)	669,559	1	27,34758	0,442717	0,096351	1,246665	Bligh	MMC	x	ST010200	CE(18:3)	M+H, M+N	C45H74O	53,3	333,1116	384,7541	358,0702	371,9452	361,5154	384,7541	86,58%	100,00%	93,06%	96,67%	93,96%
28.67_674.6011n	ST01020005	CE(20:3)	692,635	1	28,67407	0,44505	0,01326	1,240858	Folch	Pooled	x	ST010200	CE(20:3)	M+H, M+N	C47H78O	51,1	94,56631	117,3433	107,2862	100,6605	113,4412	117,3433	80,59%	100,00%	91,43%	85,78%	96,67%
27.77_672.5854n	ST01020006	CE(20:4)	695,5747	1	27,77478	0,755617	0,104754	1,246877	Bligh	MMC	x	ST010200	CE(20:4)	M+H, M+N	C47H76O	58,3	979,3198	1087,721	1056,601	1081,837	1033,569	1087,721	90,03%	100,00%	97,14%	99,46%	95,02%
27.48_696.5852n	ST01020008	CE(22:6)	714,6191	1	27,47563	0,4619	0,008961	1,212087	Folch	Pooled		ST010200	CE(22:6)	M+H, M+N	C49H76O	57	422,9592	512,6634	484,1177	482,8126	497,2719	512,6634	82,50%	100,00%	94,43%	94,18%	97,00%
28.08_716.6343m/z	ST01020009	CE(22:5)	716,6343	1	28,0759	0,452933	0,202631	1,199569	Folch	Pooled		ST010200	CE(22:5)	M+NH4	C49H78O	62,5	15,19568	18,22826	17,68997	16,23179	17,17325	18,22826	83,36%	100,00%	97,05%	89,05%	94,21%
